# Domain structure and cross-linking in a giant adhesin from the *Mobiluncus mulieris* bacterium

**DOI:** 10.1107/S2059798323007507

**Published:** 2023-10-20

**Authors:** Paul G. Young, Jacob M. Paynter, Julia K. Wardega, Martin J. Middleditch, Leo S. Payne, Edward N. Baker, Christopher J. Squire

**Affiliations:** a School of Biological Sciences, The University of Auckland, Private Bag 92019, Auckland 1010, New Zealand; b Maurice Wilkins Centre for Molecular Biodiscovery, c/o The University of Auckland, Private Bag 92019, Auckland 1010, New Zealand; University of Queensland, Australia

**Keywords:** bacterial adhesins, Ig-like domains, intramolecular cross-links, cell adhesion

## Abstract

Cell-surface proteins known as adhesins enable bacteria to colonize particular environments, and in Gram-positive bacteria often contain autocatalytically formed covalent intramolecular cross-links. While investigating the prevalence of such cross-links, a remarkable example was discovered in *Mobiluncus mulieris*, a pathogen associated with bacterial vaginosis. This organism encodes a putative adhesin of 7651 residues. Crystallography and mass spectrometry of two selected domains, and *AlphaFold* structure prediction of the remainder of the protein, were used to show that this adhesin belongs to the family of thioester, isopeptide and ester-bond-containing proteins (TIE proteins). It has an N-terminal domain homologous to thioester adhesion domains, followed by 51 immunoglobulin (Ig)-like domains containing ester- or isopeptide-bond cross-links. The energetic cost to the *M. mulieris* bacterium in retaining such a large adhesin as a single gene or protein construct suggests a critical role in pathogenicity and/or persistence.

## Abstract 2

An adhesin from *Mobiluncus mulieris*, a bacterium associated with persistence in bacterial vaginosis, contains 51 repeat Ig-like domains. Each domain displays cross-linking including intramolecular ester, isopeptide, disulfide and thioester bonds. This giant 7651-residue protein, by far the largest in the bacterial proteome, is presumably retained because of its critical pathogenic role.

## 1. Introduction

Bacteria occupy innumerable replicative niches, often within hostile environments in the human body. Their interactions with the environment are mediated by their surface structures, which include molecules collectively termed adhesins. These filamentous, ‘sticky’ appendages have critical roles in surface attachment and biofilm formation, and are particularly important in mediating host-cell interactions of pathogenic bacteria (Kline *et al.*, 2009 ▸).

In Gram-positive bacteria, many of the cell-surface proteins are attached covalently to the cell wall by enzymes called sortases. These enzymes recognize a ‘sorting’ motif, typically LP*x*TG, near the C-terminus of the protein, cleave the polypeptide following the threonine residue and join the new C-terminus to the peptidoglycan layer with an isopeptide bond (Marraffini *et al.*, 2006 ▸). A subset of these adhesins, typified by the adhesin from *Streptococcus pyogenes*, take the form of covalent polymers with individual subunits (pilins) covalently linked into chains by sortases that act as pilin polymerases (Kang & Baker, 2012 ▸). Others, typified by the adhesin from *Clostridium perfringens*, comprise a single gene or open reading frame (ORF) that produces an N-terminal adhesion domain followed by a long ‘stalk’, often with multiple repet­itive protein domains arrayed like beads on a string (Patti *et al.*, 1994 ▸; Baker *et al.*, 2015 ▸). In both of these cases the repeat domains are variations on an Ig-like fold, a structure that is extremely versatile and is highly abundant in cell-surface receptors (Chen *et al.*, 2018 ▸).

The discovery of intramolecular isopeptide-bond cross-links in the pilin protein Spy0128, which forms the repetitive polymerized backbone of pili expressed by *S. pyogenes* (Kang *et al.*, 2007 ▸), showed how extremely long and thin surface proteins resist extreme environmental stress. Spy0128 comprises two immunoglobulin (Ig)-like domains, each with a spontaneously formed isopeptide cross-link between lysine and asparagine side chains on adjacent β-strands. Similar bonds have since been found in the pili of many other Gram-positive bacteria (Kang & Baker, 2012 ▸). While investigating their prevalence, we found a second type of covalent cross-link, this time involving ester bonds formed between threonine and glutamine side chains in the repetitive Ig-like domains of the *C. perfringens* adhesin Cpe0147 (Kwon *et al.*, 2014 ▸). This protein comprises a single polypeptide with an N-terminal adhesion domain followed by 11 repeat Ig-like domains.

The intramolecular cross-links, whether isopeptide or ester bonds, stabilize the individual Ig-like domains and provide a covalently linked ‘spine’ the entire length of the protein stretching from the surface of the bacterium to the adhesion domain. Another feature of adhesins of these types is the frequent observation of a third type of post-translational modification: covalent thioester bonds between cysteine and glutamine side chains that provide a reactive ‘warhead’ within the thioester adhesin domains (TEDs) that mediate covalent bacterial adhesion (Walden *et al.*, 2015 ▸). Adhesin surface proteins that combine thioester, isopeptide and ester bonds (TIE proteins) are widespread among Gram-positive bacteria (Miller *et al.*, 2018 ▸).

Despite considerable interest in the chemistry of bond formation (Kwon *et al.*, 2014 ▸; Kang & Baker, 2011 ▸; Hagan *et al.*, 2010 ▸; Hu *et al.*, 2011 ▸) and in the use of these spontaneously formed bonds for applications in synthetic biology and biotechnology (Zakeri & Howarth, 2010 ▸; Young *et al.*, 2017 ▸), the extent of natural structural variation is largely unknown. The 11 sequential ester-bond domains of Cpe0147 show high sequence identity (Kwon *et al.*, 2014 ▸). Following up on this work, we searched sequence databases and identified putative ester-bond-containing adhesins in the proteomes of *Mobiluncus mulieris*, *M. curtisii* and *Varibaculum cambriense*, bacteria that are most often associated with bacterial vaginosis (Spiegel & Roberts, 1984 ▸; Onderdonk *et al.*, 2016 ▸).

Here, we describe the structures of these adhesins, which are remarkably large, comprising single-chain molecules of up to 7651 amino-acid residues in length. We find that these adhesin molecules contain four covalent cross-link types, isopeptide, esther, disulfide and thioester bonds, verifying their presence by mass spectrometry and X-ray crystallo­graphy. This discovery raises intriguing questions as to the roles of these supersized adhesins in pathology and disease.

## 2. Materials and methods

### 2.1. Bioinformatics/structure prediction

The amino-acid sequence motif H*x*D*xx*D*xx*Q, derived from the ester-bond domains of Cpe0147, was submitted to the *BLAST* server and the default *BLASTP* algorithm was used to search nonredundant protein sequences across all organisms. This search identified many putative ester-bond-containing adhesins, including examples from *M. mulieris*, *M. curtisii* and *V. cambriense* (NCBI Reference Sequences WP_004013458.1, WP_013188882.1 and WP_101929469.1, respectively). To enhance these predictions and to search for other domains in these proteins, the amino-acid sequences were submitted to the *AlphaFold*2 server for 3D structure prediction as detailed in Supplementary Tables S1–S3 (Jumper *et al.*, 2021 ▸; Varadi *et al.*, 2022 ▸). The predicted structures were overlaid onto the crystal structures of proteins shown to contain ester bonds (Cpe0147; Kwon *et al.*, 2014 ▸; PDB entry 4ni6), isopeptide bonds (Spy0128; Kang *et al.*, 2007 ▸; PDB entry 3b2m) and thioester bonds (SaTIE; Miller *et al.*, 2018 ▸; PDB entry 6fx6). This allowed us to map putative domain boundaries onto the full-length sequences of the *Mobiluncus* and *Varibaculum* proteins and to classify the domains according to their predicted cross-link types. Multiple sequence alignments were performed using *Clustal Omega* and were visualized using *MView* (Sievers *et al.*, 2011 ▸; Brown *et al.*, 1998 ▸).

### 2.2. Cloning

*M. mulieris* (strain BV 64-5) genomic material was purchased from ATCC (ATCC 35240D5) and the putative adhesin sequence was PCR-amplified using E14 forward (5′-tattttcagggcgccAAGCCTGGAGTGGGCACCTACGCTAC-3′) and I30 reverse (5′-gaattccggatccattcaGTAGCTAAACGAGTTTTCTGCGGTTACTTCGACATTC-3′) primers with 5′ 15-base-pair complementary pProEX HTa vector sequences for In-Fusion cloning (Clontech) (Table 1 ▸). A high annealing temperature of 72°C was chosen to minimize false priming due to the GC-rich sequence of the *M. mulieris* genome. The vector was similarly PCR-amplified with pProEX HTa Fwd (ATGGATCCGGAATTCAAAGGCCTAC) and pProEX HTa Rev (GGCGCCCTGAAAATACAGGTTTTC) primers to produce a linear product that was circularized with the adhesin gene fragment by In-Fusion recombination cloning and transformed into electrocompetent Stellar *Escherichia coli* cells (Clontech). A single colony was transferred into a 12 ml culture tube containing 5 ml 2×YT medium supplemented to 0.1 µg ml^−1^ ampicillin and incubated with shaking at 37°C overnight. The plasmid was extracted and purified from a 1 ml volume of cells using a Nucleospin Plasmid EasyPure kit (Macherey-Nagel).

### 2.3. Protein production

The purified plasmid was used to transform electrocompetent *E. coli* BL21(DE3) cells using standard electroporation protocols. A single colony was cultured overnight in 10 ml 2×YT medium at 37°C and transferred into 2 l baffled culture flasks containing 1 l 2×YT medium supplemented to 0.1 µg ml^−1^ ampicillin. The cultures were grown at 37°C with shaking to an OD_600_ of ∼0.5 before induction with 0.3 m*M* isopropyl β-d-1-thiogalactopyranoside. The culture was transferred to 18°C and incubated with shaking overnight. The cells were resuspended in 30 ml lysis buffer (50 m*M* HEPES–KOH pH 7.5, 500 m*M* NaCl, 10 m*M* imidazole, 2% glycerol) and transferred into 50 ml Falcon tubes before flash-cooling in liquid nitrogen for storage at −20°C.

Selenomethione (SeMet)-substituted protein was produced in a similar manner but with the initial overnight culture in 2×YT medium, centrifuged, resuspended in 1 ml M9 minimal medium and seeded into 2 l baffled culture flasks containing 1 l M9 minimal medium. When the OD_600_ reached ∼0.5, powdered amino acids (100 mg each of lysine, phenylalanine and threonine, 50 mg each of isoleucine, leucine and valine, and 60 mg selenomethionine) were added to the culture, which was then grown at 37°C for a further 15 min to allow inhibition of methionine-biosynthesis pathways. The culture was induced and was transferred to 18°C for 16 h before harvesting as described for the native protein.

### 2.4. Purification

The cell pellets were lysed in an M-110P microfluidizer (Microfluidics). The lysates were clarified by centrifugation at 30 000*g* for 20 min at 4°C and the supernatant was applied onto a 5 ml IMAC column (HiTrap) pre-equilibrated with lysis buffer. Two column volumes of wash buffer (50 m*M* HEPES–KOH pH 7.5, 500 m*M* NaCl, 20 m*M* sodium imidazole, 2% glycerol) were then passed over the column before elution with a buffer comprising 50 m*M* HEPES–NaOH pH 7.0, 300 m*M* NaCl, 500 m*M* sodium imidazole, 2% glycerol.

The polyhistidine tag was cleaved concurrently with buffer exchange by dialysis against 1 l size-exclusion chromatography (SEC) buffer (20 m*M* HEPES–NaOH pH 7.0, 100 m*M* NaCl) supplemented with β-mercaptoethanol to 1 m*M* and recombinant Tobacco etch virus protease (rTEV) at a protein mass ratio of 1:75. After overnight dialysis, the sample was re­applied onto an IMAC column and the eluate was collected and concentrated to 500 µl before application onto a Superdex S200 10/30 size-exclusion column (GE Healthcare Life Sciences) pre-equilibrated with SEC buffer. The eluted protein was concentrated to ∼250 mg ml^−1^ and stored on ice prior to crystallization experiments.

### 2.5. X-ray crystallography

Sitting-drop vapour-diffusion experiments were performed in 96-well plates (Art Robbins Instruments), screening 576 different conditions (Table 2 ▸). Drops of 400 nl (200 nl protein at ∼250 mg ml^−1^ in SEC buffer mixed with 200 nl reservoir solution) were dispensed using an Oryx4 robot (Douglas Instruments) and were equilibrated against 100 µl reservoir solution at 18°C. Several conditions yielded protein crystals, and diffraction-quality crystals were then produced from a hanging-drop fine screen using 1 µl + 1 µl drops in 24-well Linbro Plates (Hampton Research) equilibrated against 500 µl reservoir comprising MORPHEUS screen formulation G2 (Gorrec, 2009 ▸). The optimized condition comprised 10%(*v*/*v*) PEG 8000, 20%(*v*/*v*) ethylene glycol, 0.02 *M* carboxylic acids (sodium formate, ammonium acetate, tri­sodium citrate, sodium potassium tartrate, sodium oxamate) and 0.1 *M* MES–imidazole pH 6.5. SeMet-substituted crystals were produced from the same conditions.

Crystals were mounted in nylon loops directly from the crystallization drops and were flash-cooled in liquid nitrogen. Data were collected on the MX1 and MX2 beamlines at the Australian Synchrotron. Indexing and integration was performed using *XDS* with merging and scaling using *AIMLESS* (Kabsch, 2010 ▸; Evans & Murshudov, 2013 ▸). Details of data collection and processing for two native crystal forms are given in Table 3 ▸.

Experimental phases were obtained by single-wavelength anomalous dispersion (SAD) using SeMet-substituted crystals. A wavelength 342 eV above the theoretical selenium absorption edge (12 658 eV) was chosen for data collection. Diffraction data were obtained as for the native crystals, ensuring sufficient multiplicity for a strong anomalous signal. The processed data were submitted to the *Auto-Rickshaw* web server and a SeMet crystal structure was solved using the automated SAD protocol (Panjikar *et al.*, 2005 ▸). Native structures were solved by molecular replacement using single domains from the SeMet structure as search models in *Phaser* (McCoy *et al.*, 2007 ▸). Both the *P*1 and the *P*2_1_ native structures contained a single, two-domain molecule in the asymmetric unit. Iterative cycles of modelling and real-space refinement in *Coot* (Emsley *et al.*, 2010 ▸), together with maximum-likelihood refinement in *REFMAC*5 (Murshudov *et al.*, 2011 ▸), completed the structures. Final rounds of refinement used full anisotropic modelling of *B* factors where the resolution allowed. Refinement details are provided in Table 4 ▸.

### 2.6. Mass spectrometry

The two-domain protein used for X-ray crystallography was subjected to electrophoresis (SDS–PAGE) and bands were excised from the gel matrix, destained, digested with trypsin (without reduction and alkylation in order to detect disulfide cross-linked peptides) and the acidified digests were diluted fivefold in 0.1% formic acid. A 2 µl aliquot of each digest was desalted on a 0.3 × 10 mm trap column packed with 3 µm Reprosil C18 media (Dr Maisch) before separation on a 0.075 × 200 mm PicoFrit column (New Objective) packed in-house with 3 µm Reprosil C18 medium using a gradient of 0.1% formic acid in water (*A*) and 0.1% formic acid in acetonitrile (*B*) at 250 nl min^−1^: 0 min 1% *B*, 4 min 2% *B*, 22 min 35% *B*, 24 min 90% *B*, 28 min 90% *B*, 28.5 min 1% *B*, 45 min 1% *B*. The PicoFrit spray was directed into a TripleTOF 6600 quadru­pole time-of-flight mass spectrometer (Sciex, Framingham, Massachusetts, USA) scanning from *m*/*z* 350 to 1600 for 150 ms, followed by up to 30 MS/MS scans per cycle (*m*/*z* 100–1600) on multiply charged species using dynamic collision energy. Manual interpretation of the resulting raw data resulted in annotated MS/MS spectra for the three types of cross-linked peptide from the protein described here.

## 3. Results

### 3.1. Bioinformatics and structure prediction identify up to 51 repeat domains in single-protein adhesins

A *BLAST* sequence search identified numerous putative intramolecular ester-bond-containing Ig-like domains containing the signature H*x*D*xx*D*xx*Q sequence motif associated with the reactive (cross-linking) glutamine in Cpe0147 (Kwon *et al.*, 2014 ▸). This search predicted 18 such domains in a hypothetical protein from *M. curtisii*, including a run of 14 tandem domains in the C-terminal half of the sequence. The *M. curtisii* genome sequence (GenBank assembly CP001992.1) shows that this hypothetical protein is the largest in the bacterial proteome, comprising 5040 amino acids (Table 5 ▸). The closely related bacterial species *M. mulieris* (GenBank assembly GCA_000160615.1) contains an even larger 7645-amino-acid protein, similarly the largest gene product in its proteome (Table 5 ▸). The two proteins are homologues, with the *M. curtisii* protein, covering 62% of the *M. mulieris* sequence and sharing 35% sequence identity with it (Supplementary Fig. S1). Both proteins contain cell-wall-anchoring LP*x*TG motifs, identifying them as cell-surface proteins and putative adhesins.

**Figure 1:**
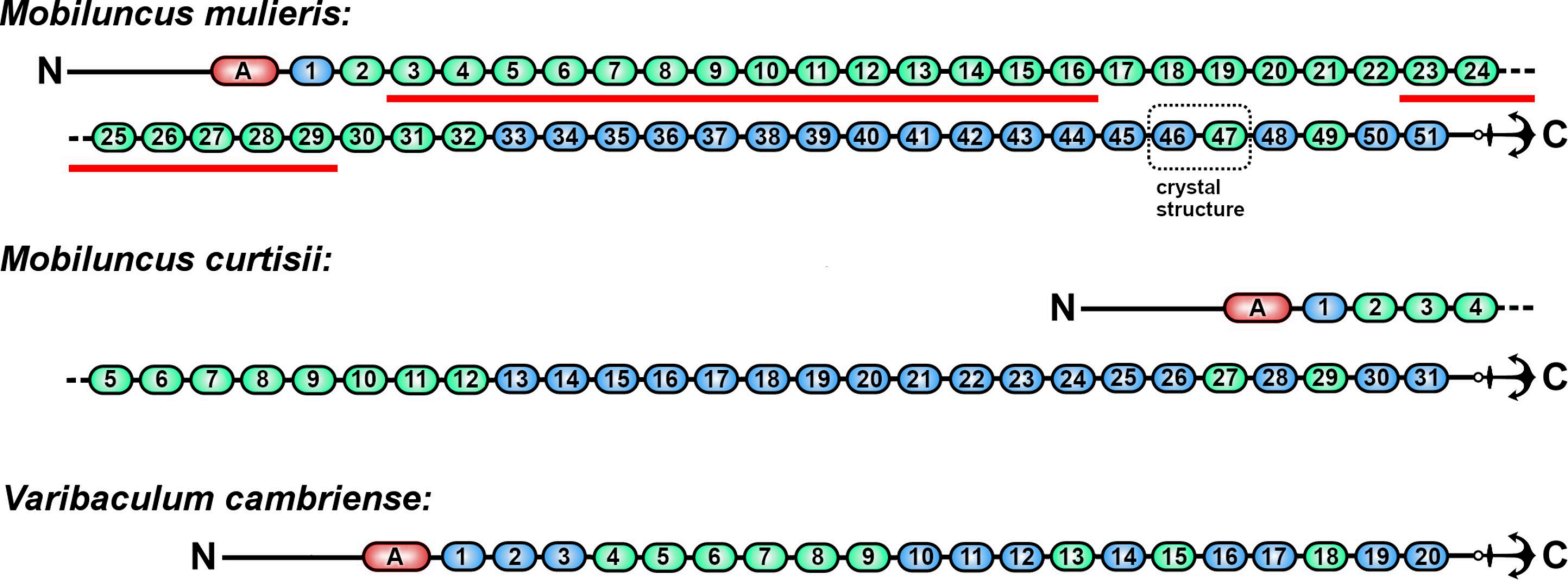
Predicted domain structures of putative adhesins from Actinomyces family bacteria implicated in bacterial vaginosis. Ester-bond-containing domains are coloured blue and isopeptide domains green. N-terminal adhesin (A) and C-terminal LP*x*TG cell-anchoring (anchor graphic) locations are indicated. Domains are numbered from 1 starting at each respective adhesin domain. The *M. mulieris* and *M. curtisii* domain structures and protein sequences match, except for large isopeptide-domain insertions (underlined in *M. mulieris* with a bold red line). The two-domain gene construct encoding repeat domains 46 and 47 was cloned and expressed, and the crystal structure of the purified protein was determined.

The ester-bond cross-link domains within these two proteins were delineated using pairwise amino-acid sequence alignments against domain 2 of Cpe0147, together with manual, multiple sequence alignments focusing on the conserved H*x*D*xx*D*xx*Q motif (Supplementary Fig. S2). Subsequent 3D structure prediction using *AlphaFold*2 (Jumper *et al.*, 2021 ▸; Varadi *et al.*, 2022 ▸) corroborated the existence of 18 ester-bond cross-link domains in both the *M. mulieris* and *M. curtisii* proteins, which were mostly located in the C-terminal half of their respective sequences (Fig. 1 ▸).

To identify other domains in the remaining portions of the *M. mulieris* protein, we searched for additional Ig-like repeats using the amino-acid sequence of the isopeptide-containing C-terminal domain of the *S. pyogenes* pilin protein Spy0128. A combination of pairwise sequence alignments, internal multiple sequence alignment (Supplementary Fig. S3), manual inspection for characteristic sequence motifs and 3D structure prediction highlights the presence of an additional 33 intramolecular isopeptide-containing domains in the *M. mulieris* protein. The *M. curtisii* protein, while having far fewer putative isopeptide domains (13 in total), appears to have a similar distribution of ester-bond and isopeptide domains (Fig. 1 ▸), consistent with a common evolutionary provenance.

Analysis of a further Actinomyces bacterium, *V. cambriense*, also commonly associated with bacterial vaginosis shows a putative adhesin comprised of 20 repeat domains, 11 ester-bond cross-linked and nine isopeptide-bond cross-linked domains. Attempts to predict the structure of the adhesion domain using *AlphaFold* failed (Fig. 1 ▸).

To definitively confirm the predicted combination of cross-link types in the *M. mulieris* protein, we characterized a recombinant two-domain protein construct at the interface between two domain types, comprising the adjacent 15th putative ester-bond domain and 32nd putative isopeptide domain (the construct comprising domains 46E–47I in Fig. 1 ▸).

### 3.2. Mass spectrometry confirms three different intramolecular cross-link types

Definitive proof of the cross-linking chemistry in the two-domain construct was first sought by mass fingerprinting, subjecting the protein to trypsin digestion followed by LC-MS/MS mass-spectrometry analysis (Supplementary Figs. S4–S6). Unique fragments identified from within the mass spectra contain the two predicted cross-link types, an ester-bond cross-link between Thr6674 and Gln6827 and an isopeptide-bond cross-link between Lys6842 and Asn6955, and further identified a disulfide bond linking Cys6887 and Cys6895.

### 3.3. Cross-links revealed by X-ray crystallography

**Figure 2:**
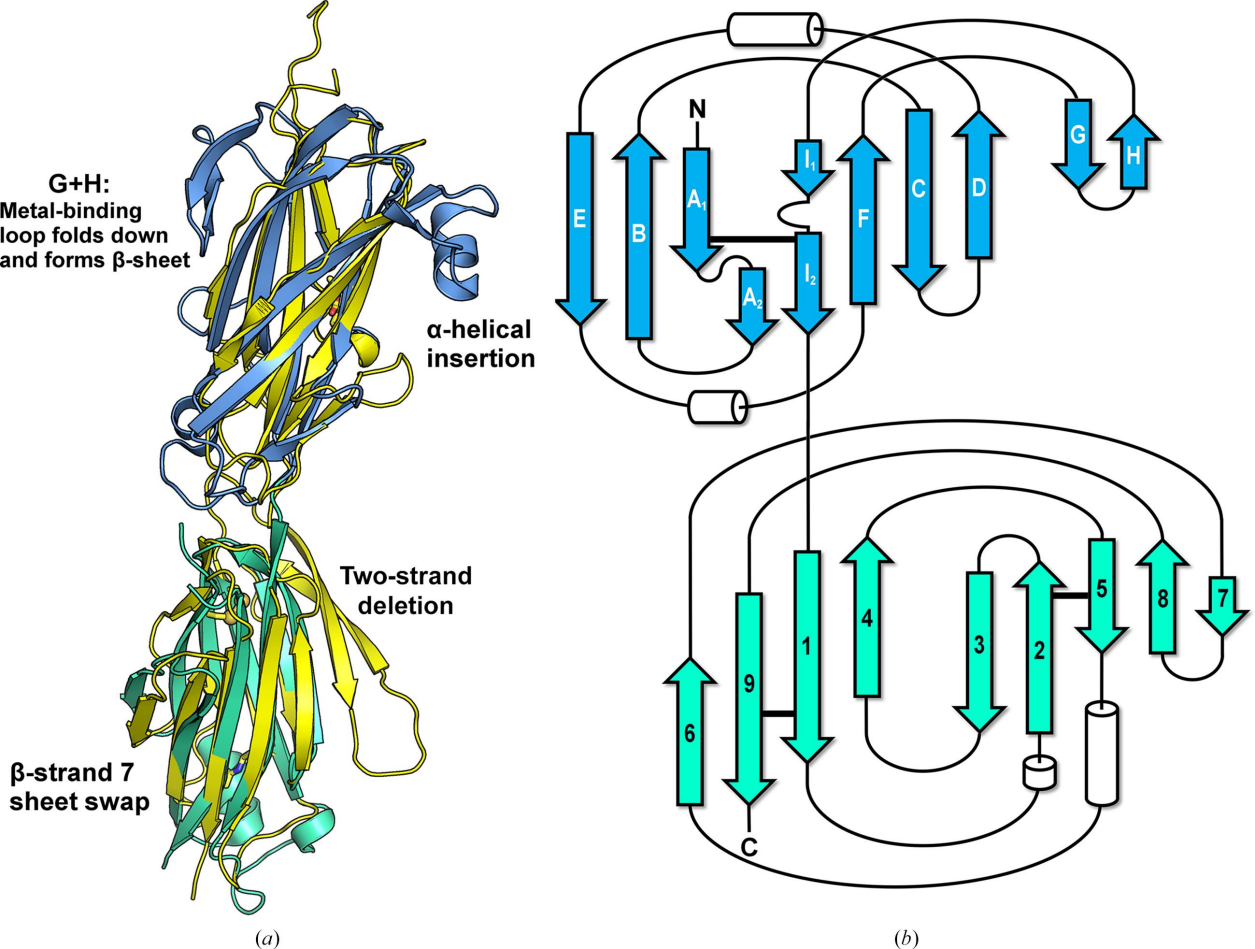
Structure of the two-domain construct 46E–47I from the *M. mulieris* adhesin and its comparison with the Cpe0147 and Spy0128 proteins. (*a*) *M. mulieris* adhesin domains (ester domain in blue; isopeptide domain in green) overlaid with Cpe0147 domain 2 (PDB entry 4ni6; yellow) and Spy0128 adhesin domain 2 (PDB entry 3b2m; yellow). (*b*) Topology diagram of the *M. mulieris* adhesin domains. The N-terminal ester domain is coloured blue with the cross-link shown as a bold horizontal line joining the first and last strands of the domain. The C-terminal isopeptide domain is coloured green with cross-links shown as bold horizontal lines. The isopeptide bond links strands 1 and 9, while the disulfide bond links strands 2 and 5. Each domain topology is similar to either the Cpe0147 or Spy0128 proteins but with four major changes as indicated in (*a*).

Crystal structures of the mixed ester–isopeptide construct 46E–47I were solved in two different space groups. Experimental phases were obtained by SAD with *Auto-Rickshaw* using data from SeMet-substituted crystals (two Se atoms per protein molecule; Panjikar *et al.*, 2005 ▸). The native structure (Fig. 2 ▸) was then solved by molecular replacement using *Phaser* (McCoy *et al.*, 2007 ▸). The molecules of the two space groups differ across 287 aligned C^α^ coordinates by a root-mean-square difference (r.m.s.d.) of 1.78 Å. Each pair of domains aligns more closely (C^α^ of residues 1–168, 0.72 Å r.m.s.d.; C^α^ of residues 169–292, 0.50 Å r.m.s.d.), suggesting that the flexible interdomain linker affords slightly different relative domain orientations. Structure determination and refinement statistics are provided in Tables 3 ▸ and 4 ▸.

The N-terminal 46E ester-bond domain structure closely overlays with the ester-bond domains of *C. perfringens* Cpe0147 (Kwon *et al.*, 2014 ▸), with an r.m.s.d. of 1.88 Å over 108 C^α^ atoms (Fig. 2 ▸). An unambiguous intramolecular ester-bond cross-link is seen to connect Thr6674 on the first β-strand of the domain fold to Gln6827 on the last β-strand. The 46E domain differs from adhesin stalk domain 1 of Cpe0147 only by the presence of an α-helical insertion and by a different orientation of the metal-binding loop. In the 46E domain, this loop folds back onto the protein, forming a two-stranded β-sheet, rather than presenting the extended metal-binding structure seen in Cpe0147.

A short 2–3-amino-acid linker precedes the isopeptide-containing 47I domain. As predicted, an isopeptide bond links the first and last β-strands of this domain, joining Lys6842 to Asn6955. There is clear homology between this domain and the second (smaller) isopeptide domain of the two-domain *S. pyogenes* pilin protein Spy0128 (Fig. 2 ▸). Superposition of the two structures affords an r.m.s.d. of 2.34 Å over 102 C^α^ atoms. The main differences between the 47I domain of *M. mulieris* and domain 2 of Spy0128 are a two-strand deletion from one β-sheet of the β-sandwich and a small additional β-strand (β-strand 7) associated with the opposite β-sheet (Fig. 2 ▸). The disulfide linkage between Cys6887 and Cys6895 identified by mass spectrometry in the 47I domain is not fully formed in the crystal structures, possibly as a result of radiation damage, and is consequently modelled at partial occupancy.

### 3.4. The cross-link environments confirm enzyme-like cross-linking reaction mechanisms

**Figure 3:**
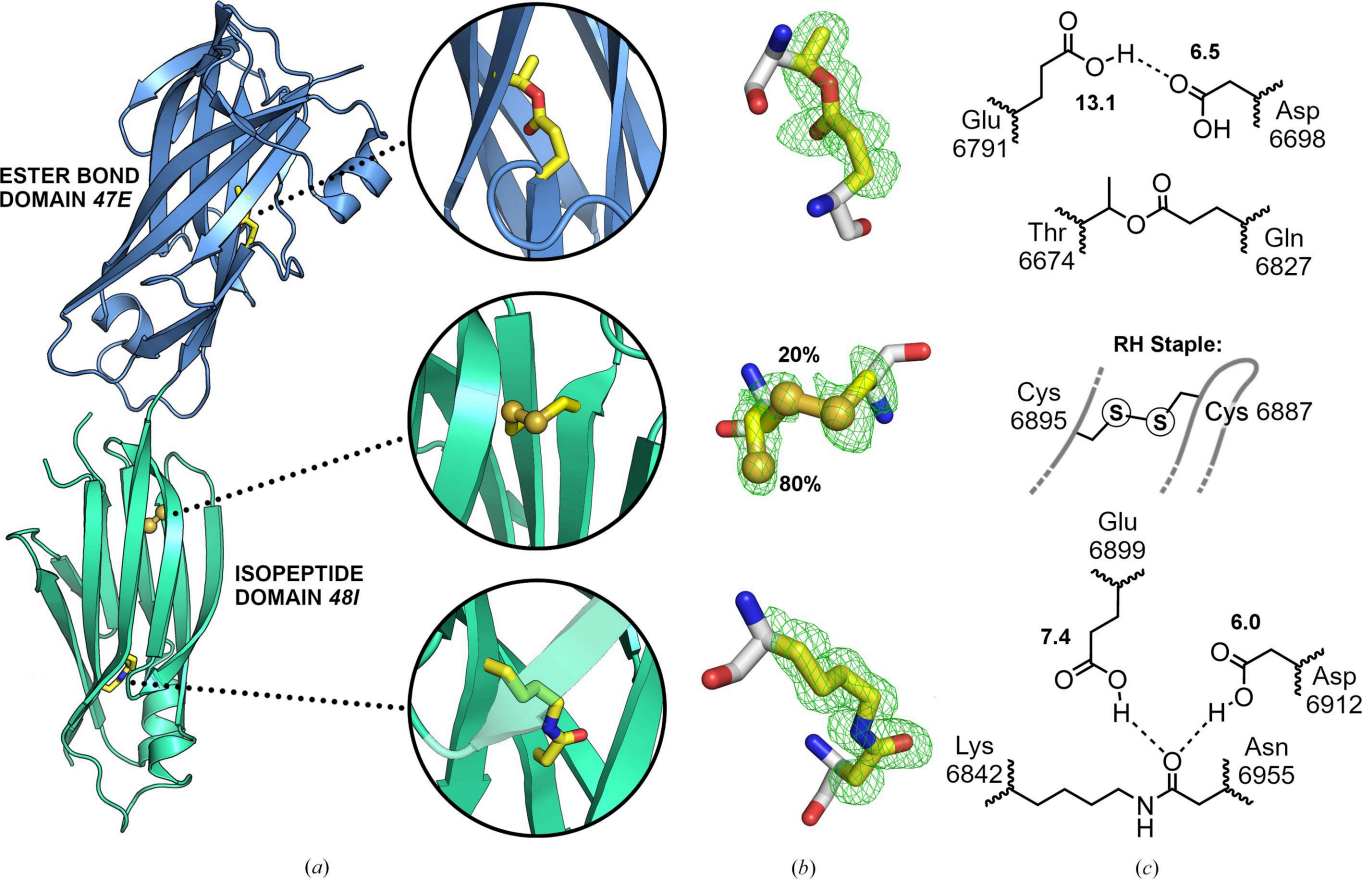
The three different intramolecular cross-links in the *M. mulieris* adhesin domains. (*a*) Overall structure highlighting the locations of the cross-links including enlarged views. (*b*) Electron density for the cross-linked side chains (*F*_o_ − *F*_c_ omit map contoured at 3.0σ). (*c*) *ChemDraw* diagrams showing the bond geometries and hydrogen bonding. p*K*a values for Asp6698, Glu6899 and Asp6912 are indicated.

The environments around each cross-link site are illustrated in Fig. 3 ▸. In contrast to the exemplar Cpe0147 structure (PDB entry 4ni6), the intramolecular ester bond between Thr6674 and Gln6827, although in a near-identical location, shows minor variations in the conformation of adjacent side chains and their interactions in 46E (Supplementary Fig. S7*a*). As in Cpe0147, a hydrogen-bonded pair of buried acidic residues (Glu6791 and Asp6698 in 46E) adjoins the ester bond, where they could contribute to the bond-forming reaction. In 46E, however, they do not directly interact with the resulting ester moiety. These acid residues have high predicted p*K*_a_ values, indicating that both are protonated, again similarly to Cpe0147. All other accessory side chains are appropriately placed for the proposed serine protease-like mechanism of bond formation (Kwon *et al.*, 2014 ▸).

At the N-terminal end of the isopeptide domain 47I, the disulfide bond between Cys6895 and Cys6887 links two adjacent strands within the same β-sheet. Whether this conserved disulfide contributes significantly to protein stability is not clear, although we note that a disulfide bond is found in a similar location in the isopeptide-domain structures of the *Actinomyces oris* fimbrial adhesin FimP and the *A. naeslundii* fimbrial adhesin FimA (PDB entries 3uxf and 3qdh, respectively; Persson *et al.*, 2012 ▸; Mishra *et al.*, 2011 ▸). The conformation of the disulfide bond in the *M. mulieris* structure has a high-energy and potentially reactive −RH Staple conformation that can stabilize β-sheet structures by linking adjacent strands (Schmidt *et al.*, 2006 ▸).

The isopeptide bond in the *M. mulieris* 47I domain is located at the C-terminal end of the domain and has an environment similar to that in the Spy0128 domain 2 structure (PDB entry 3b2m; Supplementary Fig. S7*b*). The hydrophobic environment around Lys6842, consisting of three aromatic side chains and a number of other nonpolar groups, would modify the p*K*_a_ of its ɛ-amino group, enabling cross-link formation to proceed as previously outlined by Kang *et al.* (2007 ▸). In the *M. mulieris* domain, two acidic side chains form hydrogen bonds to the isopeptide bond, implying that their polarizing effect promotes bond formation. As for the ester-bond site, both acidic side chains are predicted to have elevated p*K*_a_ values and are hence mostly protonated.

### 3.5. A putative TED domain in the N-terminal region reinforces a role in adhesion

Finally, we considered the question of whether these proteins are indeed adhesins. Cell-surface adhesins typically comprise an N-terminal adhesion domain supported on a repetitive ‘stalk’ that projects the adhesive apparatus away from the bacterial surface. In our analysis of the *M. mulieris* and *M. curtisii* sequences, the N-terminal region preceding the first recognizable stalk domain is more than 2000 residues long. In searching for possible structured domains in this region, we again attempted to predict the structure using *AlphaFold*2. This revealed a thioester cross-link domain (TED) in both proteins. Secondary-structure and domain elements overlay well between the *M. mulieris*/*M. curtisii* and SaTIE proteins (Miller *et al.*, 2018 ▸). Specifically, a conserved TQ*XX*φW motif, where φ is aromatic, and a predicted thio­ester bond between Cys529 and Gln712 could contribute to host-cell adhesion (Supplementary Fig. S8). No adhesion domain nor any other predicted folded structure could be inferred for the *V. cambriense* protein.

## 4. Discussion

Intramolecular ester, isopeptide, disulfide and thioester bonds, revealed by mass spectrometry and 3D structure prediction and by X-ray crystallography in the numerous and repetitive domains of the *M. mulieris* and *M. curtisii* adhesins, are a clear illustration of the diversity of cross-linking interactions that are now known to be possible (Baker *et al.*, 2015 ▸; Kang & Baker, 2012 ▸; Kang *et al.*, 2007 ▸; Kwon *et al.*, 2014 ▸; Walden *et al.*, 2015 ▸; Miller *et al.*, 2018 ▸). While ester- and isopeptide-bond cross-links demonstrably provide structural stability towards chemical, proteolytic and mechanical stressors, including tensile and shear forces, a thioester bond is unlikely to provide protein stabilization, but would instead effect host-cell adhesion by covalently joining the adhesin protein to the target substrate. Thioester bonds are well characterized in a number of other bacterial cell-surface proteins with N-terminal TED domains and their importance in adhesion has been clearly demonstrated (Walden *et al.*, 2015 ▸; Miller *et al.*, 2018 ▸).

From an evolutionary and protein-stability perspective, each intramolecular cross-link in the *M. mulieris* stalk domain is likely to protect the elongated, single-molecule-wide protein from tensile and shear forces, as well as from proteolytic action, by maintaining compact and rigid domain structures. Remarkable in this case is the extraordinary size of the *M. mulieris* adhesin as a single gene construct compared with polymerized pili such as the *S. pyogenes* Spy0128 adhesin. The retention of such an extended open reading frame suggests that the encoded protein has a vital role in the bacterial life cycle, perhaps in virulence or survival.

The conspicuous domain conservation of these large proteins in the *M. mulieris* and *M. curtisii* proteomes suggests a common ancestry. We presume that the evolutionary growth of these multi-domain proteins occurs via domain duplication through recombination either within the gene or between similar genes in the bacterium, or from external bacterial DNA sources. The major difference between the *M. mulieris* and *M. curtisii* proteins is the insertion of 21 additional isopeptide domains (Fig. 1 ▸). The second largest protein in the *M. mulieris* proteome is also a predicted LP*x*TG-motif cell-wall-anchored protein, for which 3D structure prediction (data not shown) provides a strong prediction of repetitive isopeptide-containing Ig-like domains (Table 5 ▸). This putative 2542-amino-acid isopeptide-rich protein could be the source of the isopeptide domains in the 7651-amino-acid protein. This argument is even more compelling in *M. curtisii*, as the second, third and fourth largest proteins encoded by this organism all appear to be cell-surface adhesins that are likely to contain isopeptide cross-linked domains (Table 5 ▸). We also predict that the largest protein in *V. cambriense*, a third bacterium isolated from bacterial vaginosis clinical samples, also possesses a mixed ester- and isopeptide-domain adhesin. At 3249 amino acids long, this putative adhesin shares 33% coverage and 11% sequence identity with the *M. mulieris* mixed adhesin (Fig. 1 ▸ and Supplementary Fig. S9). The oversized adhesins in all three of these bacteria may be a defining feature of Actinomyces bacteria that inhabit the vaginal mucosa.

What might be the function of these super-sized adhesins in these bacteria? Oversized, single-protein adhesins are found in biofilm-forming bacteria living in extreme environments. Arguably the most extreme example is the bacterium *Marinomonas primoryensis* that survives attached to the underside of ice shelves (Bar Dolev *et al.*, 2016 ▸). This bacterium uses surface adhesins containing several sections of repetitive protein domains to afford both ice adhesion and biofilm formation at the ice–water interface (Guo *et al.*, 2017 ▸). Its giant 1.5 MDa adhesin is thought to mediate biofilm formation through ∼120 calcium-binding Ig-like repeat domains. Calcium binding produces a more rigid and tightly folded Ig-like domain in much the same way as provided by the intramolecular ester and isopeptide cross-links. Given that persistent bacterial vaginosis has been linked to *Mobiluncus* species and biofilm formation (Jung *et al.*, 2017 ▸), it is conceivable that Actinomyces bacteria promote biofilm formation through their super-sized adhesins. While this role remains to be linked to the adhesins from *Mobiluncus* and *Varibaculum*, this can now be investigated as an attractive working hypothesis.

## Supplementary Material

PDB reference: bacterial adhesin from *Mobiluncus mulieris*, 5u5o

PDB reference: 5u6f

Supplementary Tables and Figures. DOI: 10.1107/S2059798323007507/jb5059sup1.pdf

## Figures and Tables

**Table 1:** Macromolecule-production information

Source organism	*Mobiluncus mulieris*
DNA source	*M. mulieris* genomic DNA
Forward primer†	5′-tattttcagggcgccAAGCCTGGAGTGGGCACCTACGCTAC-3′
Reverse primer†	5′-gaattccggatccattcaGTAGCTAAACGAGTTTTCTGCGGTTACTTCGACATTC-3′
Cloning vector	pProEX HTa
Expression vector	pProEX HTa
Expression host	*E. coli* BL21(DE3)
Complete amino-acid sequence of the construct produced	KKPGVGTYATVDKLKAFDVTDGKKDAFTIKDTVRLYNVEEGKTYAIAGQLYEQSVAGDEGSALAKAATTVKVTASMAKPATEVEKTKYGEDVKVYETEMDLTVKREDLTKNQVVKDDIALVVYEQLWAEGTYEKVNDTEVTPKGKSEPVAKHNDPQSSSQSITAEPQFGSLKLTKTVTGWEDAFAKVARPEASYKFTVKCVQKGSVDEFTLKEGEEKTVEGIPLGDTCTISEDVQGAVNQAGLKDTVKFTAVNGVTVDSQVNGEAVVKIGGTANGSDTVANVEVTAENSFSY

† Both primers contain a 5′ 15-base-pair sequence complementary to the pProEX HTa vector to facilitate In-Fusion recombination cloning (indicated in lower case) followed by a part of the gene sequence in upper case.

**Table 2:** Crystallization

Method	Vapour diffusion, hanging drop
Plate type	Linbro 24-well
Temperature (K)	291
Protein concentration (mg ml^−1^)	250
Buffer composition of protein solution	20 m*M* HEPES–NaOH pH 7.0, 100 m*M* NaCl
Composition of reservoir solution	10%(*v*/*v*) PEG 8000, 20%(*v*/*v*) ethylene glycol, 0.02 *M* carboxylic acids (sodium formate, ammonium acetate, trisodium citrate, sodium potassium tartrate, sodium oxamate), 0.1 *M* MES–imidazole pH 6.5
Volume and ratio of drop	1.0 µl, 1:1
Volume of reservoir (µl)	500

**Table 3:** Data collection and processing Values in parentheses are for the outer shell.

	*P*1 structure	*P*2_1_ structure	SeMet
Diffraction source	Beamline MX1, Australian Synchrotron	Beamline MX2, Australian Synchrotron	Beamline MX1, Australian Synchrotron
Wavelength (Å)	0.95370	0.95370	0.95370
Temperature (K)	100	100	100
Detector	ADSC Quantum 210r CCD	ADSC Quantum 315 CCD	ADSC Quantum 210r CCD
Crystal-to-detector distance (mm)	80.06	100.06	120.00
Rotation range per image (°)	1.0	1.0	1.0
Total rotation range (°)	720.0	360.0	360.0
Exposure time per image (s)	1.0	1.0	1.0
Space group	*P*1	*P*2_1_	*P*2_1_
*a*, *b*, *c* (Å)	27.90, 54.98, 57.32	34.55, 51.60, 81.21	34.66, 51.69, 81.21
α, β, γ (°)	67.72, 76.47, 85.35	90.00, 101.60, 90.00	90.00, 101.59, 90.00
Mosaicity (°)	0.28	0.80	0.24
Resolution range (Å)	51.73–1.15 (1.17–1.15)	79.50–1.50 (1.53–1.50)	43.3–1.40 (1.42–1.40)
Total No. of reflections	807789 (38851)	322292 (14567)	409094 (17529)
No. of unique reflections	102468 (4921)	43553 (2081)	55013 (2545)
Completeness (%)	94.1 (90.6)	97.1 (95.5)	99.2 (94.0)
Multiplicity	7.9 (7.9)	7.4 (7.0)	7.4 (6.9)
〈*I*/σ(*I*)〉	20.2 (1.0)	22.3 (2.7)	18.6 (2.0)
*R*_p.i.m._ †	0.026 (0.884)	0.023 (0.447)	0.020 (0.330)
CC_1/2_ ‡	1.000 (0.549)	1.000 (0.919)	1.000 (0.892)
Overall *B* factor from Wilson plot (Å^2^)	10.1	13.9	12.1
DelAnom correlation between half-sets			0.699 (0.071)
Mid-slope of anomalous normal probability			1.081

† Precision-indicating *R* factor (Weiss, 2001 ▸).

‡ Correlation coefficient (Karplus & Diederichs, 2012 ▸).

**Table 4:** Structure solution and refinement Values in parentheses are for the outer shell.

	*P*1 structure	*P*2_1_ structure
Resolution range (Å)	51.73–1.15 (1.18–1.15)	79.55–1.50 (1.539–1.500)
Completeness (%)	94.0	96.8
No. of reflections, working set	97278 (6942)	41295 (2925)
No. of reflections, test set	5177 (375)	2217 (193)
Final *R*_cryst_	0.181 (0.281)	0.234 (0.310)
Final *R*_free_	0.211 (0.286)	0.267 (0.335)
No. of non-H atoms		
Protein	2231	2155
Water	323	124
Total	2554	2279
R.m.s. deviations		
Bond lengths (Å)	0.011	0.007
Angles (°)	1.48	1.29
Average *B* factors (Å^2^)		
Protein	18.7	21.0
Water	30.0	26.5
Ramachandran plot		
Most favoured (%)	98.0	99.0
PDB code	5u5o	5u6f

**Table 5:** Largest gene products in *Mobiluncus* species: putative adhesins Gene products identified from the NCBI Genome Database entries for *M. mulieris* ATCC 35243 and *M. curtisii* ATCC 43063 were assessed by pairwise amino-acid sequence alignments with isopeptide- or ester-bond-containing Ig-like domains and by 3D structure prediction with *AlphaFold*2 (Jumper *et al.*, 2021 ▸; Varadi *et al.*, 2022 ▸). For the 7651-residue *M. mulieris* adhesin, functional prediction was aided by mass spectrometry and crystal structure analysis as described.

*M. curtisii*			*M. mulieris*			
Accession No.	Protein product	No. of amino acids	Accession No.	Protein product	No. of amino acids	Predicted function and/or features
NC_014246.1	WP_013188882.1	5040	NZ_GG668520.1	WP_004013458.1	7651	Adhesin, repeat Ig-like domains displaying isopeptide-, disulfide- and ester-bond cross-links
NC_014246.1	WP_013188810.1	4048	—	—	—	Cadherin-like repeat domains
NC_014246.1	WP_013188829.1	2549	NZ_GG668518.1	WP_004012341.1	2542	Adhesin, repeat Ig-like domains with predicted isopeptide bonds
NC_014246.1	WP_013188586.1	2364	—	—	—	Adhesin, repeat Ig-like domains with predicted isopeptide bonds
