# Supplementary material for: Domain structure and cross-linking in a giant adhesin from the Mobiluncus mulieris bacterium
Source: Acta Crystallogr D Struct Biol. 2023 Oct 20;79(Pt 11):971–9. doi: 10.1107/S2059798323007507 (PMC10619420; doi:10.1107/S2059798323007507)
Supplement: Supplementary file 1 [file d-79-00971-sup1.pdf]

STRUCTURAL BIOLOGY

Volume 79 (2023)

Supporting information for article:

Domain structure and cross-linking in a giant adhesin from the *Mobiluncus mulieris* bacterium

Paul G. Young, Jacob M. Paynter, Julia K. Wardega, Martin J. Middleditch, Leo S. Payne, Edward N. Baker and Christopher J. Squire

Table S1 *M. mulieris* adhesin AlphaFold2 structure predictions for individual domains.

For RMSD calculation, putative isopeptide bond-containing domains were aligned to the major pilin Spy0128 from *Streptococcus pyogenes* (PDB ID 3B2M), and putative ester bond-containing domains were aligned to a repeat domain from *Clostridium perfringens* adhesin Cpe0147 (PDB ID 4NI6). The putative adhesin domain was aligned to the thioester domain of *Enterococcus faecium*, TIE86 (PDB ID 6FWY).

Domain Identifier	Amino acid range	RMSD (Å)	No. Cα aligned	Sequence identity (%)	Predicted structure
n/a	Met1 - Pro472	n/a	n/a	n/a	no structure predicted
A	Ile473 - Ser765	5.33	53/293	9.43	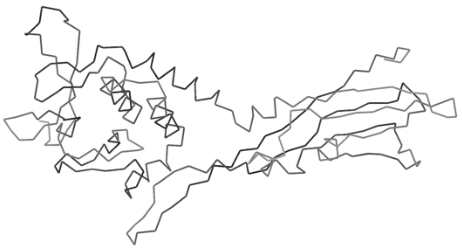 thioester domain†
1	Ser764 - Gly919	2.92	121/156	16.53	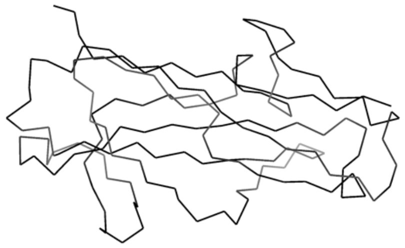 ester
2	Ser920 - Ala1061	3.11	86/142	9.30	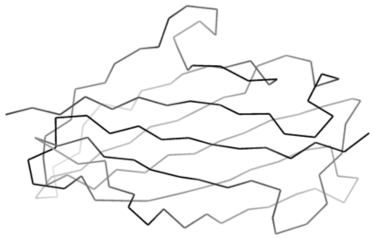 isopeptide
3	Asp1062 - Glu1181	3.08	95/120	16.84	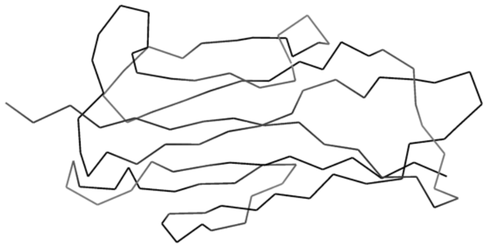 isopeptide

4	Leu1182 - Val1330	3.26	91/149	12.09	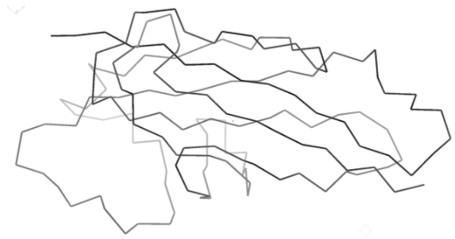 isopeptide
5	Pro1331 - Lys1447	2.09	81/117	16.05	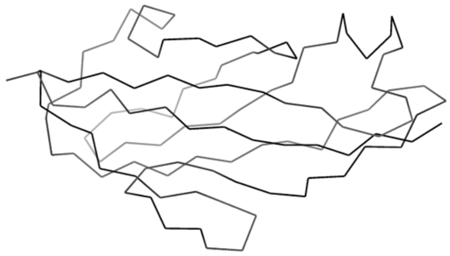 isopeptide
6	Asp1448 - Pro1574	3.16	90/127	23.33	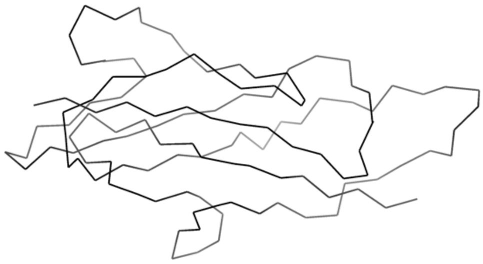 isopeptide
7	Lys1575 - Asp1694	3.13	84/120	11.90	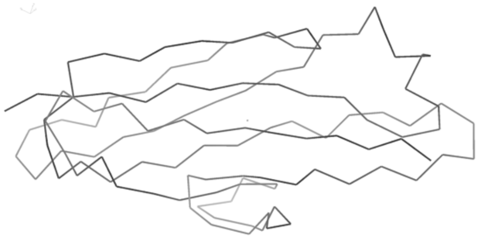 isopeptide
8	Ile1695 - Arg1821	3.68	97/127	6.19	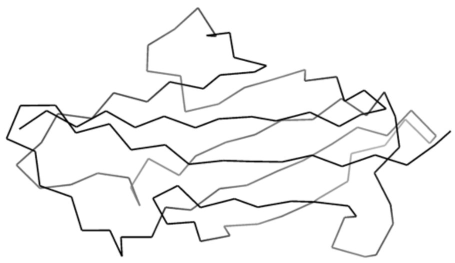 isopeptide

9	Lys1822 - Thr1940	2.70	85/119	8.24	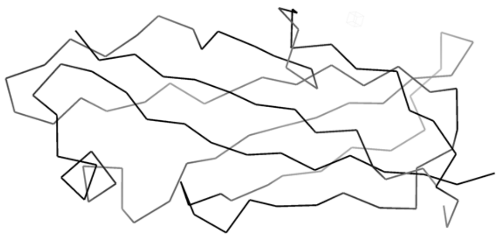 isopeptide
10	Pro1941 - Arg2062	2.12	88/122	14.77	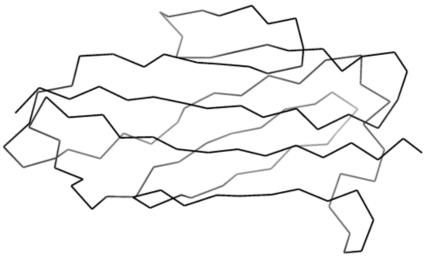 isopeptide
11	Gln2063 - Val2191	2.39	87/129	14.94	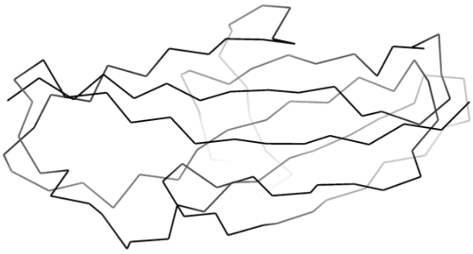 isopeptide
12	Gln2192 - Tyr2308	2.34	87/117	19.54	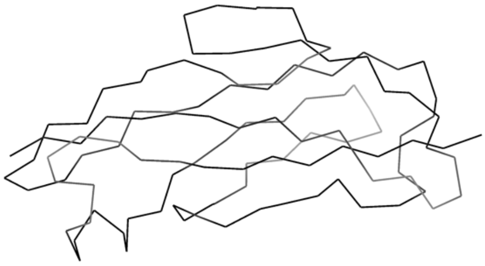 isopeptide
13	Asn2309 - Val2430	3.14	91/122	10.99	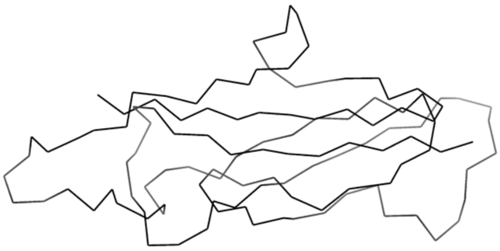 isopeptide

14	Ser2431 - Ile2556	3.10	86/126	4.65	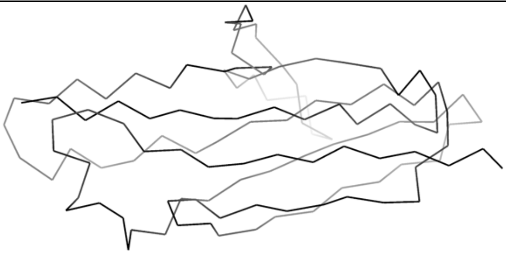 isopeptide
15	Pro2557 - Arg2682	3.59	92/126	9.78	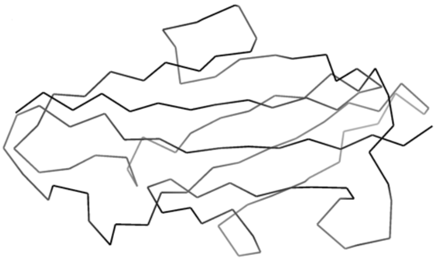 isopeptide
16	Lys2683 - Pro2797	3.15	87/115	5.75	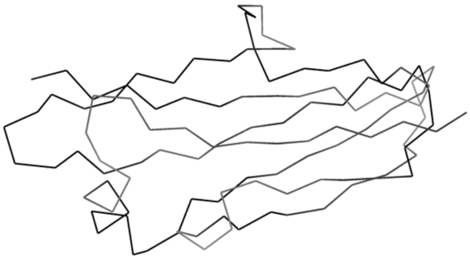 isopeptide
17	Tyr2798 - His2919	2.52	90/122	10.00	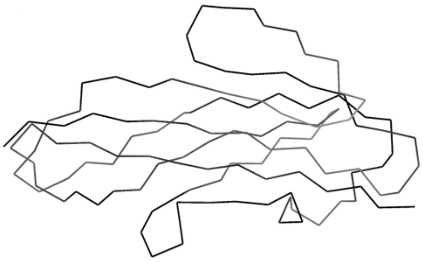 isopeptide
18	Gln2920 - Val3048	2.67	92/129	11.96	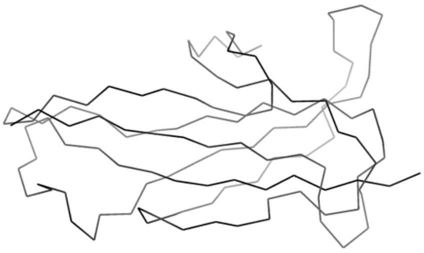 isopeptide

19	His3049 - Thr3164	2.56	88/116	20.45	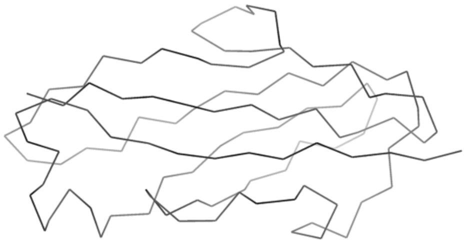<br>isopeptide
20	Tyr3165 - Pro3294	3.25	97/130	27.84	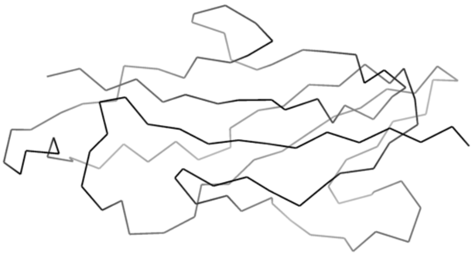<br>isopeptide
21	Gln3295 - Val3422	3.06	90/128	13.33	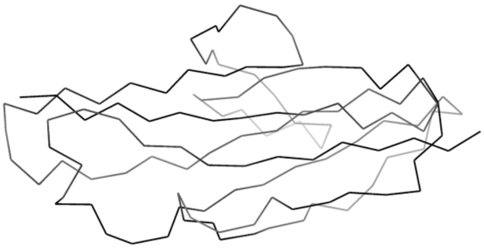<br>isopeptide
22	Ser3423 - Arg3543	3.60	89/121	11.24	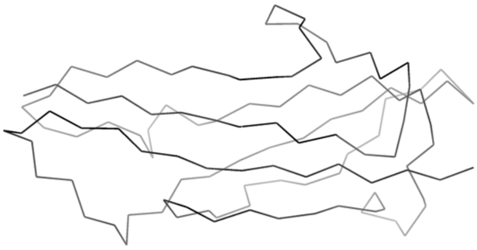<br>isopeptide
23	Glu3544 - Thr3662	2.34	97/119	12.37	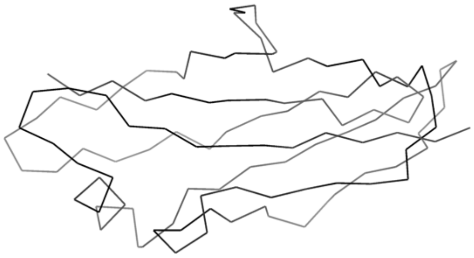<br>isopeptide

24	Pro3663 - Asp3782	2.00	96/120	22.92	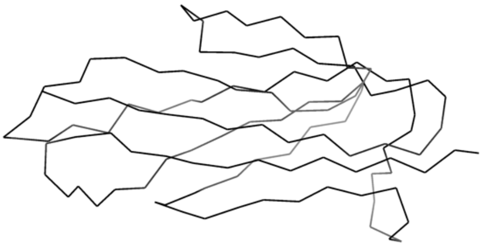 isopeptide
25	Arg3783 - Val3918	4.73	73/136	6.85	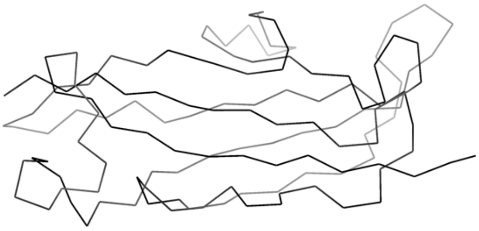 isopeptide
26	Gln3919 - Tyr4032	2.32	86/114	12.79	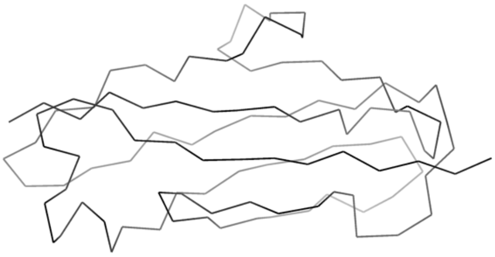 isopeptide
27	Asp4033 - Glu4166	2.78	101/134	27.72	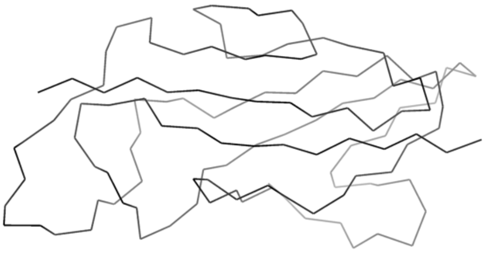 isopeptide
28	Lys4167 - Ile4293	2.91	87/127	9.20	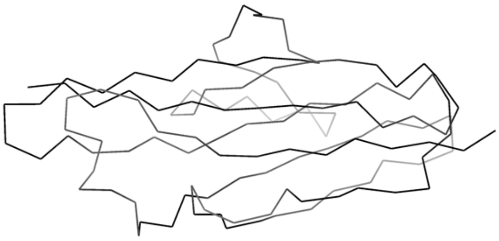 isopeptide

29	Pro4294 - Thr4427	3.62	96/134	10.42	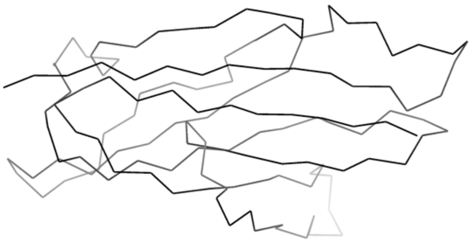 isopeptide
30	Arg4428 - Asp4533	3.91	47/106	4.26	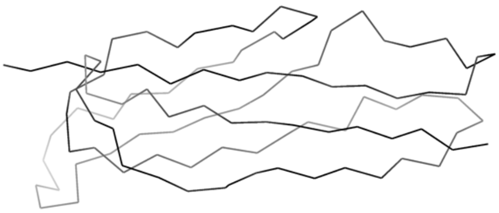 isopeptide
31	Ser4534 - Gln4676	2.49	86/143	11.63	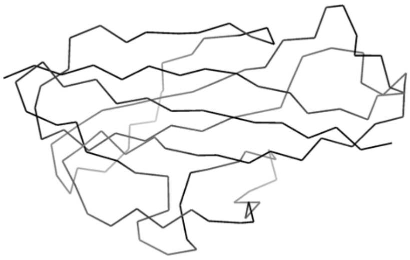 isopeptide
32	Thr4677 - Ser4839	3.34	97/163	5.15	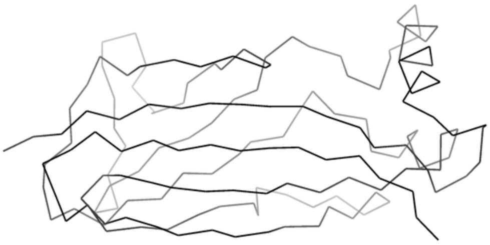 isopeptide
33	Phe4838 - Pro5000	2.42	109/163	13.76	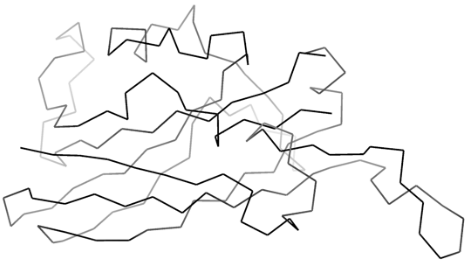 ester

34	Ile5001 - Phe5147	3.84	59/147	16.95	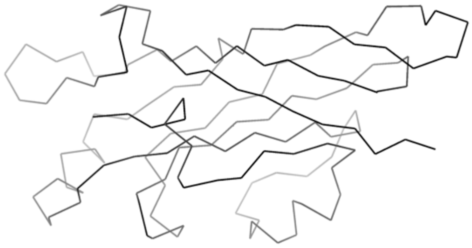 ester
35	Thr5148 - Ser5279	1.51	106/132	31.13	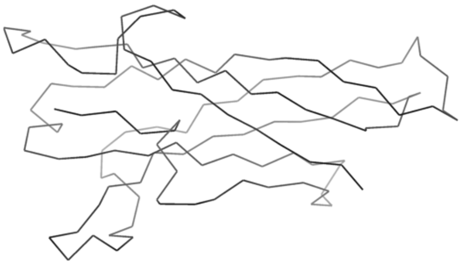 ester
36	Pro5280 - Thr5428	1.58	116/149	31.90	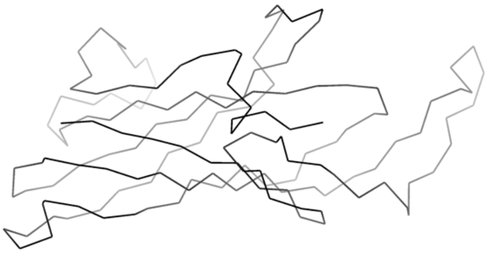 ester
37	Val5429 - Asn5553	1.59	105/125	25.71	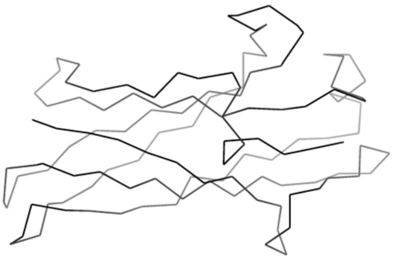 ester
38	Gly5554 - Gln5680	1.70	108/127	27.78	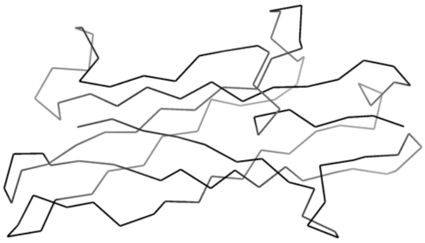 ester

39	Glu5681 - Pro5826	2.11	112/146	25.00	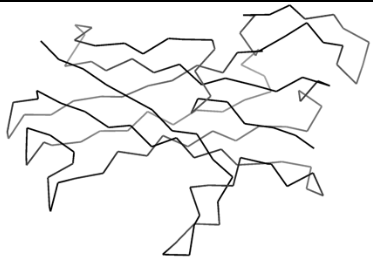 ester
40	Ser5827 - Val5956	1.93	109/130	28.44	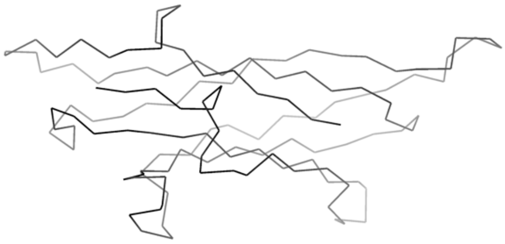 ester
41	Gly5857 - Pro6101	1.56	105/145	28.57	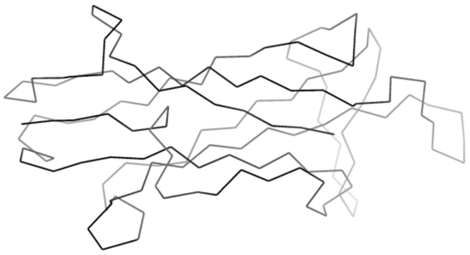 ester
42	Ser6102 - Thr6248	3.28	81/147	25.93	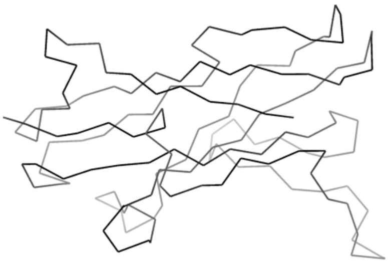 ester
43	Ser6249 - His6384	5.63	44/136	9.09	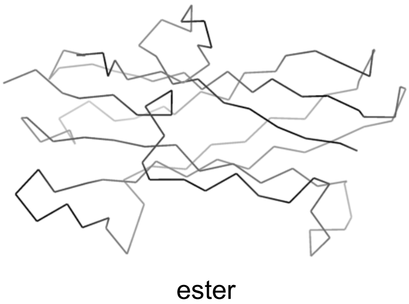 ester

44	Asn6385 - Lys6541	2.00	111/157	22.52	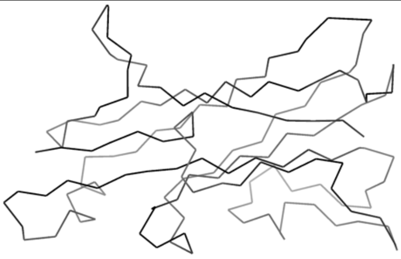 ester
45	Ala6542 - Lys6668	1.88	109/127	30.28	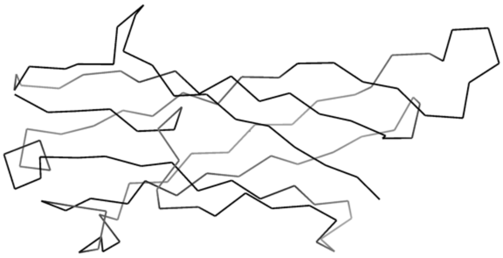 ester
46	Lys6669 - Gln6834	1.73	106/166	20.75	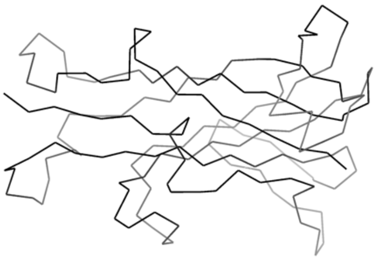 ***46E* ester domain – X-ray crystal structure also determined**
47	Phe6835 - Tyr6959	2.75	82/125	10.98	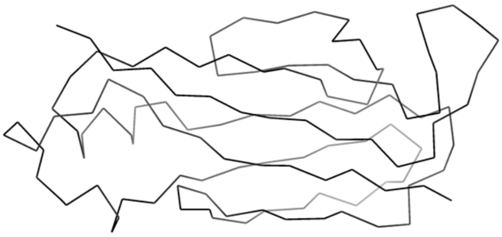 ***47I* isopeptide domain – X-ray crystal structure also determined**
48	Asp6960 - Ser7119	2.39	102/160	23.53	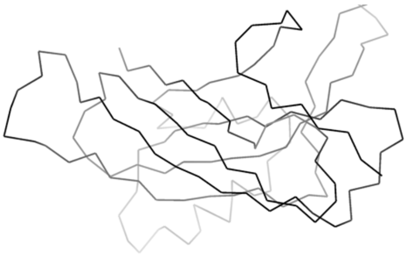 ester

49	Ser7120 - Tyr7263	2.91	93/144	12.90	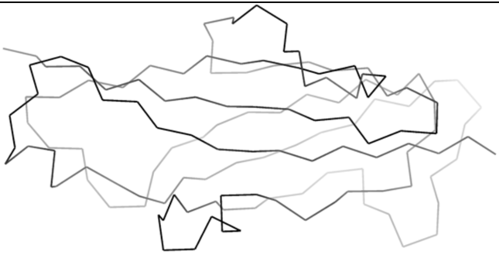 isopeptide
50	Pro7264 - Lys7410	1.44	112/147	35.89	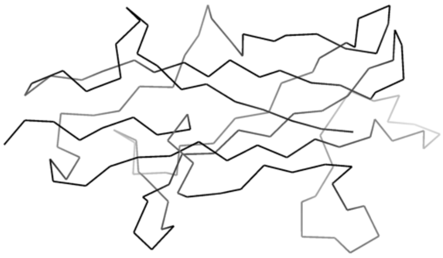 ester
51	Leu7411 - Thr7544	1.49	105/134	32.38	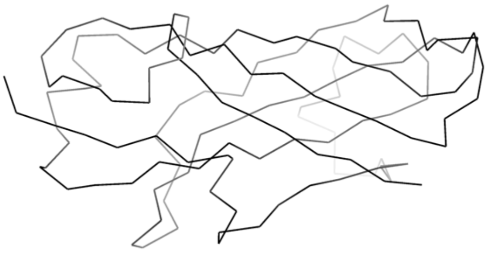 ester

† AlphaFold2 has accurately predicted the expected secondary structure elements of a thioester (TIE) adhesin domain (e.g. PDB ID 6FWY), but has not produced the correct 3D fold.

Table S2 *M. curtisii* adhesin AlphaFold2 structure predictions for individual domains.

For RMSD calculation, putative isopeptide bond-containing domains were aligned to the major pilin Spy0128 from *Streptococcus pyogenes* (PDB ID 3B2M), and putative ester bond-containing domains were aligned to a repeat domain from *Clostridium perfringens* adhesin Cpe0147 (PDB ID 4NI6). The putative adhesin domain was aligned to the thioester domain of *Enterococcus faecium*, TIE86 (PDB ID 6FWY).

Domain Identifier	Amino acid range	RMSD (Å)	No. Cα aligned	Sequence identity (%)	Predicted structure
n/a	Met1 - Val421	n/a	n/a	n/a	no structure predicted
A	Pro422 - Gln724	6.31	48/303	10.42	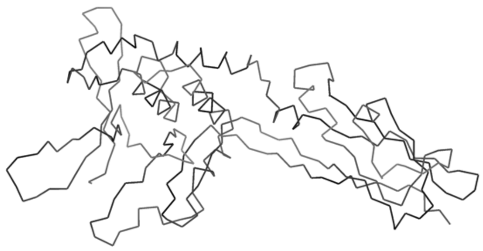 thioester domain†
1	Ile22 - Asn175	2.10	119/154	16.81	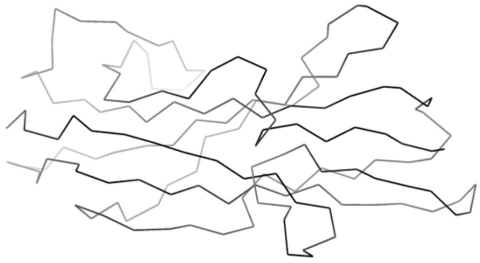 ester
2	Lys176 - Arg304	3.09	78/129	6.41	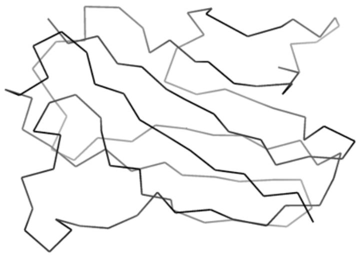 isopeptide
3	Glu305 - Pro340	2.14	93/126	22.58	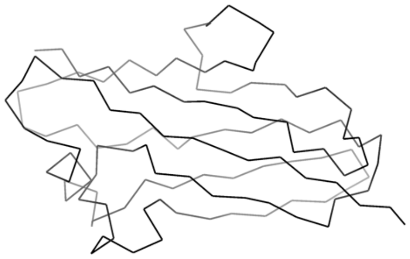 isopeptide

4	Ala341 - Gln548	3.15	85/118	8.24	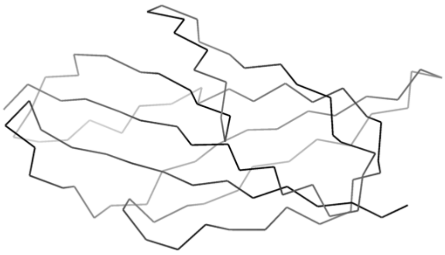<br>isopeptide
5	Lys549 - Ala655	2.99	85/107	20	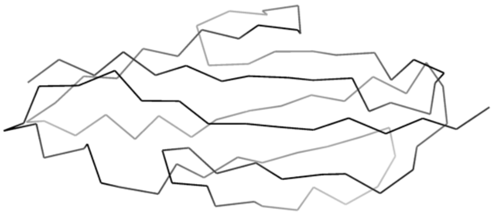<br>isopeptide
6	Asn656 - Tyr771	2.47	81/116	17.28	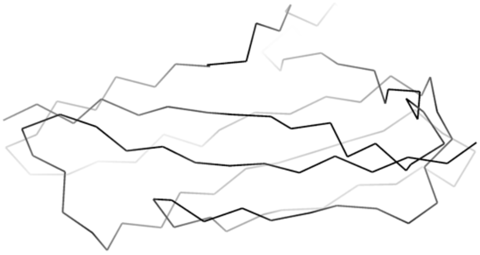<br>isopeptide
7	Asp772 - Glu905	3.57	70/133	4.29	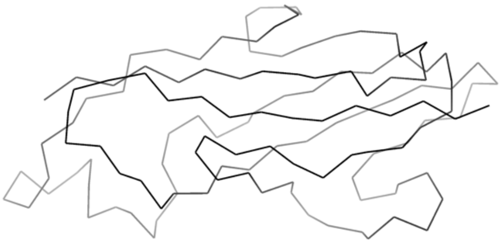<br>isopeptide
8	Lys905 - Val1034	2.93	86/130	4.65	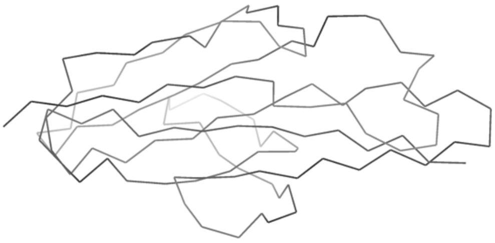<br>isopeptide

9	Pro1035 - Glu1158	3.52	98/124	9.18	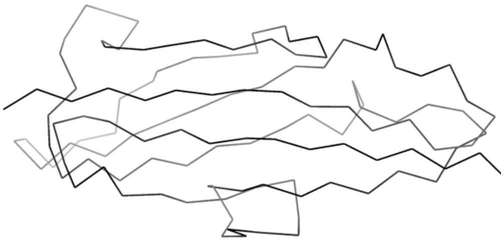 isopeptide
10	Leu1159 - Lys1264	4.51	59/106	6.78	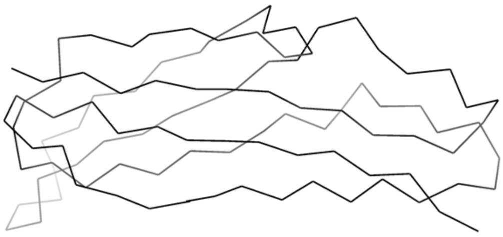 isopeptide
11	Ala1265 - Tyr1406	2.86	88/142	9.09	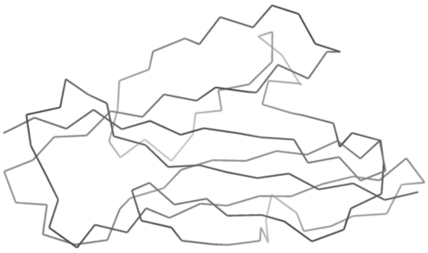 isopeptide
12	Glu1407 - Cys1566	5.02	87/160	5.75	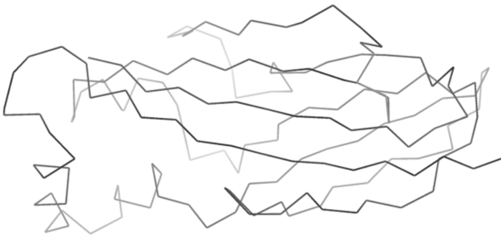 isopeptide
13	Phe1567 - Gln1731	1.99	105/165	15.24	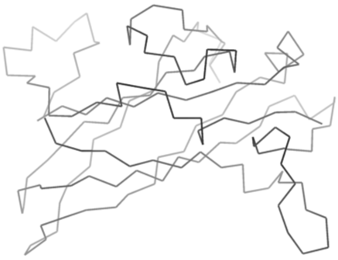 ester

14	Trp1732 - Tyr1877	1.64	102/146	22.55	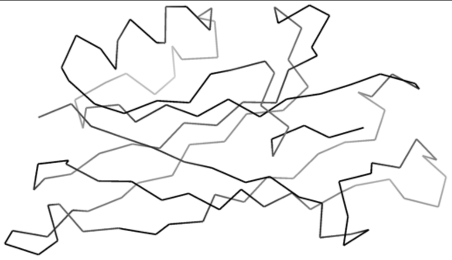 ester
15	Thr1878 - Tyr2003	3.70	58/126	12.07	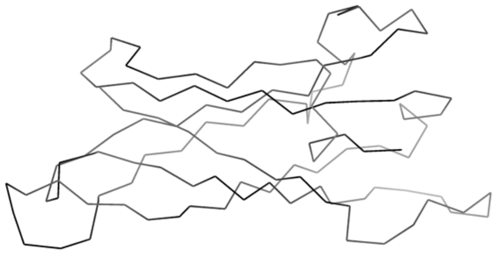 ester
16	Asn2004 - Pro2161	1.82	115/158	33.91	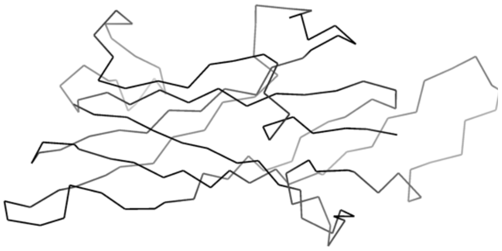 ester
17	Gly2161 - His2296	1.47	103/135	29.13	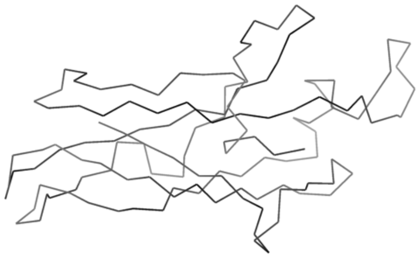 ester
18	Ser2297 - Asn2433	1.93	108/137	25.00	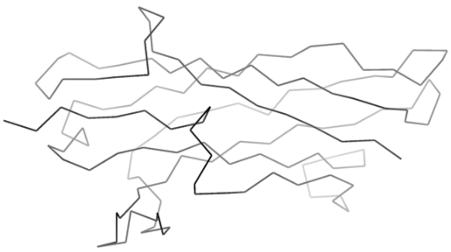 ester

19	Ser2434 - Pro2578	2.09	111/145	24.32	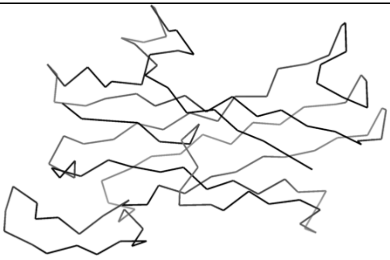 ester
20	Ser2579 - Arg2695	1.56	103/117	26.21	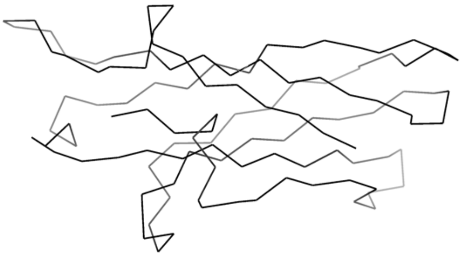 ester
21	Val2696 - Trp2840	1.58	104/145	27.88	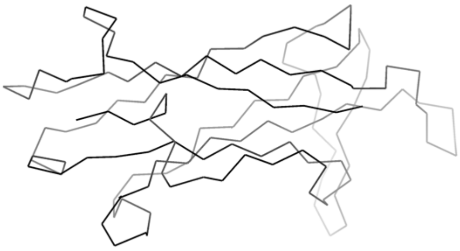 ester
22	Ser2841 - Val2966	1.85	103/126	25.24	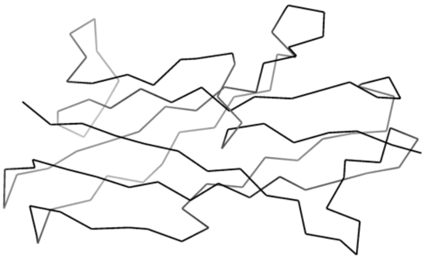 ester
23	Lys2967 - Asp3096	1.67	114/130	25.44	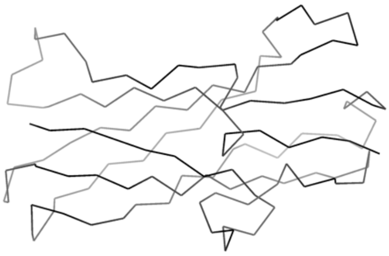 ester

24	Tyr3097 - Asp3244	1.43	107/148	22.43	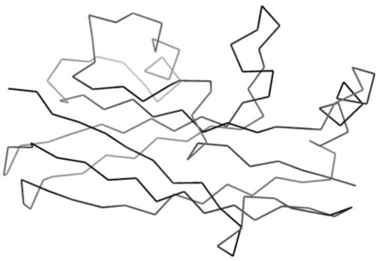 ester
25	Val3245 - Lys3378	2.03	112/134	25.89	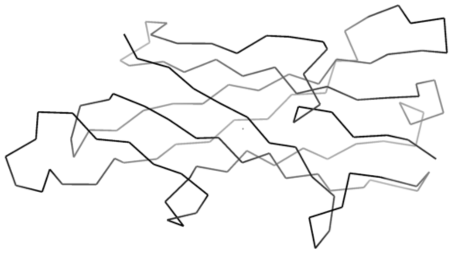 ester
26	His3379 - Pro3548	1.77	106/170	21.70	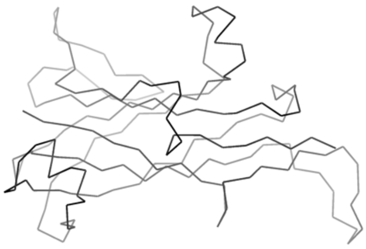 ester
27	Glu3549Asn3674	2.88	84/126	13.10	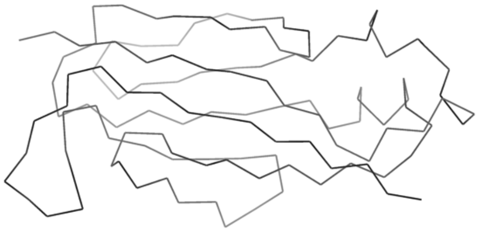 isopeptide
28	Glu3765 - Thr3824	2.29	103/150	25.24	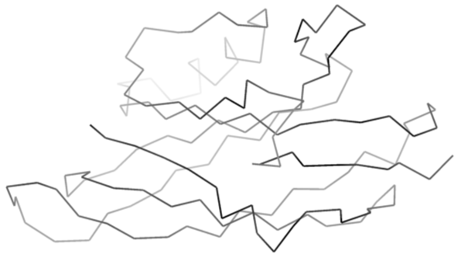 ester

29	His3825 - Val3959	3.24	95/135	11.58	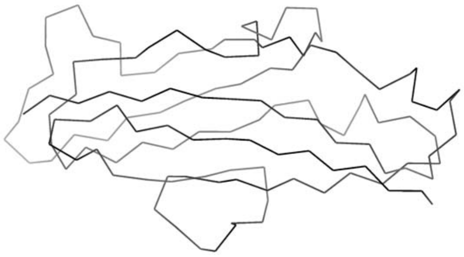 isopeptide
30	Lys3960 - Arg4102	1.93	111/143	25.23	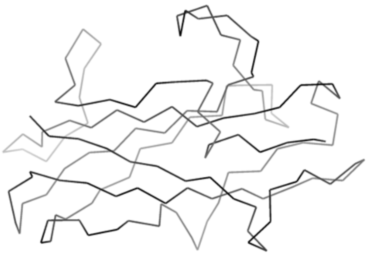 ester
31	Pro4103 - Pro4239	1.26	99/138	36.36	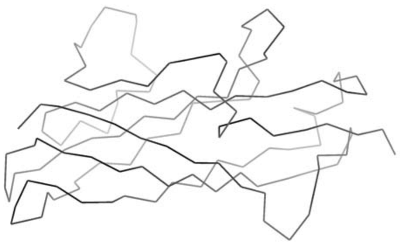 ester

† AlphaFold2 has accurately predicted the expected secondary structure elements of a thioester (TIE) adhesin domain (e.g. PDB ID 6FWY), but has not produced the correct 3D fold.

Table S3 *V. cambrienseu* adhesin AlphaFold2 structure predictions for individual domains.

For RMSD calculation, putative isopeptide bond-containing domains were aligned to the major pilin Spy0128 from *Streptococcus pyogenes* (PDB ID 3B2M), and putative ester bond-containing domains were aligned to a repeat domain from *Clostridium perfringens* adhesin Cpe0147 (PDB ID 4NI6). The putative adhesin domain was aligned to the thioester domain of *Enterococcus faecium*, TIE86 (PDB ID 6FWY).

Domain Identifier	Amino acid range	RMSD (Å)	No. Cα aligned	Sequence identity (%)	Predicted structure
1	Leu19 - Thr513	4.57	49/511	8.16	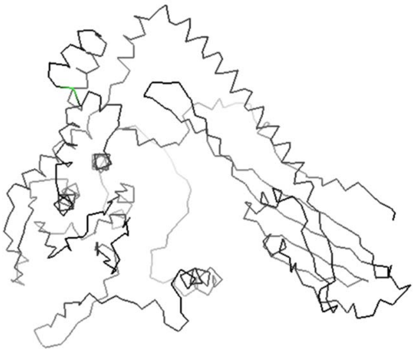 thioester[†]
2	Ala515 - Thr652	2.12	111/138	21.62	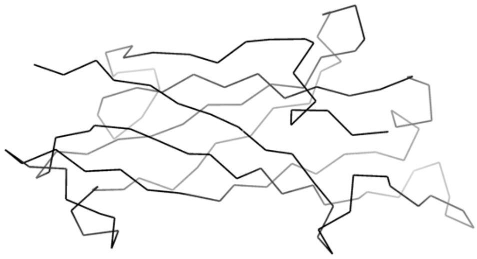 ester
3	Glu653 - Lys788	1.55	107/136	30.84	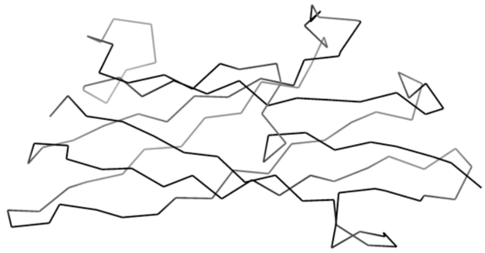 ester
4	Lys791 - Pro921	1.59	105/131	27.62	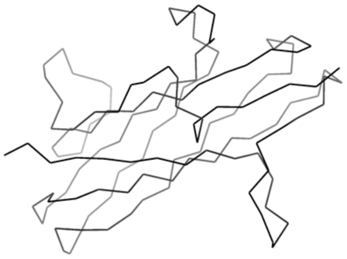 ester

5	Pro924 - Pro1037	2.15	87/114	11.49	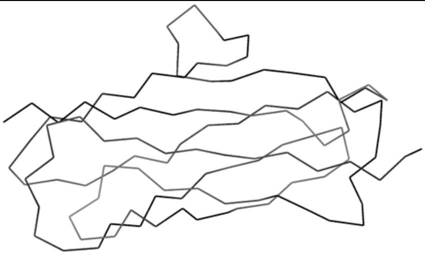
6	Gln1039 - Glu1146	3.14	86/108	6.98	isopeptide 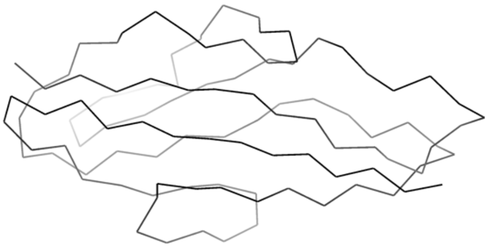
7	Thr1148 - Asn1274	2.95	91/127	14.29	isopeptide 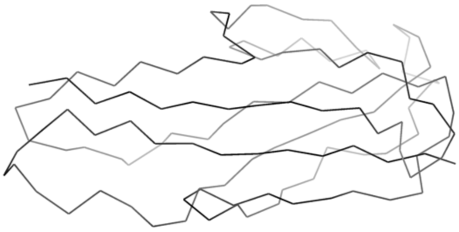
8	Lys1276 - Gln1407	1.84	97/132	23.71	isopeptide 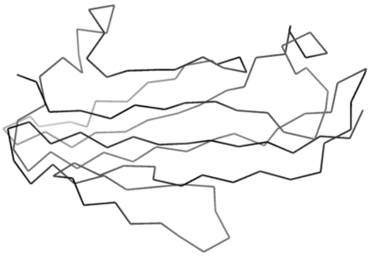
9	Leu1409 - Ile1545	3.35	77/137	7.79	isopeptide 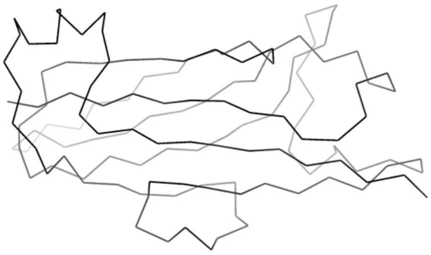
10	Lys1548 - Asp1671	2.89	100/124	17.00	isopeptide 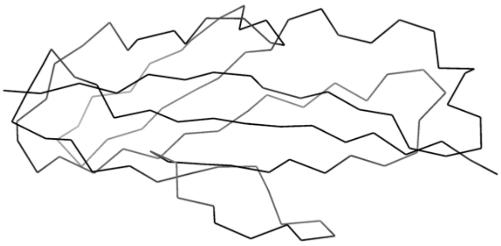

11	Pro1670 - Pro1802	1.20<br>1.62	108/133	25.93	isopeptide<br>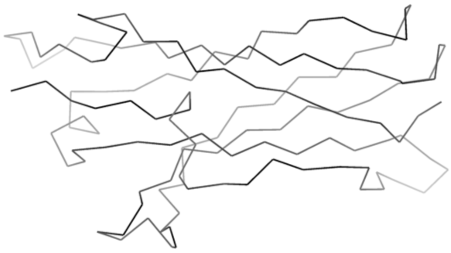
12	Asn1801 - Gly1935	1.94	110/135	28.18	ester<br>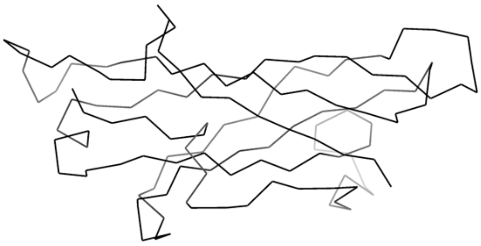
13	Pro1936 - 2069	1.53<br>2.06	108/134	27.77	ester<br>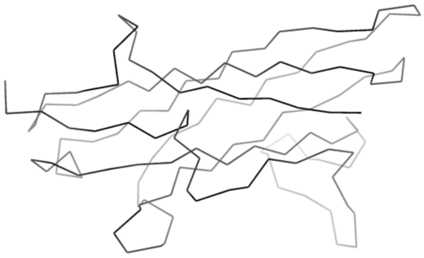
14	Ser2072 - Pro2196	3.25	87/125	10.34	ester<br>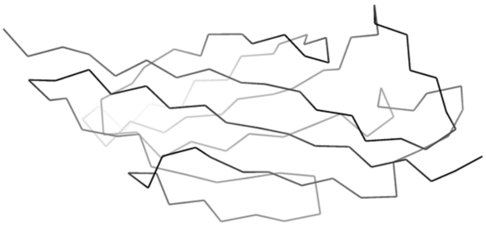
15	Glu2197 - Thr2340	3.40<br>2.02	106/144	25.47	isopeptide<br>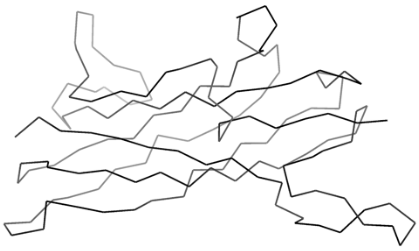<br>ester

16	Pro2344 - Met2469	3.28	97/126	11.34	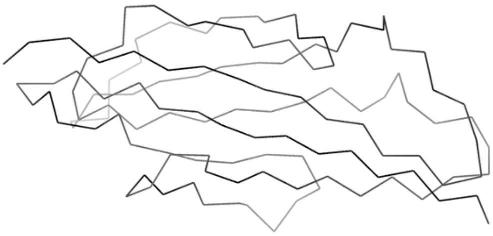
17	Gly2470 - Glu2606	2.05	111/137	34.23	isopeptide 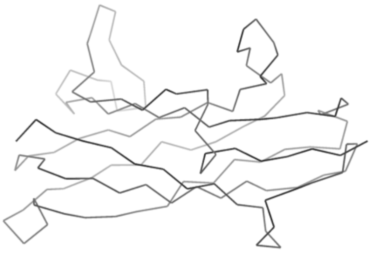
18	Ala2607 - Glu2742	2.72<br>2.22	104/136	27.88	ester 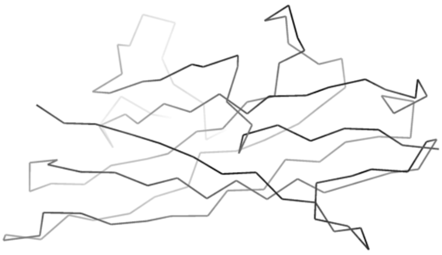
19	Lys2745 - Lys2873	2.849	90/129	14.44	ester 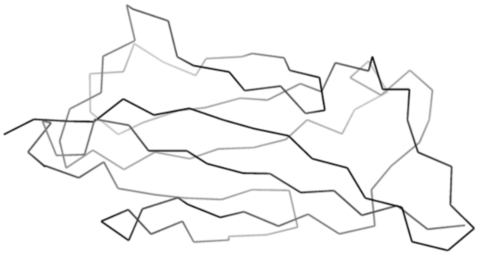
20	Asp2874 - Ala3013	3.62<br>1.67	108/140	25.00	isopeptide 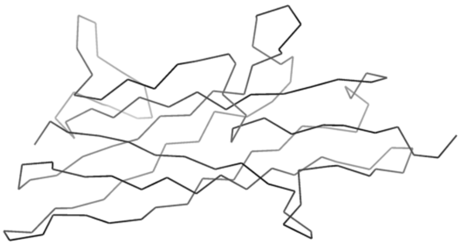
21	Thr3019 - Pro3174	2.16<br>6.46	49/156	6.12	ester 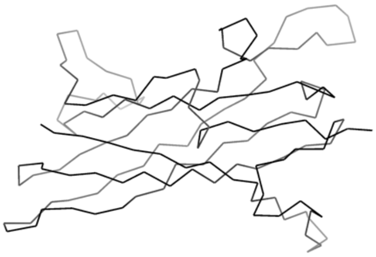

	ester

† AlphaFold2 has accurately predicted the expected secondary structure elements of a thioester (TIE) adhesin domain (e.g. PDB ID 6FWY), but has not produced the correct 3D fold.

```
Reference sequence (1): WP_004013458.1
Identities normalised by aligned length.
Colored by: identity

                    cov    pid     1 [        .         .         .         .         :         .         .         . 80
1 WP_004013458.1 100.0% 100.0%      MGDKTRGTQGRMPVSGLIKVGKGKRRRTTRNWGAAFVAASVAVAWTVGGLVMPSMAAEPAVIGKVHPEVNISGGGTANVS
2 WP_013188882.1  62.4%  35.2%      --------------------------------------------------------------------------------
  consensus/100%                    ................................................................................
  consensus/90%                     ................................................................................
  consensus/80%                     ................................................................................
  consensus/70%                     ................................................................................

                    cov    pid    81          .         1         .         .         .         .         :         . 160
1 WP_004013458.1 100.0% 100.0%      LAGQNTNLYYAALSTNLFTTIDRVYAQKMYGKTEQLGDFTAQYLPRACFVRDDRIISNEPLGNFIRMDEGGPSYLGRRYG
2 WP_013188882.1  62.4%  35.2%      --------------------------------------------------------------------------------
  consensus/100%                    ................................................................................
  consensus/90%                     ................................................................................
  consensus/80%                     ................................................................................
  consensus/70%                     ................................................................................

                    cov    pid   161          .         .         .         2         .         .         .         . 240
1 WP_004013458.1 100.0% 100.0%      AWVLYPNGSKSFSVDSSKSRSADRSVDSPSDVDDADDTEDTLKLTLDKLSFDDVVGDAAAAADKEFSDSQLPNEKTGKEQ
2 WP_013188882.1  62.4%  35.2%      --------------------------------------------------------------------------------
  consensus/100%                    ................................................................................
  consensus/90%                     ................................................................................
  consensus/80%                     ................................................................................
  consensus/70%                     ................................................................................

                    cov    pid   241          :         .         .         .         .         3         .         . 320
1 WP_004013458.1 100.0% 100.0%      KQTAESPVENPKESLASKEGGETQAKDVPPNGSESSSNSESAPELAKPTPAETSTAETEPKETKKPSETKPSSQFVEEKG
2 WP_013188882.1  62.4%  35.2%      --------------------------------------------------------------------------------
  consensus/100%                    ................................................................................
  consensus/90%                     ................................................................................
  consensus/80%                     ................................................................................
  consensus/70%                     ................................................................................

                    cov    pid   321          .         .         :         .         .         .         .         4 400
1 WP_004013458.1 100.0% 100.0%      HKAGNLDKESKSLEDRSAVSKQGKDSAENSNKADKNQGKDRSAVGQDRAVLFSAFQLKTKELKVKFDPQKSKDKAIEDVN
2 WP_013188882.1  62.4%  35.2%      --------------------------------------------------------------------------------
  consensus/100%                    ................................................................................
  consensus/90%                     ................................................................................
  consensus/80%                     ................................................................................
  consensus/70%                     ................................................................................

                    cov    pid   401          .         .         .         .         :         .         .         . 480
1 WP_004013458.1 100.0% 100.0%      KQVDEIQKDALQQLDEFVTKNRVPQDIADKFRTKLNIQFENMRRQAVEFLEKKYAKGAIGADEWRNCQQSGPIVPDGSLV
2 WP_013188882.1  62.4%  35.2%      --------------------------------------------------------------------------------
  consensus/100%                    ................................................................................
  consensus/90%                     ................................................................................
  consensus/80%                     ................................................................................
  consensus/70%                     ................................................................................

                    cov    pid   481          .         5         .         .         .         .         :         . 560
1 WP_004013458.1 100.0% 100.0%      LVNEARLDNHNKKAWYLGVKQNDYTISRLLQYASEGSGNIKADGADSFCVEPLKGLQPGAHNIYTHPSTLEWAQNHPERA
2 WP_013188882.1  62.4%  35.2%      --------------------------------------------------------------------------------
  consensus/100%                    ................................................................................
  consensus/90%                     ................................................................................
  consensus/80%                     ................................................................................
  consensus/70%                     ................................................................................

                    cov    pid   561          .         .         .         6         .         .         .         . 640
1 WP_004013458.1 100.0% 100.0%      ELVKALAWHYKQFAQPKEYAKYQNAIWSVVMGLPSMVGSWDGMKRFWVSEPGSSAGLISAAESAYKTRPSVVQALKSMHL
2 WP_013188882.1  62.4%  35.2%      --------------------------------------------------------------------------------
  consensus/100%                    ................................................................................
  consensus/90%                     ................................................................................
  consensus/80%                     ................................................................................
  consensus/70%                     ................................................................................

                    cov    pid   641          :         .         .         .         .         7         .         . 720
1 WP_004013458.1 100.0% 100.0%      DQISVDTAPDGKSKVIFLQLNDYDKSSEIRRAIGNSVYLRVTGATTPEGKPVDRVSLEEAERGVHFLVKNQDQFSVAFAG
2 WP_013188882.1  62.4%  35.2%      --------------------------------------------------------------------------------
  consensus/100%                    ................................................................................
  consensus/90%                     ................................................................................
  consensus/80%                     ................................................................................
  consensus/70%                     ................................................................................

                    cov    pid   721          .         .         :         .         .         .         .         8 800
1 WP_004013458.1 100.0% 100.0%      TLNNAQDAYFLDKPSHDTQAQVTVLSKKITVSGSLKIQWGTTASSDPQVGTTVLVNGNNQNPGGPETVDISKYGQNDTIT
2 WP_013188882.1  62.4%  35.2%      --------------------------------------------------------------------------------
  consensus/100%                    ................................................................................
  consensus/90%                     ................................................................................
  consensus/80%                     ................................................................................
  consensus/70%                     ................................................................................
```

```
                       cov     pid  801          .         .         .         .         :         .         .         . 880
  1 WP_004013458.1 100.0% 100.0%        LTDSVTYSGLAKFAISKDNYYLVGKLKRVRDGKTIEMPVRPMLQDIKDIPAGSFQGIFPVQLKVSDMKAGDKYYYEEYIL
  2 WP_013188882.1  62.4%  35.2%        --------------------------------------------------------------------------------
    consensus/100%                      ................................................................................
    consensus/90%                       ................................................................................
    consensus/80%                       ................................................................................
    consensus/70%                       ................................................................................

                       cov     pid  881          .         9         .         .         .         .         :         . 960
  1 WP_004013458.1 100.0% 100.0%        TTDPRYEKNKKVESGWYGPLSVVVSHEGENPDQTVKVKGSNAKLLINKIGAGESAKAKGNVTFDVYCKKDNKPTTMSRLY
  2 WP_013188882.1  62.4%  35.2%        --------------------------------------------------------------------------------
    consensus/100%                      ................................................................................
    consensus/90%                       ................................................................................
    consensus/80%                       ................................................................................
    consensus/70%                       ................................................................................

                       cov     pid  961          .         .         .         0         .         .         .         . 1040
  1 WP_004013458.1 100.0% 100.0%        APAAGGPASTNGAEPATELVVVPGSNCQVTETYSSQDVTFTDKDAVSFKAVVLGNHQQQQIPAPASTTVDQDGHRLRAVT
  2 WP_013188882.1  62.4%  35.2%        --------------------------------------------------------------------------------
    consensus/100%                      ................................................................................
    consensus/90%                       ................................................................................
    consensus/80%                       ................................................................................
    consensus/70%                       ................................................................................

                       cov     pid 1041          :         .         .         .         .         1         .         . 1120
  1 WP_004013458.1 100.0% 100.0%        SFDVPQGATQVTVNTTNNYQADNAAKFILKKEFRTTDGSTLGDGGYRFTVDCAGYPSDALTLKRENGYQWEFTQLKGKAL
  2 WP_013188882.1  62.4%  35.2%        --------------------------------------------------------------------------------
    consensus/100%                      ................................................................................
    consensus/90%                       ................................................................................
    consensus/80%                       ................................................................................
    consensus/70%                       ................................................................................

                       cov     pid 1121          .         .         :         .         .         .         .         2 1200
  1 WP_004013458.1 100.0% 100.0%        KAGTKCTVKEVSQHQHPNHDWQSVSFSVQGTTGMPEDNGVSFVLPSASQATAVVTATNTYELKKGSLQVTKRVSTNAKDN
  2 WP_013188882.1  62.4%  35.2%        --------------------------------------------------------------------------------
    consensus/100%                      ................................................................................
    consensus/90%                       ................................................................................
    consensus/80%                       ................................................................................
    consensus/70%                       ................................................................................

                       cov     pid 1201          .         .         .         .         :         .         .         . 1280
  1 WP_004013458.1 100.0% 100.0%        PWKNQNFSFEADCFVPHPGHNSWAGQTNFERRRTGAEAASAAWIRPVNVKPGTPTLLANKTLPVGTMCLVWENEVGGDVR
  2 WP_013188882.1  62.4%  35.2%        --------------------------------------------------------------------------------
    consensus/100%                      ................................................................................
    consensus/90%                       ................................................................................
    consensus/80%                       ................................................................................
    consensus/70%                       ................................................................................

                       cov     pid 1281          .         3         .         .         .         .         :         . 1360
  1 WP_004013458.1 100.0% 100.0%        PNLTWTGAGDNVTYTDGASRAHTTARRVFITADNGGTPGIALTATNDFQVPYGSFKISKKVTGDAAGDAKVKNHEFTFKI
  2 WP_013188882.1  62.4%  35.2%        --------------------------------------------------------------------------------
    consensus/100%                      ................................................................................
    consensus/90%                       ................................................................................
    consensus/80%                       ................................................................................
    consensus/70%                       ................................................................................

                       cov     pid 1361          .         .         .         4         .         .         .         . 1440
  1 WP_004013458.1 100.0% 100.0%        NCVGLNEQTVKVRPDQAVDFTTYTKQKVKAGTQCRIHEDAADIKGITSQLTAWEGNNSVQEGNDLKITVPAAGEPELTVS
  2 WP_013188882.1  62.4%  35.2%        --------------------------------------------------------------------------------
    consensus/100%                      ................................................................................
    consensus/90%                       ................................................................................
    consensus/80%                       ................................................................................
    consensus/70%                       ................................................................................

                       cov     pid 1441          :         .         .         .         .         5         .         . 1520
  1 WP_004013458.1 100.0% 100.0%        ATNTVTKDTAKFKIQKLVQPQGTEFNGNFKFTYSCTDPDKNVYTNTKPYGAQAVKDFIEVTAGQSSEEITVPANSKCHVT
  2 WP_013188882.1  62.4%  35.2%        --------------------------------------------------------------------------------
    consensus/100%                      ................................................................................
    consensus/90%                       ................................................................................
    consensus/80%                       ................................................................................
    consensus/70%                       ................................................................................

                       cov     pid 1521          .         .         :         .         .         .         .         6 1600
  1 WP_004013458.1 100.0% 100.0%        EDKSLPSVKDTTSKNPLKYNKLSFNPAGQNQTSGEFSPSKNQVLTVSATNHYTPKTTGFQLSKKVTGNNKANHASDTFNF
  2 WP_013188882.1  62.4%  35.2%        --------------------------------------------------------------------------------
    consensus/100%                      ................................................................................
    consensus/90%                       ................................................................................
    consensus/80%                       ................................................................................
    consensus/70%                       ................................................................................

                       cov     pid 1601          .         .         .         .         :         .         .         . 1680
  1 WP_004013458.1 100.0% 100.0%        GWSCQPREGKAIDGNVTLADNGSHTVSDLPVGTKCVLWENVATPKANENNQTKWTLPGQQAVTGKTINNHANAVEFTVDK
  2 WP_013188882.1  62.4%  35.2%        --------------------------------------------------------------------------------
    consensus/100%                      ................................................................................
```

```
    consensus/90%                ................................................................................
    consensus/80%                ................................................................................
    consensus/70%                ................................................................................

                    cov    pid  1681         .         7         .         .         .         .         :         . 1760
  1 WP_004013458.1 100.0% 100.0%     ENAAIVVEANNSFDIPNTSLTVQKKVVKGDQAVVDNNKEYRVSVSCVYPTDKTNHVVATDKALKADGILKFEADENGIKI
  2 WP_013188882.1  62.4%  35.2%     --------------------------------------------------------------------------------
    consensus/100%               ................................................................................
    consensus/90%                ................................................................................
    consensus/80%                ................................................................................
    consensus/70%                ................................................................................

                    cov    pid  1761         .         .         .         8         .         .         .         . 1840
  1 WP_004013458.1 100.0% 100.0%     PVGASCTVEENRTSAELEGHNLAIQLKVGGKNIADSNANKAENITVPAEGKTVTVENTYNRKLGGFKLTKVVTGEGLDTA
  2 WP_013188882.1  62.4%  35.2%     --------------------------------------------------------------------------------
    consensus/100%               ................................................................................
    consensus/90%                ................................................................................
    consensus/80%                ................................................................................
    consensus/70%                ................................................................................

                    cov    pid  1841         :         .         .         .         .         9         .         . 1920
  1 WP_004013458.1 100.0% 100.0%     EALQANGGKFVFTYDCALGGKSVKTGTLEVTPQTGAEANNIPAGATCTLTEKAVTAPSGFRAPERPTWKVNGADASGVIK
  2 WP_013188882.1  62.4%  35.2%     --------------------------------------------------------------------------------
    consensus/100%               ................................................................................
    consensus/90%                ................................................................................
    consensus/80%                ................................................................................
    consensus/70%                ................................................................................

                    cov    pid  1921         .         .         :         .         .         .         .         0 2000
  1 WP_004013458.1 100.0% 100.0%     IVEKTDATQNPVLEATNTYTPYQVKFNLRKIVNTDDHTQPGGEYGFHVDCGQGATKDVKLNQDNKYTWDSASTPEFSRLS
  2 WP_013188882.1  62.4%  35.2%     --------------------------------------------------------------------------------
    consensus/100%               ................................................................................
    consensus/90%                ................................................................................
    consensus/80%                ................................................................................
    consensus/70%                ................................................................................

                    cov    pid  2001         .         .         .         .         :         .         .         . 2080
  1 WP_004013458.1 100.0% 100.0%     AKSCTVTETVLPTLEGYTYQGVKYSVSDASAATDAAKRSVTFAFPPKGDVSVAVSATNSYRRQQGSIEVEKIVTIADNAK
  2 WP_013188882.1  62.4%  35.2%     --------------------------------------------------------------------------------
    consensus/100%               ................................................................................
    consensus/90%                ................................................................................
    consensus/80%                ................................................................................
    consensus/70%                ................................................................................

                    cov    pid  2081         .         1         .         .         .         .         :         . 2160
  1 WP_004013458.1 100.0% 100.0%     NPWDGKRYSFEADCANPVMNWSDHDRVEVEAGKKGQMHRGRWVGSVCHVWENTADTVDVINQLNWENGCEAVEPYTDKQG
  2 WP_013188882.1  62.4%  35.2%     --------------------------------------------------------------------------------
    consensus/100%               ................................................................................
    consensus/90%                ................................................................................
    consensus/80%                ................................................................................
    consensus/70%                ................................................................................

                    cov    pid  2161         .         .         .         2         .         .         .         . 2240
  1 WP_004013458.1 100.0% 100.0%     VEHPQACKVTIKADSKGTSGVKVSAKNHLTVQYGTFSLAKKLSGDAADDAKALNTDFNFRVNCGEAYNEVVKIKPGATWA
  2 WP_013188882.1  62.4%  35.2%     ---------------------------------------------------------------MANYMEVFSM-------
    consensus/100%               ...............................................................htsY.EVhph.......
    consensus/90%                ...............................................................htsY.EVhph.......
    consensus/80%                ...............................................................htsY.EVhph.......
    consensus/70%                ...............................................................htsY.EVhph.......

                    cov    pid  2241         :         .         .         .         .         3         .         . 2320
  1 WP_004013458.1 100.0% 100.0%     LPDGKVKVGSTCTVHELPAEIPGLSSTFAGWEGNGYTPADDNGITVNVAKTEPGAQPVV-FTANN--QADYNLGKFKIRK
  2 WP_013188882.1  62.4%  35.2%     -----------------------------------------SAIAKDPRQNQPGKPTLVSFKQKGKKGRNWTAGVIA---
    consensus/100%               .........................................suIshsstpspPGt.slV.Fptps..ttsashGhht...
    consensus/90%                .........................................suIshsstpspPGt.slV.Fptps..ttsashGhht...
    consensus/80%                .........................................suIshsstpspPGt.slV.Fptps..ttsashGhht...
    consensus/70%                .........................................suIshsstpspPGt.slV.Fptps..ttsashGhht...

                    cov    pid  2321         .         .         :         .         .         .         .         4 2400
  1 WP_004013458.1 100.0% 100.0%     SVVPQGTSFEGTFHFTYNCTPPAGKTVKPDLALNGEVDVPAGEASAEITVPADSTCTVTEADPKDAPKPVVADDKNPLKY
  2 WP_013188882.1  62.4%  35.2%     SAIAVAWAVGGLVMPSLAATPTAQASVKPTVNVSPDANFNMGTN---------------------------GNVRYRLAY
    consensus/100%               Ssls.uhuhtGhhh.ohssTPsAttoVKPslslss-sshshGps...........................uss+..LtY
    consensus/90%                Ssls.uhuhtGhhh.ohssTPsAttoVKPslslss-sshshGps...........................uss+..LtY
    consensus/80%                Ssls.uhuhtGhhh.ohssTPsAttoVKPslslss-sshshGps...........................uss+..LtY
    consensus/70%                Ssls.uhuhtGhhh.ohssTPsAttoVKPslslss-sshshGps...........................uss+..LtY

                    cov    pid  2401         .         .         .         .         :         .         .         . 2480
  1 WP_004013458.1 100.0% 100.0%     QGTSFQGVPGLASKAITVGKDQVIEFEAVNKYVSQEGGFEFFKSVVGNNASKHTQDKFNFHWYCQARDKQVFSGSQNLSD
  2 WP_013188882.1  62.4%  35.2%     TGQLFSTKDPQEAAA---------------HY--------------GD---TAQVGDFTAHYL-----------------
    consensus/100%               pGp.Fpshss.tutA...............+Y..............Gs...ptp.scFshHah.................
    consensus/90%                pGp.Fpshss.tutA...............+Y..............Gs...ptp.scFshHah.................
    consensus/80%                pGp.Fpshss.tutA...............+Y..............Gs...ptp.scFshHah.................
    consensus/70%                pGp.Fpshss.tutA...............+Y..............Gs...ptp.scFshHah.................

                    cov    pid  2481         .         5         .         .         .         .         :         . 2560
```

```
1 WP_004013458.1 100.0% 100.0%      KGNKKVTKLPVGTKCALWEDSFKKLEDEAKPETKWLQGSSEIKTEKVKDAQGNTHDGVNFEIKSEKDTFSVTAQNKFDIP
2 WP_013188882.1  62.4%  35.2%      -----------PKECITKANRFDTMGD-MKGTPRWDGYDA-L--EQV---------------------------------
  consensus/100%                    ...........spcChhhtspFcphtD.hKsps+W.thsu.l..EpV.................................
  consensus/90%                     ...........spcChhhtspFcphtD.hKsps+W.thsu.l..EpV.................................
  consensus/80%                     ...........spcChhhtspFcphtD.hKsps+W.thsu.l..EpV.................................
  consensus/70%                     ...........spcChhhtspFcphtD.hKsps+W.thsu.l..EpV.................................

                 cov     pid 2561          .         .         .         6         .         .         .         . 2640
1 WP_004013458.1 100.0% 100.0%      STELKVKKLVKADKASKVAAGKQFTVNVTCVYPTDGQTYTIADAEKDGVFKHNQEKTFKTDKSGKNPIPVGAICTVTEDV
2 WP_013188882.1  62.4%  35.2%      -------------------------------APDRG-------------------KIAQKPLGQNAPMGFG---------
  consensus/100%                    ...............................hPscG...................KhhppshutpsPhshG.........
  consensus/90%                     ...............................hPscG...................KhhppshutpsPhshG.........
  consensus/80%                     ...............................hPscG...................KhhppshutpsPhshG.........
  consensus/70%                     ...............................hPscG...................KhhppshutpsPhshG.........

                 cov     pid 2641          :         .         .         .         .         7         .         . 2720
1 WP_004013458.1 100.0% 100.0%      ASADVPGHNWSVQIADGSTVVKTHT-----------TRVKVPKAGKTVTITNDYERKVGSFKLSKFVAGVDVNTALAAND
2 WP_013188882.1  62.4%  35.2%      --------AWVIFPGSSSATLPFNPSGSASRAATSDRSAAEPEEEDADTLTLSLEPIE----------GEATET--GATQ
  consensus/100%                    ........sWsl..usuSssl.hps...........ppst.PcttcssTlT.shE.h...........G.sspT..uAsp
  consensus/90%                     ........sWsl..usuSssl.hps...........ppst.PcttcssTlT.shE.h...........G.sspT..uAsp
  consensus/80%                     ........sWsl..usuSssl.hps...........ppst.PcttcssTlT.shE.h...........G.sspT..uAsp
  consensus/70%                     ........sWsl..usuSssl.hps...........ppst.PcttcssTlT.shE.h...........G.sspT..uAsp

                 cov     pid 2721          .         .         :         .         .         .         .         8 2800
1 WP_004013458.1 100.0% 100.0%      AKFEFKYNCKLDGKPVKDGTISLSATQMSTEITGIPAGAKCVVSEPKVKAPHGYAVPTLSWKLDGAQAADGTATITEGNT
2 WP_013188882.1  62.4%  35.2%      AR-IFGKT----ADPSGDSGTSTEPS-GAGNAPQLPANPEEPSSAP-----------ELSPESPA-PAAVGEDRAA-NPT
  consensus/100%                    A+..Fths....ucPstDushShpso.husphstlPAsscpssStP...........pLS.c.su..AAsGpsphs.ssT
  consensus/90%                     A+..Fths....ucPstDushShpso.husphstlPAsscpssStP...........pLS.c.su..AAsGpsphs.ssT
  consensus/80%                     A+..Fths....ucPstDushShpso.husphstlPAsscpssStP...........pLS.c.su..AAsGpsphs.ssT
  consensus/70%                     A+..Fths....ucPstDushShpso.husphstlPAsscpssStP...........pLS.c.su..AAsGpsphs.ssT

                 cov     pid 2801          .         .         .         .         :         .         .         . 2880
1 WP_004013458.1 100.0% 100.0%      STLDATNTYTPYQVKFDLK--KLV----KANDQAKLDGDFTFQVSCAQTSVD---------------------------V
2 WP_013188882.1  62.4%  35.2%      PTKTILSGAVPFGYKFEPTMKEIKLKFDPSVDKDTAIANFNKQVDEAQADVMNQLEQFMNGKPFDARTKKAMVDNLNLQF
  consensus/100%                    sThshhsshsPathKF-.p..clh.....usDpsph.usFshQVspAQssV............................h
  consensus/90%                     sThshhsshsPathKF-.p..clh.....usDpsph.usFshQVspAQssV............................h
  consensus/80%                     sThshhsshsPathKF-.p..clh.....usDpsph.usFshQVspAQssV............................h
  consensus/70%                     sThshhsshsPathKF-.p..clh.....usDpsph.usFshQVspAQssV............................h

                 cov     pid 2881          .         9         .         .         .         .         :         . 2960
1 WP_004013458.1 100.0% 100.0%      TLNQANDYSWDSSKFAGKPILSGE----TC----TIVETGTADIANYNHKSVEYTVSGSGVTKGNKAPAKGVSFTLPKTG
2 WP_013188882.1  62.4%  35.2%      DNLRKQGLAWFEEQYSKHPSASKFPGIKACEDNGPIVPVGSFILV-----------------K---------NWTAATKG
  consensus/100%                    s..ptpshuW.pppaut+P.hSt.....sC....sIV.sGoh.ls.................K.........saThsppG
  consensus/90%                     s..ptpshuW.pppaut+P.hSt.....sC....sIV.sGoh.ls.................K.........saThsppG
  consensus/80%                     s..ptpshuW.pppaut+P.hSt.....sC....sIV.sGoh.ls.................K.........saThsppG
  consensus/70%                     s..ptpshuW.pppaut+P.hSt.....sC....sIV.sGoh.ls.................K.........saThsppG

                 cov     pid 2961          .         .         .         0         .         .         .         . 3040
1 WP_004013458.1 100.0% 100.0%      DASVSV------TATNTYDHQLGSLQLTKAVSVKDNAANPWDNKSYGFTVDCYQPGEGEQVGEKLSTKAVSLKAGETKTV
2 WP_013188882.1  62.4%  35.2%      EPNVWQWARKHRNEGQAYHAAIDFLALYPS-----DTKNSF--KVDGQASFCVEPFYGYQIGNHKVYTD-----------
  consensus/100%                    -ssV........stspsYcttls.LtLh.u.....sstNsa..Ks.G.ss.ChpPh.G.QlGp+hshps...........
  consensus/90%                     -ssV........stspsYcttls.LtLh.u.....sstNsa..Ks.G.ss.ChpPh.G.QlGp+hshps...........
  consensus/80%                     -ssV........stspsYcttls.LtLh.u.....sstNsa..Ks.G.ss.ChpPh.G.QlGp+hshps...........
  consensus/70%                     -ssV........stspsYcttls.LtLh.u.....sstNsa..Ks.G.ss.ChpPh.G.QlGp+hshps...........

                 cov     pid 3041          :         .         .         .         .         1         .         . 3120
1 WP_004013458.1 100.0% 100.0%      VEKAKVGTVCFVKETVADTPD---------------------------------------VTNQLTWENAGTTVTSPDGV
2 WP_013188882.1  62.4%  35.2%      ---PSKSTIYWAKD-NPEKAETVKALAWHYAKYNRGANWGHYQNAIWMVIFGITDNGRAWNLDQMSWGDTGVTDVKDL-I
  consensus/100%                    ...sphuTlhasK-.ss-ps-.......................................shsQhoWtssGsTssps..l
  consensus/90%                     ...sphuTlhasK-.ss-ps-.......................................shsQhoWtssGsTssps..l
  consensus/80%                     ...sphuTlhasK-.ss-ps-.......................................shsQhoWtssGsTssps..l
  consensus/70%                     ...sphuTlhasK-.ss-ps-.......................................shsQhoWtssGsTssps..l

                 cov     pid 3121          .         .         :         .         .         .         .         2 3200
1 WP_004013458.1 100.0% 100.0%      ANARRVVIAEDQGKTPAMSVTATNRLEVHYGTFTLQKTLTGEAAADEDVTKTPVNFKVKCGDLSEQTVTVIPGGAPVDYA
2 WP_013188882.1  62.4%  35.2%      QQ----ALTAYQHRQDGNVVKAL--ETMHLDQVSLQPANDGSSL-----------LTVQL----------------NDYN
  consensus/100%                    tp....slst.Qt+psu.sVpAh...phHhspholQ.s.sGpuh...........hpVph................sDYs
  consensus/90%                     tp....slst.Qt+psu.sVpAh...phHhspholQ.s.sGpuh...........hpVph................sDYs
  consensus/80%                     tp....slst.Qt+psu.sVpAh...phHhspholQ.s.sGpuh...........hpVph................sDYs
  consensus/70%                     tp....slst.Qt+psu.sVpAh...phHhspholQ.s.sGpuh...........hpVph................sDYs

                 cov     pid 3201          .         .         .         .         :         .         .         . 3280
1 WP_004013458.1 100.0% 100.0%      TATGQKVKVGTACTVHEVPAKVTGVSSELTWQG----SGTPAADNGVSVEIPAVGAPNVAIKAVNNVTY---NKAAFKIK
2 WP_013188882.1  62.4%  35.2%      PNST--------------EGRIIGDQVYLTVQGATYADGKPIPNNRISLL-----------EALNGVTLKIANQNAFKIS
  consensus/100%                    ssos...............u+lhGsps.LThQG....sGpPhssNtlSl............cAlNsVTh...NpsAFKIp
  consensus/90%                     ssos...............u+lhGsps.LThQG....sGpPhssNtlSl............cAlNsVTh...NpsAFKIp
  consensus/80%                     ssos...............u+lhGsps.LThQG....sGpPhssNtlSl............cAlNsVTh...NpsAFKIp
  consensus/70%                     ssos...............u+lhGsps.LThQG....sGpPhssNtlSl............cAlNsVTh...NpsAFKIp

                 cov     pid 3281          .         3         .         .         .         .         :         . 3360
1 WP_004013458.1 100.0% 100.0%      KTVDPVATKFLGDFQFTYRCVAPNGKVYTQATPFNNQASAQYVSVAAGEESAEIIVPTDSKCTVTEADPQTTPAVDNKGA
2 WP_013188882.1  62.4%  35.2%      FAG------SVKNVEDPYFIDNPNGPTQAQVVVLRKDL-----DLSGSL---------EVKW---TYIPQPKPAIS----
  consensus/100%                    hss.......ltshp.sYhhssPNG.s.sQssshppph.....sluuu..........-sKh...ph.PQspPAls....
  consensus/90%                     hss.......ltshp.sYhhssPNG.s.sQssshppph.....sluuu..........-sKh...ph.PQspPAls....
  consensus/80%                     hss.......ltshp.sYhhssPNG.s.sQssshppph.....sluuu..........-sKh...ph.PQspPAls....
```

```
    consensus/70%                hss.......ltshp.sYhhssPNG.s.sQssshppph.....sluuu..........-sKh...ph.PQspPAls....

                     cov     pid 3361         .         .         .         4         .         .         .         . 3440
  1 WP_004013458.1 100.0% 100.0%      NPVKYSGTQFTPGGKGLTSASFTVTKGTEVVTATA--KNLYVPQTTGFQFSKAVSGNNVANHPGKFNFGYTCKLPNGQKT
  2 WP_013188882.1  62.4%  35.2%      TTAKVNGAESVPGGAETA--TFS--RADEVITLTDDVKYENIPADTGMN-------------RKYLIGSLTKVSEGIAK
    consensus/100%                    sssKhsGsp.sPGGtths..oFo..+usEVlThTs..K...lPtsTGhp..............tKa.hG.hsKlspG.tp
    consensus/90%                     sssKhsGsp.sPGGtths..oFo..+usEVlThTs..K...lPtsTGhp..............tKa.hG.hsKlspG.tp
    consensus/80%                     sssKhsGsp.sPGGtths..oFo..+usEVlThTs..K...lPtsTGhp..............tKa.hG.hsKlspG.tp
    consensus/70%                     sssKhsGsp.sPGGtths..oFo..+usEVlThTs..K...lPtsTGhp..............tKa.hG.hsKlspG.tp

                     cov     pid 3441         :         .         .         .         .         5         .         . 3520
  1 WP_004013458.1 100.0% 100.0%      AVKTEQLGAN--D---------PAVNVTDLPVGTECVLWEESVEKTQPSEK-ISTTWTVGAAKPVVGSKQADHQGTPHDN
  2 WP_013188882.1  62.4%  35.2%      KVLTQVADADISDQGTVTGLFPYEVKASEVKKGD-RFYYEEILVTSDPKYAGGETAFTKGYGNLNV---IAKHTGENPDQ
    consensus/100%                    tVhTp.hsAs..D..........tVpso-l.hGs.phhaEE.l.popPp.t.hpTsaThGhup.sV....AcHpGps.Dp
    consensus/90%                     tVhTp.hsAs..D..........tVpso-l.hGs.phhaEE.l.popPp.t.hpTsaThGhup.sV....AcHpGps.Dp
    consensus/80%                     tVhTp.hsAs..D..........tVpso-l.hGs.phhaEE.l.popPp.t.hpTsaThGhup.sV....AcHpGps.Dp
    consensus/70%                     tVhTp.hsAs..D..........tVpso-l.hGs.phhaEE.l.popPp.t.hpTsaThGhup.sV....AcHpGps.Dp

                     cov     pid 3521         .         .         :         .         .         .         .         6 3600
  1 WP_004013458.1 100.0% 100.0%      AVKFKVDKENEAVSITAENTFEVSGTELTVKKTVTKDNNSEIADSKSFKVSIKCVYPTDK-----AEHTIATNEPLTNGQ
  2 WP_013188882.1  62.4%  35.2%      TVLIAKNK-----------------AELTLSKEAIGA-ADKADGNVTFR--VSCDDGTAATLYAPARGGVANTNPAWAG-
    consensus/100%                    sVhhthsK.................sELTlpKpshts.sschssshoF+..lpCs.sTst.....ActslAsspPhhsG.
    consensus/90%                     sVhhthsK.................sELTlpKpshts.sschssshoF+..lpCs.sTst.....ActslAsspPhhsG.
    consensus/80%                     sVhhthsK.................sELTlpKpshts.sschssshoF+..lpCs.sTst.....ActslAsspPhhsG.
    consensus/70%                     sVhhthsK.................sELTlpKpshts.sschssshoF+..lpCs.sTst.....ActslAsspPhhsG.

                     cov     pid 3601         .         .         .         .         :         .         .         . 3680
  1 WP_004013458.1 100.0% 100.0%      SKTFKTDDTGTAIPVGAKCTVTEDETSAKVA---GHTWKL----EVKDPNQVSTKGL-------SAEKDKAKNTVELVND
  2 WP_013188882.1  62.4%  35.2%      -----YQASSIEVTPGAICTVEETYSPAPVDLATAPRWVGGNAEMVSDPTPVNVNGKTIGSKAKFKMGDAGDINLAVTNT
    consensus/100%                    .....hpsouhtlssGAhCTVpEs.osA.Vs...u.pWhh.....VpDPs.VsspGh........t.tDtuc.sltlsNs
    consensus/90%                     .....hpsouhtlssGAhCTVpEs.osA.Vs...u.pWhh.....VpDPs.VsspGh........t.tDtuc.sltlsNs
    consensus/80%                     .....hpsouhtlssGAhCTVpEs.osA.Vs...u.pWhh.....VpDPs.VsspGh........t.tDtuc.sltlsNs
    consensus/70%                     .....hpsouhtlssGAhCTVpEs.osA.Vs...u.pWhh.....VpDPs.VsspGh........t.tDtuc.sltlsNs

                     cov     pid 3681         .         7         .         .         .         .         :         . 3760
  1 WP_004013458.1 100.0% 100.0%      YKREVGGFKLTKAVAGKG-IDFAKAADANNGKFEFTYDCVLDG--KTVKNGKGKLDLTKDKQTVEVKDIPTDATCTLKET
  2 WP_013188882.1  62.4%  35.2%      YREPTGGFKVFKYVNSDGKFDAYTAANANGGKFNFYYRCDKGIAGSFALPEDPSTDADGDPWSYEVKDLPIGARCSVTEM
    consensus/100%                    Y+c.sGGFKlhKhVsucG.hDhhpAAsANsGKFpFhYcCshsh..phshstcsphDhstD..ohEVKDlPhsApColpEh
    consensus/90%                     Y+c.sGGFKlhKhVsucG.hDhhpAAsANsGKFpFhYcCshsh..phshstcsphDhstD..ohEVKDlPhsApColpEh
    consensus/80%                     Y+c.sGGFKlhKhVsucG.hDhhpAAsANsGKFpFhYcCshsh..phshstcsphDhstD..ohEVKDlPhsApColpEh
    consensus/70%                     Y+c.sGGFKlhKhVsucG.hDhhpAAsANsGKFpFhYcCshsh..phshstcsphDhstD..ohEVKDlPhsApColpEh

                     cov     pid 3761         .         .         .         8         .         .         .         . 3840
  1 WP_004013458.1 100.0% 100.0%      APTAPTGFAAPTAPTWKIGST--TSDSGMVTIKEGQTAEVVATNTYTPYQVKFTLKKVIKA--DKTLDGDFKFQVTCGDK
  2 WP_013188882.1  62.4%  35.2%      KPTAPSGYDVTISAADALVKPVQSNTVSLRPIRADFANQVSFMNQYLPADASFSLRKIARFTDGQLINDNFALHYSCSDR
    consensus/100%                    tPTAPoGassshuss.tlsps..osssuhhsI+ts.sspVshhNpYhPhpspFoL+Klh+h..sphlsssFthphoCuD+
    consensus/90%                     tPTAPoGassshuss.tlsps..osssuhhsI+ts.sspVshhNpYhPhpspFoL+Klh+h..sphlsssFthphoCuD+
    consensus/80%                     tPTAPoGassshuss.tlsps..osssuhhsI+ts.sspVshhNpYhPhpspFoL+Klh+h..sphlsssFthphoCuD+
    consensus/70%                     tPTAPoGassshuss.tlsps..osssuhhsI+ts.sspVshhNpYhPhpspFoL+Klh+h..sphlsssFthphoCuD+

                     cov     pid 3841         :         .         .         .         .         9         .         . 3920
  1 WP_004013458.1 100.0% 100.0%      TQSVTLNKDNKYTWESSELKTPQILSGVKCHLEETSEQAFKSHTWKSLDYQVDGKGVDTSVDKDAKRVTFTLPATGDADV
  2 WP_013188882.1  62.4%  35.2%      SGKTV-SGDVTVAGNGEAVSVKGVKAGMTCTITEPRKPAIAKANFKDLSFDLSGTQYNVS---NSERLEFDVPRNPNAVV
    consensus/100%                    otpss.stDsphshpuptlps.tlhuGhpCplpEspc.AhtptsaKsLsaplsGpthssS...sucRlpFslPtsssAsV
    consensus/90%                     otpss.stDsphshpuptlps.tlhuGhpCplpEspc.AhtptsaKsLsaplsGpthssS...sucRlpFslPtsssAsV
    consensus/80%                     otpss.stDsphshpuptlps.tlhuGhpCplpEspc.AhtptsaKsLsaplsGpthssS...sucRlpFslPtsssAsV
    consensus/70%                     otpss.stDsphshpuptlps.tlhuGhpCplpEspc.AhtptsaKsLsaplsGpthssS...sucRlpFslPtsssAsV

                     cov     pid 3921         .         .         :         .         .         .         .         0 4000
  1 WP_004013458.1 100.0% 100.0%      TITATNTYDRNKGALQLQKVAKLNGQDVSKDAKANPLPNHQYQFEVTCYDPADNGLGAKTQAVTVPLQAGAPPQTVIKDA
  2 WP_013188882.1  62.4%  35.2%      LVTATNVYEQKAGSFTLQKTVK--------AGESNPLKGTAFTFRISCGEEDPF---------TVSLKAG--QTWTSKSY
    consensus/100%                    hlTATNsY-pptGuhpLQKssK........sucuNPL.sptapFcloCh-.ss..........TVsLpAG...phs.Ksh
    consensus/90%                     hlTATNsY-pptGuhpLQKssK........sucuNPL.sptapFcloCh-.ss..........TVsLpAG...phs.Ksh
    consensus/80%                     hlTATNsY-pptGuhpLQKssK........sucuNPL.sptapFcloCh-.ss..........TVsLpAG...phs.Ksh
    consensus/70%                     hlTATNsY-pptGuhpLQKssK........sucuNPL.sptapFcloCh-.ss..........TVsLpAG...phs.Ksh

                     cov     pid 4001         .         .         .         .         :         .         .         . 4080
  1 WP_004013458.1 100.0% 100.0%      PVGSVCFIKETPVKTEAVIGTLSWEGDGTNAVGPDNAANVRKVVIGGEKGDVQQVNVKAVNDLTVQYGIFSLHKELTGDA
  2 WP_013188882.1  62.4%  35.2%      PVGTECLIHEYDPSTQAATSTVSYQGAMPM--GTTDA------VLT-VKEGKAAVKVEAVNTVKANYGKFALTKKVEGDA
    consensus/100%                    PVGo.ChI+EhsspTpAshuTlSapGshs...GsssA......Vls..KtshttVpVcAVNslpspYGhFuLpKclpGDA
    consensus/90%                     PVGo.ChI+EhsspTpAshuTlSapGshs...GsssA......Vls..KtshttVpVcAVNslpspYGhFuLpKclpGDA
    consensus/80%                     PVGo.ChI+EhsspTpAshuTlSapGshs...GsssA......Vls..KtshttVpVcAVNslpspYGhFuLpKclpGDA
    consensus/70%                     PVGo.ChI+EhsspTpAshuTlSapGshs...GsssA......Vls..KtshttVpVcAVNslpspYGhFuLpKclpGDA

                     cov     pid 4081         .         1         .         .         .         .         :         . 4160
  1 WP_004013458.1 100.0% 100.0%      QNDKDVKSKDFEFTVKCPGLDD--QKVTLKGGETKTLPEGKMKVGTKCSVHEVPATVAGVTFDPVSWSQNATARTDG---
  2 WP_013188882.1  62.4%  35.2%      KAA----ATTFAFDYKCNDAAHTNGTLEVRGGQTKTVG-DNILVGTTCYIKEKPVQIDGVTVTP-SWVTNLPNGTGAYAS
    consensus/100%                    pss....upsFtFshKCsshsc..tplpl+GGpTKTls.sphhVGTpC.l+EhPsplsGVThsP.SWspNhsstTsu...
    consensus/90%                     pss....upsFtFshKCsshsc..tplpl+GGpTKTls.sphhVGTpC.l+EhPsplsGVThsP.SWspNhsstTsu...
    consensus/80%                     pss....upsFtFshKCsshsc..tplpl+GGpTKTls.sphhVGTpC.l+EhPsplsGVThsP.SWspNhsstTsu...
    consensus/70%                     pss....upsFtFshKCsshsc..tplpl+GGpTKTls.sphhVGTpC.l+EhPsplsGVThsP.SWspNhsstTsu...

                     cov     pid 4161         .         .         .         2         .         .         .         . 4240
  1 WP_004013458.1 100.0% 100.0%      GIDVTI--PATG-VKDLSFTATNKAVYDKATFQIQKVISSTENLAFEDGFKFTYTCKAPNGKTFTQKAPFGAQTGVDTIN
  2 WP_013188882.1  62.4%  35.2%      YKQFTIPQPQNGKAQVLQLEAKNTLEYDKASFTIKKVIQGGAVFTPDATFKFTYSCKAPDGKTYTQATPFGSQAKAKFIE
```

6/17/2018 MView

```
   consensus/100%                hhphTI..PtsG.spsLphpApNph.YDKAoFpIpKVIpustshs.-ssFKFTYoCKAPsGKTaTQtsPFGuQstschIp
   consensus/90%                 hhphTI..PtsG.spsLphpApNph.YDKAoFpIpKVIpustshs.-ssFKFTYoCKAPsGKTaTQtsPFGuQstschIp
   consensus/80%                 hhphTI..PtsG.spsLphpApNph.YDKAoFpIpKVIpustshs.-ssFKFTYoCKAPsGKTaTQtsPFGuQstschIp
   consensus/70%                 hhphTI..PtsG.spsLphpApNph.YDKAoFpIpKVIpustshs.-ssFKFTYoCKAPsGKTaTQtsPFGuQstschIp

                       cov    pid 4241          :         .         .         .         .         3         .         . 4320
1 WP_004013458.1 100.0% 100.0%    VKAGQPSGIITVPQGSKCTVSEANPKD---LPKLVNDPNRNPVKYQENGT-----SIKVGDKTVTGLTSAEFTADGQMVT
2 WP_013188882.1  62.4%  35.2%    VAAGQESAAIVVPQGSKCSVSEPQAQLYVTNQKLVNNPNANPLKYVSTSFVPEGKGLTSGEQT---------VTDSQTFQ
   consensus/100%                 VtAGQ.SuhIsVPQGSKCoVSEspsp......KLVNsPNtNPlKY.psuh.....ulpsG-pT.........ssDuQhhp
   consensus/90%                  VtAGQ.SuhIsVPQGSKCoVSEspsp......KLVNsPNtNPlKY.psuh.....ulpsG-pT.........ssDuQhhp
   consensus/80%                  VtAGQ.SuhIsVPQGSKCoVSEspsp......KLVNsPNtNPlKY.psuh.....ulpsG-pT.........ssDuQhhp
   consensus/70%                  VtAGQ.SuhIsVPQGSKCoVSEspsp......KLVNsPNtNPlKY.psuh.....ulpsG-pT.........ssDuQhhp

                       cov    pid 4321          .         .         :         .         .         .         .         4 4400
1 WP_004013458.1 100.0% 100.0%    VIATNTYVEKITGFQFKKTLDGNNAGKYQDQQFSFGYTCKTPKEVRSG--DAKLKSADDPVKVENLPVGTECTLWENKVN
2 WP_013188882.1  62.4%  35.2%    FAATNVYTEKMTGFKFYKAAVTGNNAGNHTEQFYFNYSCQTPAGEVKNGVGYDLGAGSDPVTVENLPVGTKCVVWEKPAV
   consensus/100%                 hhATNsYsEKhTGFpFhKshsssNsut.pspQF.FsYoCpTPtt.hps..shcLtuusDPVpVENLPVGTcCslWEp.ss
   consensus/90%                  hhATNsYsEKhTGFpFhKshsssNsut.pspQF.FsYoCpTPtt.hps..shcLtuusDPVpVENLPVGTcCslWEp.ss
   consensus/80%                  hhATNsYsEKhTGFpFhKshsssNsut.pspQF.FsYoCpTPtt.hps..shcLtuusDPVpVENLPVGTcCslWEp.ss
   consensus/70%                  hhATNsYsEKhTGFpFhKshsssNsut.pspQF.FsYoCpTPtt.hps..shcLtuusDPVpVENLPVGTcCslWEp.ss

                       cov    pid 4401          .         .         .         .         :         .         .         . 4480
1 WP_004013458.1 100.0% 100.0%    PKNAEKVSTTWTIDGK-PVSSGSVTDHNGVKHTNANGVTFKVETEGTPVNVVATNKFDIPSTSFKIHKTVTNKTDGYHVP
2 WP_013188882.1  62.4%  35.2%    AQANEKVTTKWTVGTAEPVEGKDVKPFDNNARTQANGVAFTVDQENQALMVGATNDFTVPDTKLVVSKTIVAGE--NTTV
   consensus/100%                 spssEKVoTpWTlsst.PVputsVpsassst+TpANGVsFpV-pEspsl.VsATNcFslPsTphhlpKTlsstp...pss
   consensus/90%                  spssEKVoTpWTlsst.PVputsVpsassst+TpANGVsFpV-pEspsl.VsATNcFslPsTphhlpKTlsstp...pss
   consensus/80%                  spssEKVoTpWTlsst.PVputsVpsassst+TpANGVsFpV-pEspsl.VsATNcFslPsTphhlpKTlsstp...pss
   consensus/70%                  spssEKVoTpWTlsst.PVputsVpsassst+TpANGVsFpV-pEspsl.VsATNcFslPsTphhlpKTlsstp...pss

                       cov    pid 4481          .         5         .         .         .         .         :         . 4560
1 WP_004013458.1 100.0% 100.0%    ADKGFKFDVTCTYPTDDAEHVIAKDELVKGGQDSKNFEADVDGVPIPVGASCTVTEKNAA--VANYSLAVTMKDGGKALT
2 WP_013188882.1  62.4%  35.2%    GNKSVKLSATCKYPTDNAEHVIMDGKTFKNG-DSAEFTTDVNGVKIPVGATCTISESEPSAKVAGHLWTVQMKEGKRVLS
   consensus/100%                 usKuhKhssTCpYPTDsAEHVIhcschhKsG.DStpFpsDVsGV.IPVGAoCTloEppsu..VAsa.hsVpMK-Gt+sLo
   consensus/90%                  usKuhKhssTCpYPTDsAEHVIhcschhKsG.DStpFpsDVsGV.IPVGAoCTloEppsu..VAsa.hsVpMK-Gt+sLo
   consensus/80%                  usKuhKhssTCpYPTDsAEHVIhcschhKsG.DStpFpsDVsGV.IPVGAoCTloEppsu..VAsa.hsVpMK-Gt+sLo
   consensus/70%                  usKuhKhssTCpYPTDsAEHVIhcschhKsG.DStpFpsDVsGV.IPVGAoCTloEppsu..VAsa.hsVpMK-Gt+sLo

                       cov    pid 4561          .         .         .         6         .         .         .         . 4640
1 WP_004013458.1 100.0% 100.0%    VETKQADQTKSEPGYTAKVITTVEAKTVNVENTYTRDFGIFKIEKKTTAQSYIEKPSSFDFKYTCKDPKDSKAEVKQGIL
2 WP_013188882.1  62.4%  35.2%    DKA-----------TATVTLNAEKGRTVEVINTYERELGKFKIMKSVSAAKNITVKESYSFSYTCADPQDATQKVAGKVT
   consensus/100%                 scs...........hsshslss.cu+TVpV.NTYpR-hGhFKI.KpsoAtp.Iph.pSasFpYTCtDPpDuptcVtttlh
   consensus/90%                  scs...........hsshslss.cu+TVpV.NTYpR-hGhFKI.KpsoAtp.Iph.pSasFpYTCtDPpDuptcVtttlh
   consensus/80%                  scs...........hsshslss.cu+TVpV.NTYpR-hGhFKI.KpsoAtp.Iph.pSasFpYTCtDPpDuptcVtttlh
   consensus/70%                  scs...........hsshslss.cu+TVpV.NTYpR-hGhFKI.KpsoAtp.Iph.pSasFpYTCtDPpDuptcVtttlh

                       cov    pid 4641          :         .         .         .         .         7         .         . 4720
1 WP_004013458.1 100.0% 100.0%    NVGEA-AVSSQQIPVGYKCEVSEVDPKV---KGAKWAHTLSQKTFVIQKSEVQTASLTATNDFTDSTGKFKVTKKVKNDD
2 WP_013188882.1  62.4%  35.2%    GVTDKGYKIVDGIPLGYKCHITEDQAGAKTEEGSTLSATLSANDFVVEKDNVVDV--SVTNTYSKANGKFSVKKAIAKDS
   consensus/100%                 sVs-t.hh.sptIPlGYKCcloEspsts...cGuphutTLStpsFVlpKspV.ss..osTNsaocusGKFpVpKtltpDs
   consensus/90%                  sVs-t.hh.sptIPlGYKCcloEspsts...cGuphutTLStpsFVlpKspV.ss..osTNsaocusGKFpVpKtltpDs
   consensus/80%                  sVs-t.hh.sptIPlGYKCcloEspsts...cGuphutTLStpsFVlpKspV.ss..osTNsaocusGKFpVpKtltpDs
   consensus/70%                  sVs-t.hh.sptIPlGYKCcloEspsts...cGuphutTLStpsFVlpKspV.ss..osTNsaocusGKFpVpKtltpDs

                       cov    pid 4721          .         .         :         .         .         .         .         8 4800
1 WP_004013458.1 100.0% 100.0%    KLALPLNATYNFEWWCGPKGAE---APQKWNTFSLKQGETFESGEIASGSECGIREVKPTGDFAAVGANVEVTWSLAETK
2 WP_013188882.1  62.4%  35.2%    T-AAIADGTYNFEYWCVTPDGSEVGTAAAPNKFSIKAGQTWTSGNIATGSKCAIREVTPSGTTT--GVAAEVSWSLQEST
   consensus/100%                 p.Ah.hsuTYNFEaWCss.sup...sstt.NpFSlKtGpTapSGpIAoGScCuIREVpPoGshs..GsssEVoWSLtEop
   consensus/90%                  p.Ah.hsuTYNFEaWCss.sup...sstt.NpFSlKtGpTapSGpIAoGScCuIREVpPoGshs..GsssEVoWSLtEop
   consensus/80%                  p.Ah.hsuTYNFEaWCss.sup...sstt.NpFSlKtGpTapSGpIAoGScCuIREVpPoGshs..GsssEVoWSLtEop
   consensus/70%                  p.Ah.hsuTYNFEaWCss.sup...sstt.NpFSlKtGpTapSGpIAoGScCuIREVpPoGshs..GsssEVoWSLtEop

                       cov    pid 4801          .         .         .         .         :         .         .         . 4880
1 WP_004013458.1 100.0% 100.0%    TTQGATSVKAPVLVDSEQAFVFGKQGDSAGKFTIGKNGSEQILTATNEVKQTYVDLSLEKVAQSSNNNGDPGLSVDKFNY
2 WP_013188882.1  62.4%  35.2%    TMQGVEPTP-YQAVGAENNFVFGAEGSTAGKFTIGKNGTTTGLTATNKLSYEEVKLKLQKIAEATSAHGRTDYSQTNFEN
   consensus/100%                 ThQGspss....hVsuEpsFVFGtpGsoAGKFTIGKNGopphLTATNclp.p.VcLpLpKlApuosspGcsshS.spFp.
   consensus/90%                  ThQGspss....hVsuEpsFVFGtpGsoAGKFTIGKNGopphLTATNclp.p.VcLpLpKlApuosspGcsshS.spFp.
   consensus/80%                  ThQGspss....hVsuEpsFVFGtpGsoAGKFTIGKNGopphLTATNclp.p.VcLpLpKlApuosspGcsshS.spFp.
   consensus/70%                  ThQGspss....hVsuEpsFVFGtpGsoAGKFTIGKNGopphLTATNclp.p.VcLpLpKlApuosspGcsshS.spFp.

                       cov    pid 4881          .         9         .         .         .         .         :         . 4960
1 WP_004013458.1 100.0% 100.0%    WNSYYQAWGGFVTRNFVEMQVTCNDKPVADKAHPEGIWKVPLNGKIDLPVKVAIGSKCTVAELD-KGFNDYKMLFYGRWR
2 WP_013188882.1  62.4%  35.2%    MKQ-YGAYYRYMNRDYVNMHVTCKNGAKTVV---DKTVQVNVNGDALDFGMVPYNSKCTVAELDDESFNDWNAMQYGGWR
   consensus/100%                 hpp.YtAahtahsRsaVpMpVTCpstshssh...-thhpVslNGch...shVshsSKCTVAELD.cuFNDaphh.YGtWR
   consensus/90%                  hpp.YtAahtahsRsaVpMpVTCpstshssh...-thhpVslNGch...shVshsSKCTVAELD.cuFNDaphh.YGtWR
   consensus/80%                  hpp.YtAahtahsRsaVpMpVTCpstshssh...-thhpVslNGch...shVshsSKCTVAELD.cuFNDaphh.YGtWR
   consensus/70%                  hpp.YtAahtahsRsaVpMpVTCpstshssh...-thhpVslNGch...shVshsSKCTVAELD.cuFNDaphh.YGtWR

                       cov    pid 4961          .         .         .         0         .         .         .         . 5040
1 WP_004013458.1 100.0% 100.0%    HSVDMSAKSLDGI--DVSKRYDFDAVEDDKAQAAIDFDVSGKTKVEVKMTNNWDCFSKVTSELGTNLTDA----KK--VN
2 WP_013188882.1  62.4%  35.2%    HKAVITATDASGKALASEVNYAFDMMSSDGEQAAITFDVTSEADVSVLMRNRWDCFASIESVLDAPDHEAVADGKRVLKN
   consensus/100%                 HpsshoApshsGh..ssphpYsFDhhpsDttQAAIsFDVoucscVpVhMpNpWDCFuplpS.Lsss.p-A....K+..hN
   consensus/90%                  HpsshoApshsGh..ssphpYsFDhhpsDttQAAIsFDVoucscVpVhMpNpWDCFuplpS.Lsss.p-A....K+..hN
   consensus/80%                  HpsshoApshsGh..ssphpYsFDhhpsDttQAAIsFDVoucscVpVhMpNpWDCFuplpS.Lsss.p-A....K+..hN
   consensus/70%                  HpsshoApshsGh..ssphpYsFDhhpsDttQAAIsFDVoucscVpVhMpNpWDCFuplpS.Lsss.p-A....K+..hN
```

```
                    cov    pid 5041         :         .         .         .         .         1         .         . 5120
  1 WP_004013458.1 100.0% 100.0%      GVRQIDLSEKKSDGTNKYTDWVTLTDVVEFKNL-PKRNYTVMGRLFNASKSGKLTAADFNNPNQYLNWKNPQGHTWNYVY
  2 WP_013188882.1  62.4%  35.2%      GIKVINVD--------SETGRIKLTDTVTLKNVTANRTYTVVERLYSTSKAGDRNR----FDQGYLTWESENGETWDYYA
    consensus/100%                    Gl+.Isls........p.TshlpLTDsVphKNl.spRsYTVhtRLassSKuGchst.....sptYLsWcs.pGcTWsYhh
    consensus/90%                     Gl+.Isls........p.TshlpLTDsVphKNl.spRsYTVhtRLassSKuGchst.....sptYLsWcs.pGcTWsYhh
    consensus/80%                     Gl+.Isls........p.TshlpLTDsVphKNl.spRsYTVhtRLassSKuGchst.....sptYLsWcs.pGcTWsYhh
    consensus/70%                     Gl+.Isls........p.TshlpLTDsVphKNl.spRsYTVhtRLassSKuGchst.....sptYLsWcs.pGcTWsYhh

                    cov    pid 5121         .         .         :         .         .         .         .         2 5200
  1 WP_004013458.1 100.0% 100.0%      QEVNADS----AKVTLTYEIPVSLFEANPDGIAAGVAVYRSDY--WNPQCAGNCGLMAVEPTLPQNQQVKPIWKGELKTQ
  2 WP_013188882.1  62.4%  35.2%      EEVTVPGGANDFTTDISFTIPVELLKKHPEGIAAGIMVFNGGNPMNKSQACARCLAYGSELKLPAGQSIVPQWAPTMSTV
    consensus/100%                    pEVsssu....hpssloapIPVpLhctpP-GIAAGlhVapus....psQssupChhhusE.pLPtsQplhP.WtsphpT.
    consensus/90%                     pEVsssu....hpssloapIPVpLhctpP-GIAAGlhVapus....psQssupChhhusE.pLPtsQplhP.WtsphpT.
    consensus/80%                     pEVsssu....hpssloapIPVpLhctpP-GIAAGlhVapus....psQssupChhhusE.pLPtsQplhP.WtsphpT.
    consensus/70%                     pEVsssu....hpssloapIPVpLhctpP-GIAAGlhVapus....psQssupChhhusE.pLPtsQplhP.WtsphpT.

                    cov    pid 5201         .         .         .         .         :         .         .         . 5280
  1 WP_004013458.1 100.0% 100.0%      VLNPVTKSNMLQVGKGAPEIQSIADTVTYTNVAPGSYDLYGRIVALGNEGLVIGEGTANVTTAEAGSNALSSGEWNNDQI
  2 WP_013188882.1  62.4%  35.2%      AYDRTTDSRVLSTNEKEVPTQKLTDNLTYTKVPAGDYYLYGRLVQTDDTSKSVVDNVKKIT-VPAGQNFFGKNRVD----
    consensus/100%                    shs.sTcSphLpssctts.hQplsDslTYTpVssGsY.LYGRlVthsspuhsls-sstplT.s.AGpNhhupschs....
    consensus/90%                     shs.sTcSphLpssctts.hQplsDslTYTpVssGsY.LYGRlVthsspuhsls-sstplT.s.AGpNhhupschs....
    consensus/80%                     shs.sTcSphLpssctts.hQplsDslTYTpVssGsY.LYGRlVthsspuhsls-sstplT.s.AGpNhhupschs....
    consensus/70%                     shs.sTcSphLpssctts.hQplsDslTYTpVssGsY.LYGRlVthsspuhsls-sstplT.s.AGpNhhupschs....

                    cov    pid 5281         .         3         .         .         .         .         :         . 5360
  1 WP_004013458.1 100.0% 100.0%      KVNTASLEDAVKNDAKGFVTYEFLLPKGSATSGAAV-------ETLKGQAVASHVDDNDVMQQLFTPTLRTTATVGSENG
  2 WP_013188882.1  62.4%  35.2%      --NEFNLQTSQLTPGAEYVMFEYMLPANVDVNSLAGKTPAELQDMSKAVNLPFHAEVKDICQKLYTPKLKTNAAID----
    consensus/100%                    ..NphsLpsu.hssuttaVhaEahLPtssssuhAs.......-h.Ku.sls.Hs-spDlhQpLaTPpL+TsAsls....
    consensus/90%                     ..NphsLpsu.hssuttaVhaEahLPtssssuhAs.......-h.Ku.sls.Hs-spDlhQpLaTPpL+TsAsls....
    consensus/80%                     ..NphsLpsu.hssuttaVhaEahLPtssssuhAs.......-h.Ku.sls.Hs-spDlhQpLaTPpL+TsAsls....
    consensus/70%                     ..NphsLpsu.hssuttaVhaEahLPtssssuhAs.......-h.Ku.sls.Hs-spDlhQpLaTPpL+TsAsls....

                    cov    pid 5361         .         .         .         4         .         .         .         . 5440
  1 WP_004013458.1 100.0% 100.0%      TKTVDANATGTVEVFDKVEYRNLPGGKKYTVTGKLHFKSADGTDGGELPGVDSASGTIDLTSQKATEGHSGTVTLKFTVP
  2 WP_013188882.1  62.4%  35.2%      -KPVTPAATGTVKVKDKVDYFNLVAGKQYTLTGTLHVKAADGSDAGVLNDPGATKTETFTVPGKAGTLVSGSHTLTFTVK
    consensus/100%                    .KsVsssATGTVcVhDKV-YhNLsuGKpYTlTGpLHhKuADGoDuG.Lsssuspsph.hsstKAsphhSGohTLpFTV.
    consensus/90%                     .KsVsssATGTVcVhDKV-YhNLsuGKpYTlTGpLHhKuADGoDuG.Lsssuspsph.hsstKAsphhSGohTLpFTV.
    consensus/80%                     .KsVsssATGTVcVhDKV-YhNLsuGKpYTlTGpLHhKuADGoDuG.Lsssuspsph.hsstKAsphhSGohTLpFTV.
    consensus/70%                     .KsVsssATGTVcVhDKV-YhNLsuGKpYTlTGpLHhKuADGoDuG.Lsssuspsph.hsstKAsphhSGohTLpFTV.

                    cov    pid 5441         :         .         .         .         .         5         .         . 5520
  1 WP_004013458.1 100.0% 100.0%      VEKLQQKIVVAFEEVKDGNILVAAHADITDEAQTVYSPKIGTTASAAPGFSATADTPA-TVDMSNAKPLKGKDSRKAELG
  2 WP_013188882.1  62.4%  35.2%      VASLKTQTVVAFEEVKDRGVVVATHAEITDEDQTVYNPSIGTTASVKDGVSDAADTRAATVDMTKAELVK-DSKTKKELS
    consensus/100%                    VtpLppphVVAFEEVKDtsllVAsHA-ITDEsQTVYsPpIGTTASstsGhSssADT.A.TVDMopAc.lK.csppKtELu
    consensus/90%                     VtpLppphVVAFEEVKDtsllVAsHA-ITDEsQTVYsPpIGTTASstsGhSssADT.A.TVDMopAc.lK.csppKtELu
    consensus/80%                     VtpLppphVVAFEEVKDtsllVAsHA-ITDEsQTVYsPpIGTTASstsGhSssADT.A.TVDMopAc.lK.csppKtELu
    consensus/70%                     VtpLppphVVAFEEVKDtsllVAsHA-ITDEsQTVYsPpIGTTASstsGhSssADT.A.TVDMopAc.lK.csppKtELu

                    cov    pid 5521         .         .         :         .         .         .         .         6 5600
  1 WP_004013458.1 100.0% 100.0%      NVAVIDTVEYHGLEAGKYVLYGELKKLSDGKEVSTAKTSS---HSFTVDAVTGVHGTQSVTFSDLKVQSGETYVVYEYLF
  2 WP_013188882.1  62.4%  35.2%      GVVVTDKVAYNSIAPGEYVMYGQLMTVGANGKLLSQTPVQTAFQTLSIAKGASASGTMELTFPGVKVVSGESYVVFEKLY
    consensus/100%                    sVsVhDpVtYpultsGcYVhYGpLhplusstcl.otpssp...poholstssuspGT.plTFsslKV.SGEoYVVaEhLa
    consensus/90%                     sVsVhDpVtYpultsGcYVhYGpLhplusstcl.otpssp...poholstssuspGT.plTFsslKV.SGEoYVVaEhLa
    consensus/80%                     sVsVhDpVtYpultsGcYVhYGpLhplusstcl.otpssp...poholstssuspGT.plTFsslKV.SGEoYVVaEhLa
    consensus/70%                     sVsVhDpVtYpultsGcYVhYGpLhplusstcl.otpssp...poholstssuspGT.plTFsslKV.SGEoYVVaEhLa

                    cov    pid 5601         .         .         .         .         :         .         .         . 5680
  1 WP_004013458.1 100.0% 100.0%      KAGDFQVDK---PDTKKAIASHADPEDKGQTVTVPGVTTDATDGDGDKYVDSSQNFTIKDTVTATGLIPGKTYDVSGELM
  2 WP_013188882.1  62.4%  35.2%      KTSDFDQAAGKPSNPKATVAEHADPKDSAQTVYVPGVTTDAKDGDGDKYVDPTQPIKIVDTVTGSGLIPGKEYTIDGELM
    consensus/100%                    KsuDFp.st...sssKtslApHADPcDpuQTVhVPGVTTDApDGDGDKYVDsoQshpIhDTVTuoGLIPGKpYslsGELM
    consensus/90%                     KsuDFp.st...sssKtslApHADPcDpuQTVhVPGVTTDApDGDGDKYVDsoQshpIhDTVTuoGLIPGKpYslsGELM
    consensus/80%                     KsuDFp.st...sssKtslApHADPcDpuQTVhVPGVTTDApDGDGDKYVDsoQshpIhDTVTuoGLIPGKpYslsGELM
    consensus/70%                     KsuDFp.st...sssKtslApHADPcDpuQTVhVPGVTTDApDGDGDKYVDsoQshpIhDTVTuoGLIPGKpYslsGELM

                    cov    pid 5681         .         7         .         .         .         .         :         . 5760
  1 WP_004013458.1 100.0% 100.0%      VDDGTPQGATTGIKQTG-TI--TAKADGTGETVLEFPVTAQQAQDLGL----------VGKPIVVFEDLSLDGKKVAVHH
  2 WP_013188882.1  62.4%  35.2%      MAGQNGEPVHTGVFNNGGDVKVVADANGKFQKDLTFEMSVEKAKELGLWVDENGQPTPTGKDLVVFENLKQGGKPVAIHH
    consensus/100%                    hsstsspsspTGlhpsG.sl..sAcAsGphppsLpF.hosppAp-LGL..........sGKslVVFEsLp.sGK.VAlHH
    consensus/90%                     hsstsspsspTGlhpsG.sl..sAcAsGphppsLpF.hosppAp-LGL..........sGKslVVFEsLp.sGK.VAlHH
    consensus/80%                     hsstsspsspTGlhpsG.sl..sAcAsGphppsLpF.hosppAp-LGL..........sGKslVVFEsLp.sGK.VAlHH
    consensus/70%                     hsstsspsspTGlhpsG.sl..sAcAsGphppsLpF.hosppAp-LGL..........sGKslVVFEsLp.sGK.VAlHH

                    cov    pid 5761         .         .         .         8         .         .         .         . 5840
  1 WP_004013458.1 100.0% 100.0%      DIKDEKQTVYNGGLKTKAVDAADENQAMVPGQKSAAVVDTVTFNGRFEKSHSYTLVGELHYVNGT---------VVPGTK
  2 WP_013188882.1  62.4%  35.2%      DITDEAQTVHSGSMGTSAK-DSDGNQTMIPGQENAVITDTVKFTGKFEPKHNYTLVGELHYVTTNPDGSKTDAGKVPGVT
    consensus/100%                    DIpDEtQTVasGuhtTpAh.suDtNQsMlPGQcsAslsDTVpFsG+FE.pHsYTLVGELHYVsss.........hVPGsp
    consensus/90%                     DIpDEtQTVasGuhtTpAh.suDtNQsMlPGQcsAslsDTVpFsG+FE.pHsYTLVGELHYVsss.........hVPGsp
    consensus/80%                     DIpDEtQTVasGuhtTpAh.suDtNQsMlPGQcsAslsDTVpFsG+FE.pHsYTLVGELHYVsss.........hVPGsp
    consensus/70%                     DIpDEtQTVasGuhtTpAh.suDtNQsMlPGQcsAslsDTVpFsG+FE.pHsYTLVGELHYVsss.........hVPGsp

                    cov    pid 5841         :         .         .         .         .         9         .         . 5920
  1 WP_004013458.1 100.0% 100.0%      TETKTFQSDQDGAIAAQKMTFTVPAEYIKAGQNMVVFEKLFDAKKKDGTPVASHEDPNDPDQTITVQEVEITTTAYDGA-
  2 WP_013188882.1  62.4%  35.2%      VQPVQVTSDAKGIIGKQTMKFTVAAKSIVEGKDLVVFETLHDGT-ATGKVVVKHANPNDPAQTINVVKNSITTTAYDGGK
    consensus/100%                    spshphpSDtcGhIutQpMpFTVsAc.IhtGpshVVFEpLaDup.tsGpsVspHtsPNDPsQTIsV.cspITTTAYDGu.
    consensus/90%                     spshphpSDtcGhIutQpMpFTVsAc.IhtGpshVVFEpLaDup.tsGpsVspHtsPNDPsQTIsV.cspITTTAYDGu.
```

```
   consensus/80%                spshphpSDtcGhIutQpMpFTVsAc.IhtGpshVVFEpLaDup.tsGpsVspHtsPNDPsQTIsV.cspITTTAYDGu.
   consensus/70%                spshphpSDtcGhIutQpMpFTVsAc.IhtGpshVVFEpLaDup.tsGpsVspHtsPNDPsQTIsV.cspITTTAYDGu.

                      cov    pid 5921         .         .         :         .         .         .         .         0 6000
 1 WP_004013458.1 100.0% 100.0%  AGDKSDPKDKNLDASKETVTIYDQVDYKGLNVGEEYTITGTLHYQADATLADGTQVKRGDEVPAQYVNVTPVKITANKAS
 2 WP_013188882.1  62.4%  35.2%  DGKGADPKDKNLDNSHAKVAIYDLVDYEGLQVGKEYTVTGTLHYQADATLADGTQVHTGDPIDAKYVTVTPAKFTAKTPS
   consensus/100%               sGctuDPKDKNLDsS+tpVsIYD.VDYcGLpVGcEYTlTGTLHYQADATLADGTQV+pGD.lsApYVsVTPsKhTAppsS
   consensus/90%                sGctuDPKDKNLDsS+tpVsIYD.VDYcGLpVGcEYTlTGTLHYQADATLADGTQV+pGD.lsApYVsVTPsKhTAppsS
   consensus/80%                sGctuDPKDKNLDsS+tpVsIYD.VDYcGLpVGcEYTlTGTLHYQADATLADGTQV+pGD.lsApYVsVTPsKhTAppsS
   consensus/70%                sGctuDPKDKNLDsS+tpVsIYD.VDYcGLpVGcEYTlTGTLHYQADATLADGTQV+pGD.lsApYVsVTPsKhTAppsS

                      cov    pid 6001         .         .         .         .         :         .         .         . 6080
 1 WP_004013458.1 100.0% 100.0%  SDESGAVKAIVKFEVQKTALATAPVVVFETLYQGTVEVATHQDIDDGSQVVYHPSLRTLATVNGAKVIQMKKDSKENLTV
 2 WP_013188882.1  62.4%  35.2%  SKDQGAVKAIVKFEVDKAALATAPVVVFEDLQDGTVTVATHHDINDDSQVVYNPSLATVATVNDGKLVQLKKDQK-AVTV
   consensus/100%               Sc-pGAVKAIVKFEVpKsALATAPVVVFEsL.pGTVpVATHpDIsDsSQVVYpPSLtTlATVNsuKllQhKKDpK.slTV
   consensus/90%                Sc-pGAVKAIVKFEVpKsALATAPVVVFEsL.pGTVpVATHpDIsDsSQVVYpPSLtTlATVNsuKllQhKKDpK.slTV
   consensus/80%                Sc-pGAVKAIVKFEVpKsALATAPVVVFEsL.pGTVpVATHpDIsDsSQVVYpPSLtTlATVNsuKllQhKKDpK.slTV
   consensus/70%                Sc-pGAVKAIVKFEVpKsALATAPVVVFEsL.pGTVpVATHpDIsDsSQVVYpPSLtTlATVNsuKllQhKKDpK.slTV

                      cov    pid 6081         .         1         .         .         .         .         :         . 6160
 1 WP_004013458.1 100.0% 100.0%  TDQITWANLAPGTYTLEGSLMEVKDGQLVSNTPVAKGQTQKVEVAAGKAGATTSTGEAQMTFKLPVDKVKSGSQFVVYQI
 2 WP_013188882.1  62.4%  35.2%  DDEVTITNAAPGTYTLKGELMSADGKTVISTSEVKN-----VKVTKGE------ATVQKVTFQVPANQVKAGAKFVVFEE
   consensus/100%               sDplThsNhAPGTYTLcGpLMpscstpllSso.Vtp.....VcVstGc......ss.tphTFplPsspVKuGupFVVap.
   consensus/90%                sDplThsNhAPGTYTLcGpLMpscstpllSso.Vtp.....VcVstGc......ss.tphTFplPsspVKuGupFVVap.
   consensus/80%                sDplThsNhAPGTYTLcGpLMpscstpllSso.Vtp.....VcVstGc......ss.tphTFplPsspVKuGupFVVap.
   consensus/70%                sDplThsNhAPGTYTLcGpLMpscstpllSso.Vtp.....VcVstGc......ss.tphTFplPsspVKuGupFVVap.

                      cov    pid 6161         .         .         .         2         .         .         .         . 6240
 1 WP_004013458.1 100.0% 100.0%  LKDKSGQVVATHADPKSDDQTVTVGSLDTTATDAADGNKHADNAAAVTINDKVDYSGLNLAATYPDGTLKAYLVRGELMD
 2 WP_013188882.1  62.4%  35.2%  LVDAKGVVVADHKDLASEAQTVRVGSLETTATDKADGDHVADNTKTVTIVDKVDYSGLNLDAKYADGTPQAYLLSGQLMD
   consensus/100%               LhDtpG.VVAsHtD.tS-sQTVpVGSL-TTATDtADGs+hADNstsVTIsDKVDYSGLNLsApYsDGT.pAYLlpGpLMD
   consensus/90%                LhDtpG.VVAsHtD.tS-sQTVpVGSL-TTATDtADGs+hADNstsVTIsDKVDYSGLNLsApYsDGT.pAYLlpGpLMD
   consensus/80%                LhDtpG.VVAsHtD.tS-sQTVpVGSL-TTATDtADGs+hADNstsVTIsDKVDYSGLNLsApYsDGT.pAYLlpGpLMD
   consensus/70%                LhDtpG.VVAsHtD.tS-sQTVpVGSL-TTATDtADGs+hADNstsVTIsDKVDYSGLNLsApYsDGT.pAYLlpGpLMD

                      cov    pid 6241         :         .         .         .         .         3         .         . 6320
 1 WP_004013458.1 100.0% 100.0%  KATGKPVAGVAPVERVIGAANSVYRV-GDQNRPVEEEITSGAGSVVLSFQVPAKLTQGKVLVAFETVYEEGREFLIHHDI
 2 WP_013188882.1  62.4%  35.2%  KQTKQAVPGLAPVERVIGPAGSVYRAEGQPDRPVEKVITSGTGFELLEFEVPANLILGKPVVVFETVYQEGKEYLIHHDI
   consensus/100%               KtTtpsVsGlAPVERVIGsAsSVYRs.Gp.sRPVEc.ITSGsG..lLpFpVPApLh.GKslVsFETVYpEG+EaLIHHDI
   consensus/90%                KtTtpsVsGlAPVERVIGsAsSVYRs.Gp.sRPVEc.ITSGsG..lLpFpVPApLh.GKslVsFETVYpEG+EaLIHHDI
   consensus/80%                KtTtpsVsGlAPVERVIGsAsSVYRs.Gp.sRPVEc.ITSGsG..lLpFpVPApLh.GKslVsFETVYpEG+EaLIHHDI
   consensus/70%                KtTtpsVsGlAPVERVIGsAsSVYRs.Gp.sRPVEc.ITSGsG..lLpFpVPApLh.GKslVsFETVYpEG+EaLIHHDI

                      cov    pid 6321         .         .         :         .         .         .         .         4 6400
 1 WP_004013458.1 100.0% 100.0%  NDDAQTVYTPSVKTQARVDSERNLLLADKDSTIKDTVTLSGLKTGETYVLSGVLMDKATGQPVLGKDMQAITAVSEPLKA
 2 WP_013188882.1  62.4%  35.2%  NDDSQTVWSPKLATKASVENERGLVIADAKSTVKDTVTFTGMKVGETYVVAGELWNKTQNK------STGIKVTSKPFKA
   consensus/100%               NDDuQTVaoPpltTpApV-sERsLllADtcSTlKDTVThoGhKsGETYVluG.LhsKspsp.......puIpssScPhKA
   consensus/90%                NDDuQTVaoPpltTpApV-sERsLllADtcSTlKDTVThoGhKsGETYVluG.LhsKspsp.......puIpssScPhKA
   consensus/80%                NDDuQTVaoPpltTpApV-sERsLllADtcSTlKDTVThoGhKsGETYVluG.LhsKspsp.......puIpssScPhKA
   consensus/70%                NDDuQTVaoPpltTpApV-sERsLllADtcSTlKDTVThoGhKsGETYVluG.LhsKspsp.......puIpssScPhKA

                      cov    pid 6401         .         .         .         .         :         .         .         . 6480
 1 WP_004013458.1 100.0% 100.0%  ESGAFVKTDAVSFTVPAGTVKADTELVVFEKLWVANEVTVDTKTKTVTPKDTKTGKSQPAASHEDITDENQTVKSGTSPS
 2 WP_013188882.1  62.4%  35.2%  DKASWTATDFMTFEVPAGTVAENDELVVFETLYRGDKVTN-----------------NPASTHKDPNDPAQTVGSGVKPA
   consensus/100%               -puuastTDhhoFpVPAGTVttssELVVFEpLahuscVTs.................pPAuoHcD.sD.sQTVtSGspPu
   consensus/90%                -puuastTDhhoFpVPAGTVttssELVVFEpLahuscVTs.................pPAuoHcD.sD.sQTVtSGspPu
   consensus/80%                -puuastTDhhoFpVPAGTVttssELVVFEpLahuscVTs.................pPAuoHcD.sD.sQTVtSGspPu
   consensus/70%                -puuastTDhhoFpVPAGTVttssELVVFEpLahuscVTs.................pPAuoHcD.sD.sQTVtSGspPu

                      cov    pid 6481         .         5         .         .         .         .         :         . 6560
 1 WP_004013458.1 100.0% 100.0%  LKTVLSADGKREWVENNTNIPTVPHASDSLIDTVLYTGLTEGVSYRLDAKLMEINPVTGKVSETPVATGYTEFTAKTSDG
 2 WP_013188882.1  62.4%  35.2%  IKTVLSTDGNRV---AGADIPTIPVDTTQLVDTVSYVGLTPGIKYTLNATLMEIGL-DGKVSDTG-ITGTATFTPTAANG
   consensus/100%               lKTVLSsDGpR....ssssIPTlPhsospLlDTV.YsGLT.GlpYpLsApLMEIs..sGKVS-Ts.hTGhspFTspsusG
   consensus/90%                lKTVLSsDGpR....ssssIPTlPhsospLlDTV.YsGLT.GlpYpLsApLMEIs..sGKVS-Ts.hTGhspFTspsusG
   consensus/80%                lKTVLSsDGpR....ssssIPTlPhsospLlDTV.YsGLT.GlpYpLsApLMEIs..sGKVS-Ts.hTGhspFTspsusG
   consensus/70%                lKTVLSsDGpR....ssssIPTlPhsospLlDTV.YsGLT.GlpYpLsApLMEIs..sGKVS-Ts.hTGhspFTspsusG

                      cov    pid 6561         .         .         .         6         .         .         .         . 6640
 1 WP_004013458.1 100.0% 100.0%  TAQVTFNGI-TGKLKAGYKYVAYEKMTRPGQPDKPVPPPHEDPKDPNQTVVSEHNPGITTTLTDAQAAKGTDGKVISLTR
 2 WP_013188882.1  62.4%  35.2%  TQKVGYNSFDKGILKPGYKYVAYETLIRNDKPNEP-PLKHEDPKDSDQTVKTDYHPSVVTDLTQDGSQ------V--IAT
   consensus/100%               TtpVsaNuh.pGhLKsGYKYVAYEphhRsspPscP.P..HEDPKDssQTVho-apPulsTsLTpstut......V..lsp
   consensus/90%                TtpVsaNuh.pGhLKsGYKYVAYEphhRsspPscP.P..HEDPKDssQTVho-apPulsTsLTpstut......V..lsp
   consensus/80%                TtpVsaNuh.pGhLKsGYKYVAYEphhRsspPscP.P..HEDPKDssQTVho-apPulsTsLTpstut......V..lsp
   consensus/70%                TtpVsaNuh.pGhLKsGYKYVAYEphhRsspPscP.P..HEDPKDssQTVho-apPulsTsLTpstut......V..lsp

                      cov    pid 6641         :         .         .         .         .         7         .         . 6720
 1 WP_004013458.1 100.0% 100.0%  DAQLKDVVRVTQTGLIEGAKYHVFSKLVNQANP--DQVVSAGMQEFTATGDQLRSVTVKFTVPKETLQE--LAGSDPSAE
 2 WP_013188882.1  62.4%  35.2%  DAALDLQDKVQMKGLVKGEKYVLVSRLVYNAGQPNETVVTDGLEKFTADSVNMTK-VIKFNVTTDQLKAPMAAAKAAGSE
   consensus/100%               DAtLc..s+Vp.pGLlcGtKYhlhS+LV.pAs...-pVVosGhpcFTAsusphpp.slKFsVsp-pLpt..hAupssuuE
   consensus/90%                DAtLc..s+Vp.pGLlcGtKYhlhS+LV.pAs...-pVVosGhpcFTAsusphpp.slKFsVsp-pLpt..hAupssuuE
   consensus/80%                DAtLc..s+Vp.pGLlcGtKYhlhS+LV.pAs...-pVVosGhpcFTAsusphpp.slKFsVsp-pLpt..hAupssuuE
   consensus/70%                DAtLc..s+Vp.pGLlcGtKYhlhS+LV.pAs...-pVVosGhpcFTAsusphpp.slKFsVsp-pLpt..hAupssuuE

                      cov    pid 6721         .         .         :         .         .         .         .         8 6800
 1 WP_004013458.1 100.0% 100.0%  FKLVAYEYLALDSDTDIVNKEATSEIEAVGFKTGKTWAATHADPNDAGQTVTVVKAPKIGTTLKYGQS-KTVWVADKVEL
```

```
2 WP_013188882.1   62.4%  35.2%      LKLVAYEYLTRASLVTGD--D---ATIKASVTDASKWDATHAQANDTRQTVTVVDVPRIGTKLQYAQGQKVVWEGDNVKL
  consensus/100%                     hKLVAYEYLshsS.sshs..-...th.tsuhpsuppWsATHApsNDstQTVTVVcsP+IGTpLpYuQu.KsVW.uDpVcL
  consensus/90%                      hKLVAYEYLshsS.sshs..-...th.tsuhpsuppWsATHApsNDstQTVTVVcsP+IGTpLpYuQu.KsVW.uDpVcL
  consensus/80%                      hKLVAYEYLshsS.sshs..-...th.tsuhpsuppWsATHApsNDstQTVTVVcsP+IGTpLpYuQu.KsVW.uDpVcL
  consensus/70%                      hKLVAYEYLshsS.sshs..-...th.tsuhpsuppWsATHApsNDstQTVTVVcsP+IGTpLpYuQu.KsVW.uDpVcL

                    cov    pid 6801         .         .         .         .         :         .         .         . 6880
1 WP_004013458.1 100.0% 100.0%      TDTVEYFNLQPKTKYTLSGNLMGGTSAESLSDTGVKATTEFTTPAAANGAQTVSGT------AVVKFTVPREVLERNEKL
2 WP_013188882.1   62.4%  35.2%      TDFVEYYNLLPATEYTLNGKLMGIATDGKVSDTGVVGTAKFTTPKATNGATRVSGTIADKTAAKVEFTVPLSVLKANDKL
  consensus/100%                     TDhVEYaNL.PtTcYTLsGpLMGhsostplSDTGVhuTscFTTPtAsNGAppVSGT......AhVcFTVPhpVLctN-KL
  consensus/90%                      TDhVEYaNL.PtTcYTLsGpLMGhsostplSDTGVhuTscFTTPtAsNGAppVSGT......AhVcFTVPhpVLctN-KL
  consensus/80%                      TDhVEYaNL.PtTcYTLsGpLMGhsostplSDTGVhuTscFTTPtAsNGAppVSGT......AhVcFTVPhpVLctN-KL
  consensus/70%                      TDhVEYaNL.PtTcYTLsGpLMGhsostplSDTGVhuTscFTTPtAsNGAppVSGT......AhVcFTVPhpVLctN-KL

                    cov    pid 6881         .         9         .         .         .         .         :         . 6960
1 WP_004013458.1 100.0% 100.0%      VAYEYLTIDGNPVASHEDPKDENQTVTSKKPGVGTYATVDKLKAFDVTDG-------KKDAFTIKDTVRLYNVEEGKTYA
2 WP_013188882.1   62.4%  35.2%      VAYEALLLKGQPVADHEDPNDENQTVKVKHPGVGTIAHVGDDTNLEVWEGVNQEGKKAPETYTVHDVIRMVNVEEGKTYA
  consensus/100%                     VAYEhLhlcGpPVAsHEDPpDENQTVpsK+PGVGThApVsc.psh-Vh-G.......t.-saTl+DslRhhNVEEGKTYA
  consensus/90%                      VAYEhLhlcGpPVAsHEDPpDENQTVpsK+PGVGThApVsc.psh-Vh-G.......t.-saTl+DslRhhNVEEGKTYA
  consensus/80%                      VAYEhLhlcGpPVAsHEDPpDENQTVpsK+PGVGThApVsc.psh-Vh-G.......t.-saTl+DslRhhNVEEGKTYA
  consensus/70%                      VAYEhLhlcGpPVAsHEDPpDENQTVpsK+PGVGThApVsc.psh-Vh-G.......t.-saTl+DslRhhNVEEGKTYA

                    cov    pid 6961         .         .         .         0         .         .         .         . 7040
1 WP_004013458.1 100.0% 100.0%      IAGQLYEQSVAGD--EGSALAKAATTVKVTASMAKPATEVEKTKYGEDVKVYETEMDLTVKREDLTKNQVVKDDIALVVY
2 WP_013188882.1   62.4%  35.2%      LGGQVYSKNAYDANAQTPALAANAKTVKVTADMAKPATDAEKAQYGADVKVYETSMDIPVKAADIKT-----HGDKLVVF
  consensus/100%                     luGQlYppsshss..pssALAtsApTVKVTAsMAKPAT-sEKspYGtDVKVYETpMDlsVKttDlpp.....cs.tLVVa
  consensus/90%                      luGQlYppsshss..pssALAtsApTVKVTAsMAKPAT-sEKspYGtDVKVYETpMDlsVKttDlpp.....cs.tLVVa
  consensus/80%                      luGQlYppsshss..pssALAtsApTVKVTAsMAKPAT-sEKspYGtDVKVYETpMDlsVKttDlpp.....cs.tLVVa
  consensus/70%                      luGQlYppsshss..pssALAtsApTVKVTAsMAKPAT-sEKspYGtDVKVYETpMDlsVKttDlpp.....cs.tLVVa

                    cov    pid 7041         :         .         .         .         .         1         .         . 7120
1 WP_004013458.1 100.0% 100.0%      EQLWAEGTYEKVNDTEVTPKGKSEPVAKHNDPQSSSQSIT-AEPQFGSLKLTKTVTGWEDAFAKVARPEASYKFTVKCVQ
2 WP_013188882.1   62.4%  35.2%      EQLWAEGTYESADGGKVTPKGNHKPVAAHNEITADSQSINVNRPEFGSLTLTKAVTGWDENTANVERAKATYVFTVKCAQ
  consensus/100%                     EQLWAEGTYEpssscVTPKGppcPVAtHN-.pusSQSIs.scPpFGSLpLTKsVTGW--shApVtRscAoYhFTVKCsQ
  consensus/90%                      EQLWAEGTYEpssscVTPKGppcPVAtHN-.pusSQSIs.scPpFGSLpLTKsVTGW--shApVtRscAoYhFTVKCsQ
  consensus/80%                      EQLWAEGTYEpssscVTPKGppcPVAtHN-.pusSQSIs.scPpFGSLpLTKsVTGW--shApVtRscAoYhFTVKCsQ
  consensus/70%                      EQLWAEGTYEpssscVTPKGppcPVAtHN-.pusSQSIs.scPpFGSLpLTKsVTGW--shApVtRscAoYhFTVKCsQ

                    cov    pid 7121         .         .         :         .         .         .         .         2 7200
1 WP_004013458.1 100.0% 100.0%      KGSVDEFTLKEGEEKTVEGIPLGDTCTISEDVQGAVNQAGLKDTVKFTAVNGVTVDSQVNGEAVVKIGGTANGSDTVANV
2 WP_013188882.1   62.4%  35.2%      KGSVDTITLHEGGSHTIDGIPVGDKCTITEDVQHAELQAGLKDTVKFTETNGVTVESVKNGEGTVMVGGTSNGTDRVPNV
  consensus/100%                     KGSVDphTL+EGtp+Tl-GIPlGDpCTIoEDVQtA..QAGLKDTVKFTtsNGVTV-S.hNGEusVhlGGTuNGoDpVsNV
  consensus/90%                      KGSVDphTL+EGtp+Tl-GIPlGDpCTIoEDVQtA..QAGLKDTVKFTtsNGVTV-S.hNGEusVhlGGTuNGoDpVsNV
  consensus/80%                      KGSVDphTL+EGtp+Tl-GIPlGDpCTIoEDVQtA..QAGLKDTVKFTtsNGVTV-S.hNGEusVhlGGTuNGoDpVsNV
  consensus/70%                      KGSVDphTL+EGtp+Tl-GIPlGDpCTIoEDVQtA..QAGLKDTVKFTtsNGVTV-S.hNGEusVhlGGTuNGoDpVsNV

                    cov    pid 7201         .         .         .         .         :         .         .         . 7280
1 WP_004013458.1 100.0% 100.0%      EVTAENSFSYDPVITTNTISQFGKVLENGGVLTDTVTYKQMPAGNYVLHTYFVEMVKDEATGKTVAKKIDYVPSYVTEQT
2 WP_013188882.1   62.4%  35.2%      TVVADNKFTNEVTIGTNTTGQFGKKSSNGGKITDLVKYQNLQAGKYLLHTYFVEYDKAG-----QAHKIDYVPSEVREVT
  consensus/100%                     pVsA-NpFo.-ssIsTNThuQFGKh.pNGGhlTDhVpYpph.AGpYlLHTYFVEhsKst......A+KIDYVPS.VpE.T
  consensus/90%                      pVsA-NpFo.-ssIsTNThuQFGKh.pNGGhlTDhVpYpph.AGpYlLHTYFVEhsKst......A+KIDYVPS.VpE.T
  consensus/80%                      pVsA-NpFo.-ssIsTNThuQFGKh.pNGGhlTDhVpYpph.AGpYlLHTYFVEhsKst......A+KIDYVPS.VpE.T
  consensus/70%                      pVsA-NpFo.-ssIsTNThuQFGKh.pNGGhlTDhVpYpph.AGpYlLHTYFVEhsKst......A+KIDYVPS.VpE.T

                    cov    pid 7281         .         3         .         .         .         .         :         . 7360
1 WP_004013458.1 100.0% 100.0%      VKADATTPENGYNGTWTVSVDIPDTLHEVGKKVVVWQDVYVAPQTADMTKFKDGLNKLSAGETSKVAKLVVSHHETSEQQ
2 WP_013188882.1   62.4%  35.2%      VEGG--YVPGGHSGTWSVDMTVPTELTQAHKTVVVWEDLYKLPQNVD--TFTTELSKLPTGRT--GAGLIASHHEDNLGA
  consensus/100%                     Vcus..hs.sGasGTWoVshslPspLppstKpVVVWpDlYhhPQssD..pFpstLsKLssGcT..sAtLlsSHHEss.tt
  consensus/90%                      Vcus..hs.sGasGTWoVshslPspLppstKpVVVWpDlYhhPQssD..pFpstLsKLssGcT..sAtLlsSHHEss.tt
  consensus/80%                      Vcus..hs.sGasGTWoVshslPspLppstKpVVVWpDlYhhPQssD..pFpstLsKLssGcT..sAtLlsSHHEss.tt
  consensus/70%                      Vcus..hs.sGasGTWoVshslPspLppstKpVVVWpDlYhhPQssD..pFpstLsKLssGcT..sAtLlsSHHEss.tt

                    cov    pid 7361         .         .         .         4         .         .         .         . 7440
1 WP_004013458.1 100.0% 100.0%      GDGYQWFKVSSNYGSFQVEKTVENGAGLSENVSRQLPKTFKFEYEAKLPAGALLKPGTQPKGEFTLTVDSSNPALAKSPV
2 WP_013188882.1   62.4%  35.2%      GAGTQWLEVSTHFGGFQVNKVVKPAPDLPADVISKIPTTWNFTYVAKLPDGKKAKEGTALEGNFNLTVDPKDPSKAVSQK
  consensus/100%                     GsGhQWhcVSopaGuFQVpKsVcsussLstsV.pplPpTapFpY.AKLPsGthhK.GTt.cGpFsLTVDspsPuhAhS.h
  consensus/90%                      GsGhQWhcVSopaGuFQVpKsVcsussLstsV.pplPpTapFpY.AKLPsGthhK.GTt.cGpFsLTVDspsPuhAhS.h
  consensus/80%                      GsGhQWhcVSopaGuFQVpKsVcsussLstsV.pplPpTapFpY.AKLPsGthhK.GTt.cGpFsLTVDspsPuhAhS.h
  consensus/70%                      GsGhQWhcVSopaGuFQVpKsVcsussLstsV.pplPpTapFpY.AKLPsGthhK.GTt.cGpFsLTVDspsPuhAhS.h

                    cov    pid 7441         :         .         .         .         .         5         .         . 7520
1 WP_004013458.1 100.0% 100.0%      FEGFPVGTEVAITETGVEGTMPTGATMSTTWATADGKAKTKAWSSDRKSDKVTVKIQPRGLLQVKATNHFESTYPKLATL
2 WP_013188882.1   62.4%  35.2%      FDGFPMGTEITITETDAKAQIPTQAKLKVSWKS-------EGWTDAAEGPTKTFTITPAATVSVTALNEFSLVKPTLATV
  consensus/100%                     F-GFPhGTElsITETsscuphPTtAphpsoWto.......cuWosstcusphThpIpPtuhlpVpAhNcFp.shPpLATl
  consensus/90%                      F-GFPhGTElsITETsscuphPTtAphpsoWto.......cuWosstcusphThpIpPtuhlpVpAhNcFp.shPpLATl
  consensus/80%                      F-GFPhGTElsITETsscuphPTtAphpsoWto.......cuWosstcusphThpIpPtuhlpVpAhNcFp.shPpLATl
  consensus/70%                      F-GFPhGTElsITETsscuphPTtAphpsoWto.......cuWosstcusphThpIpPtuhlpVpAhNcFp.shPpLATl

                    cov    pid 7521         .         .         :         .         .         .         .         6 7600
1 WP_004013458.1 100.0% 100.0%      ATTVDGAKMLKPSEATPVLDTVTYSGLVADREYWLLTQLVYTD------DSTPVLGADGQPLARWTKVSAGKDGQGTWVV
2 WP_013188882.1   62.4%  35.2%      ARTADDGKMLKPSEDTAVVDTVTYTNLVQGRDYWLKTELVYVDGTPDPNDNQPVLGADGNPLVKWTKVTAEGD-NSEWVV
  consensus/100%                     ApTsDsuKMLKPSEsTsVlDTVTYosLVtsR-YWLhTpLVYsD......DspPVLGADGpPLs+WTKVoAttD.pupWVV
  consensus/90%                      ApTsDsuKMLKPSEsTsVlDTVTYosLVtsR-YWLhTpLVYsD......DspPVLGADGpPLs+WTKVoAttD.pupWVV
  consensus/80%                      ApTsDsuKMLKPSEsTsVlDTVTYosLVtsR-YWLhTpLVYsD......DspPVLGADGpPLs+WTKVoAttD.pupWVV
  consensus/70%                      ApTsDsuKMLKPSEsTsVlDTVTYosLVtsR-YWLhTpLVYsD......DspPVLGADGpPLs+WTKVoAttD.pupWVV
```

```
                   cov     pid 7601             .         .         .         .         :         .         .         . 7680
1 WP_004013458.1 100.0% 100.0%         DRENPLVVPETTDSQRDLVFFESLFEVPNTPGDAGTKPPDPTDPSNPPIVEHKDPKDPKQVVSSRPKLEMQTVATIGADV
2 WP_013188882.1  62.4%  35.2%         DKDNPMVVPANANPNADVVFFESLYEMTKDGGQP--------KVGDKPIVEHRDNADPKQIITRRPALSLQTTAELS-GV
  consensus/100%                       D+-NPhVVPtssssptDlVFFESLaEhspssGps........csus.PIVEH+DstDPKQllopRPtLphQTsAplu.sV
  consensus/90%                        D+-NPhVVPtssssptDlVFFESLaEhspssGps........csus.PIVEH+DstDPKQllopRPtLphQTsAplu.sV
  consensus/80%                        D+-NPhVVPtssssptDlVFFESLaEhspssGps........csus.PIVEH+DstDPKQllopRPtLphQTsAplu.sV
  consensus/70%                        D+-NPhVVPtssssptDlVFFESLaEhspssGps........csus.PIVEH+DstDPKQllopRPtLphQTsAplu.sV

                   cov     pid 7681             .         7         .         .         .         .         :         . 7760
1 WP_004013458.1 100.0% 100.0%         KTIKPGEAVKITDTVSYNGLKAGGVYTLVGKLVRKTDGEDVSTPVTKTGLVADASGSGKWTMDIPLTAEQTKNLKQGDEL
2 WP_013188882.1  62.4%  35.2%         KSITAGEGATVNDKVTYSGLKAGAEYTLVGTLVKKSDGSIVGSPVTAAGLKAEADGSGSWKMSIPLTAKDTAGLTDGEKL
  consensus/100%                       KoIpsGEusplsDpVoYsGLKAGu.YTLVGpLV+KoDGp.VuoPVTtsGLhA-AsGSGpWpMsIPLTAcpTtsLppG-cL
  consensus/90%                        KoIpsGEusplsDpVoYsGLKAGu.YTLVGpLV+KoDGp.VuoPVTtsGLhA-AsGSGpWpMsIPLTAcpTtsLppG-cL
  consensus/80%                        KoIpsGEusplsDpVoYsGLKAGu.YTLVGpLV+KoDGp.VuoPVTtsGLhA-AsGSGpWpMsIPLTAcpTtsLppG-cL
  consensus/70%                        KoIpsGEusplsDpVoYsGLKAGu.YTLVGpLV+KoDGp.VuoPVTtsGLhA-AsGSGpWpMsIPLTAcpTtsLppG-cL

                   cov     pid 7761             .         .         .         8         .         .         .         . 7840
1 WP_004013458.1 100.0% 100.0%         VVFEKAYLGKMEDAASNP--NLKPILAHEDFKDAGQTVTVVETPDTPPTTPPTTPPYTTPPVSPSTTTPPTTPPNPPVSP
2 WP_013188882.1  62.4%  35.2%         VVFEKAYAGKLPEAGTAPAGTYTPILAHEDLQDASQTVNVVKPTPPPPYTPPVESKTTTPPP-------------PPVKS
  consensus/100%                       VVFEKAYhGKh.-AuosP..shpPILAHEDhpDAuQTVsVVcssssPPhTPPsps.hTTPPs.............PPVps
  consensus/90%                        VVFEKAYhGKh.-AuosP..shpPILAHEDhpDAuQTVsVVcssssPPhTPPsps.hTTPPs.............PPVps
  consensus/80%                        VVFEKAYhGKh.-AuosP..shpPILAHEDhpDAuQTVsVVcssssPPhTPPsps.hTTPPs.............PPVps
  consensus/70%                        VVFEKAYhGKh.-AuosP..shpPILAHEDhpDAuQTVsVVcssssPPhTPPsps.hTTPPs.............PPVps

                   cov     pid 7841             :         .         .         .         .         9         .      ] 7917
1 WP_004013458.1 100.0% 100.0%         SITTPPTT------PPPPAPPVAPATTIPPAQAKMPPTLARTGAQAALVGMLSLAMIAAGGAIGLLAARRKRETTE-
2 WP_013188882.1  62.4%  35.2%         ETTKPPSTPSSTPPDTPPSIPVAPATTVPPMHPKVPPTLARTGAQAAIFGGLSILMIAAGAGLGLLAARRKRESEVK
  consensus/100%                       phTpPPoT......ssPPu.PVAPATTlPPhpsKhPPTLARTGAQAAlhGhLSlhMIAAGuulGLLAARRKREop..
  consensus/90%                        phTpPPoT......ssPPu.PVAPATTlPPhpsKhPPTLARTGAQAAlhGhLSlhMIAAGuulGLLAARRKREop..
  consensus/80%                        phTpPPoT......ssPPu.PVAPATTlPPhpsKhPPTLARTGAQAAlhGhLSlhMIAAGuulGLLAARRKREop..
  consensus/70%                        phTpPPoT......ssPPu.PVAPATTlPPhpsKhPPTLARTGAQAAlhGhLSlhMIAAGuulGLLAARRKREop..
```

MView 1.63, Copyright © 1997-2018 Nigel P. Brown

Figure S1 Pairwise sequence alignment between *M. mulieris* and *M. curtisii* mega-adhesin. Sequences were aligned using Clustal Omega and visualised using MView.

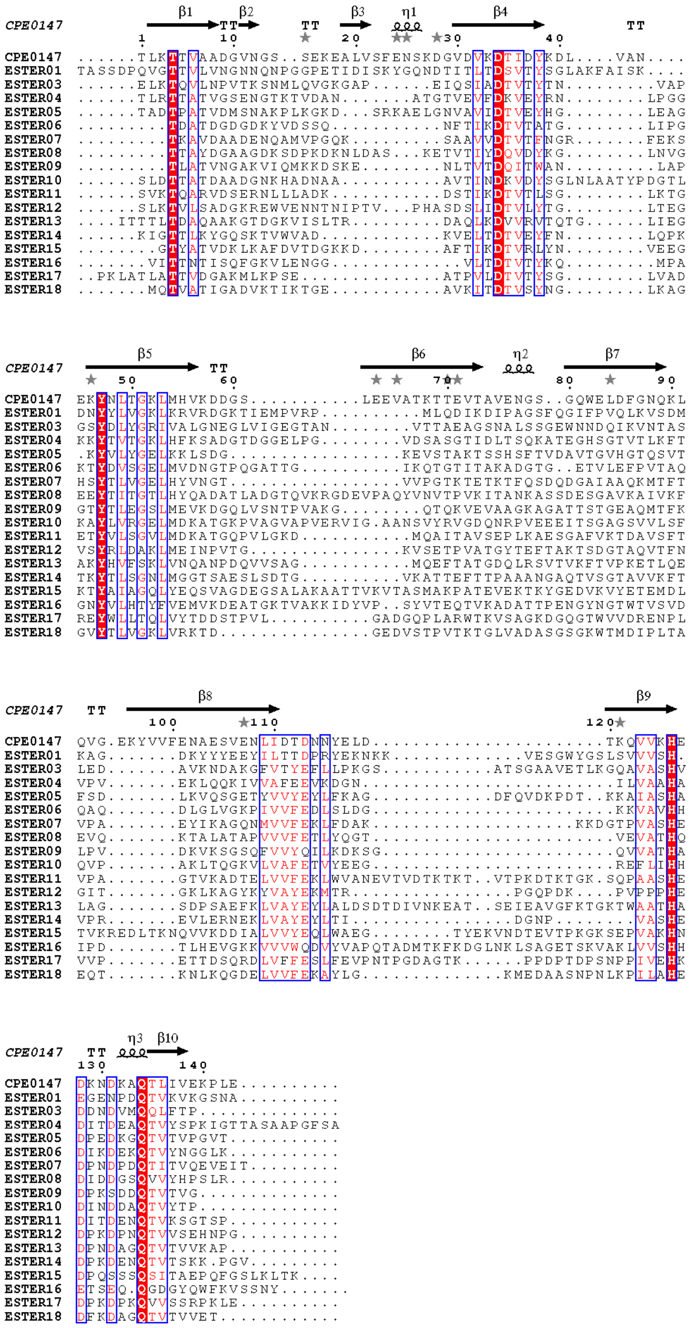

Figure S2 Multiple sequence alignment between ester bond domains within *M. mulieris* mega-adhesin. Sequences were aligned using Clustal Omega and visualised using MView.

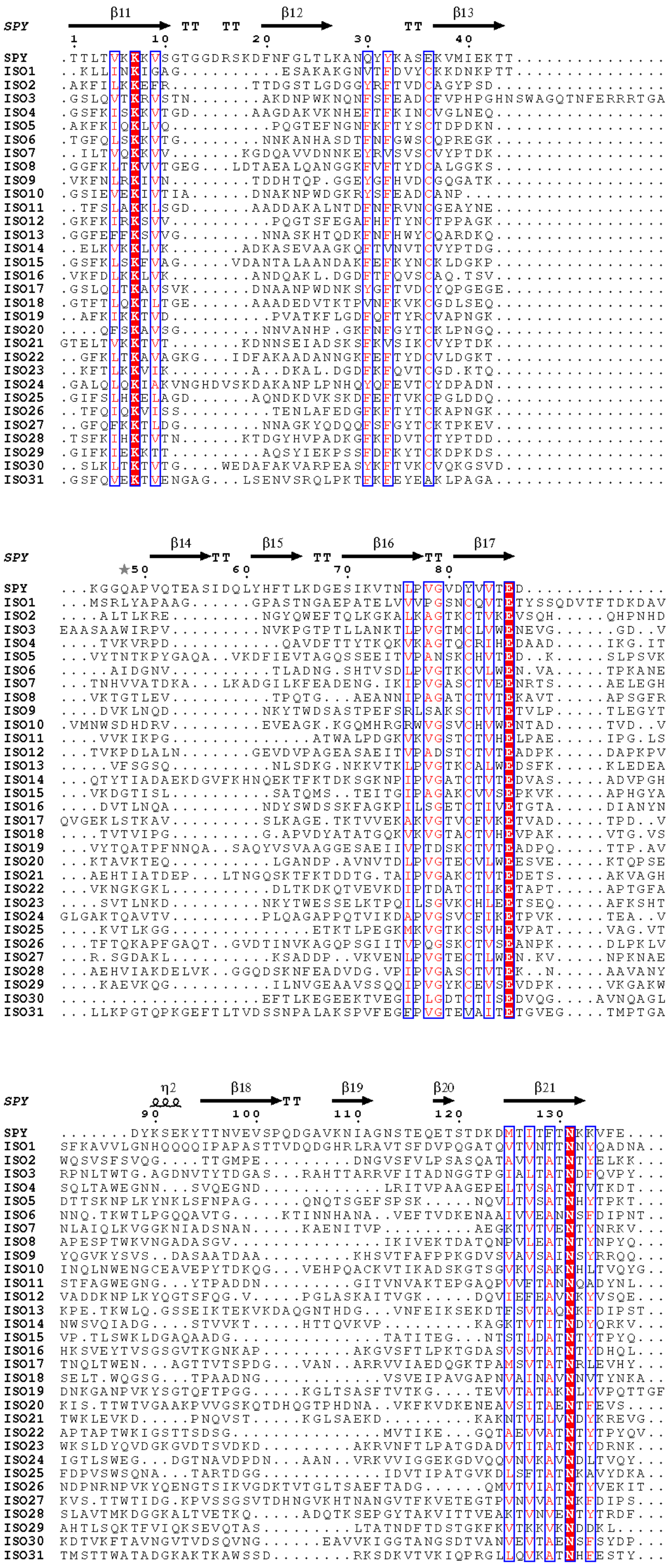

Figure S3 Multiple sequence alignment between isopeptide domains within *M. mulieris* mega-adhesin. Sequences were aligned using Clustal Omega and visualised using MView.

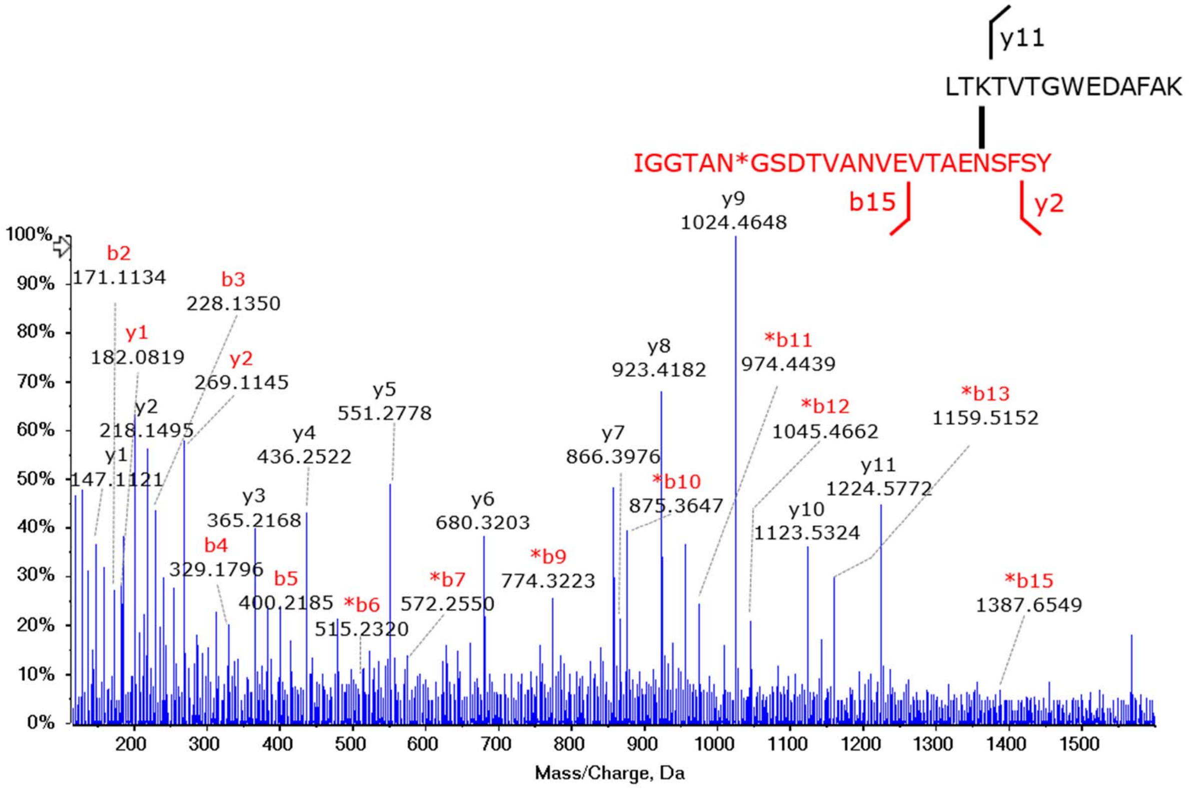

Figure S4 LC-MS/MS analysis of isopeptide bond formation in *M. mulieris* protein construct *46E-47I*. The 4+ ion (m/z 988.97) representing an isopeptide cross-linked peptide from the tryptic digest was collisionally dissociated during LC-MS/MS. Fragment ions labelled in red are derived from the peptide IGGTANGSDTVANVEVTAENSFSY, while those in black are from the peptide LTKTVTGWEDAFAK, with the largest of the respective *b* and *y* series ions shown on the schematic of the cross-linked peptide sequences. The asterisk in the red sequence, and in the red *b* ion series peak labels, indicates the presence of a deamidated asparagine residue at position 6.

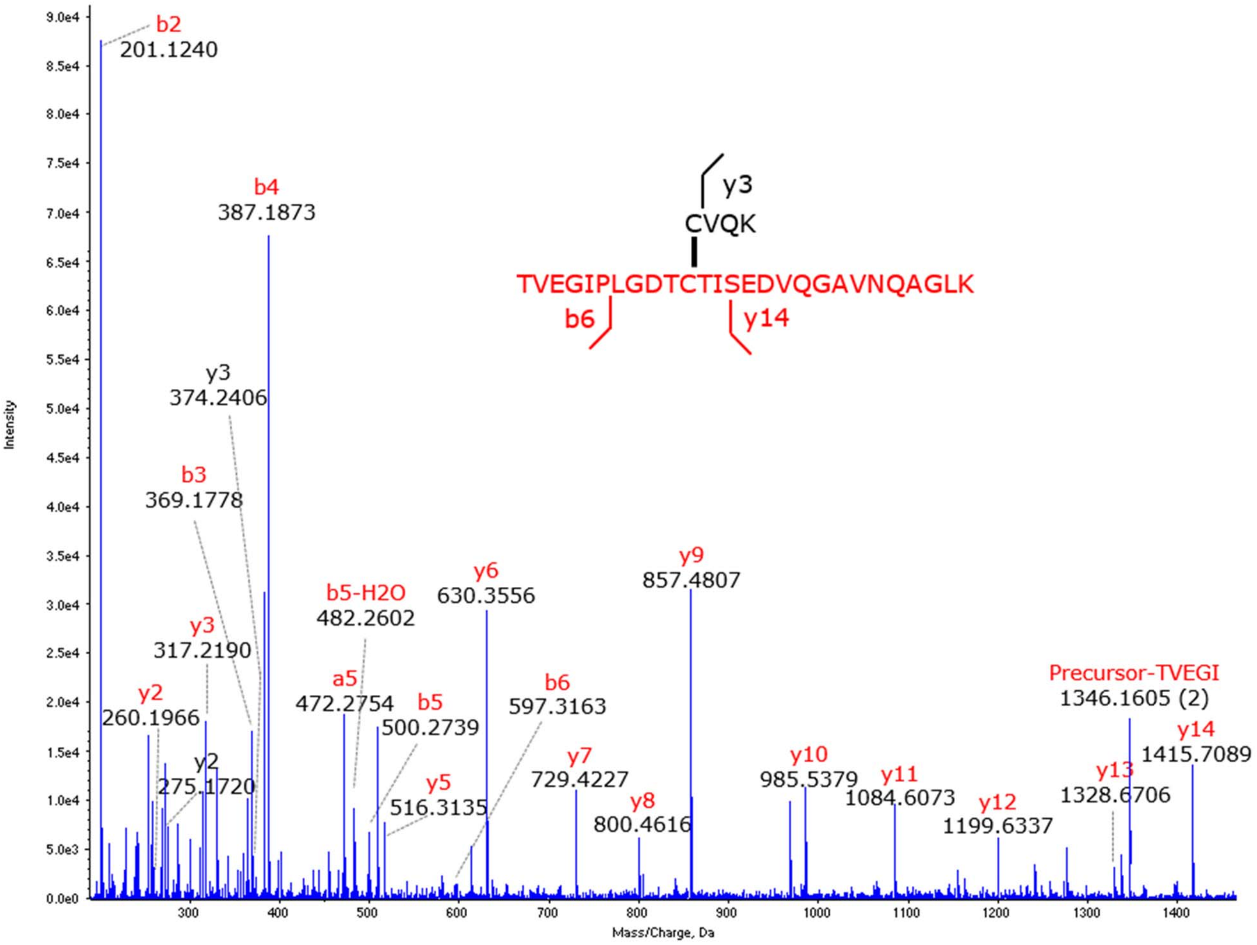

Figure S5 LC-MS/MS analysis of disulfide bond formation in *M. mulieris* protein construct *46E-47I*. The 3+ ion (m/z 1063.87) representing a disulphide cross-linked peptide from the tryptic digest was collisionally dissociated during LC-MS/MS. Fragment ions labelled in red are derived from the peptide TVEGIPLGDTCTISEDVQGAVNQAGLK, while those in black are from the peptide CVQK, with the largest of the respective *b* and *y* series ions shown on the schematic of the cross-linked peptide sequences. Doubly-charged fragment ions indicated in parentheses.

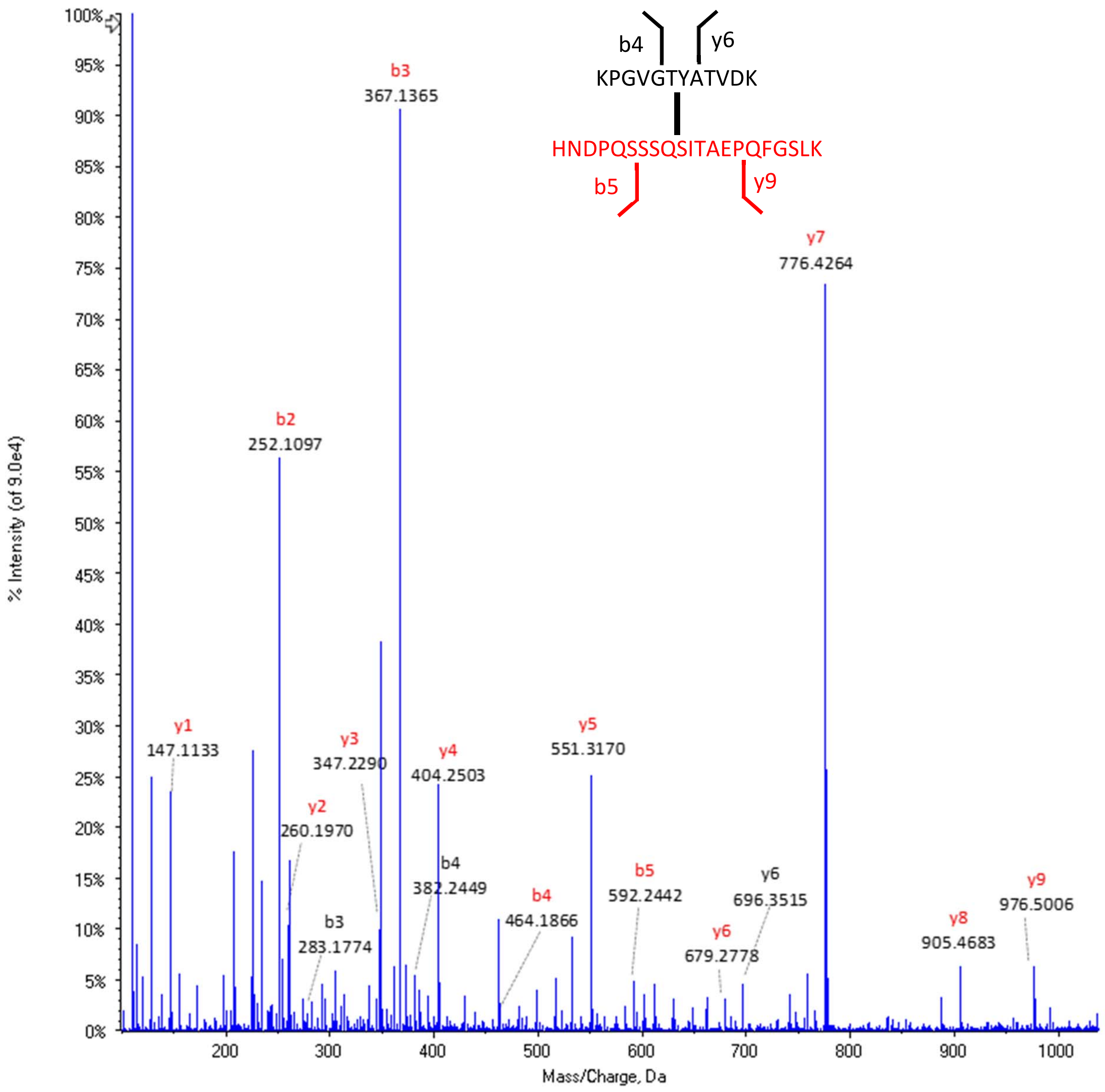

Figure S6 LC-MS/MS analysis of ester bond formation in *M. mulieris* protein construct *46E-47I*. The 3+ ion (m/z 1159.24) representing an ester cross-linked peptide from the tryptic digest was collisionally dissociated during LC-MS/MS. Fragment ions labelled in red are derived from the peptide HNDPQSSSQSITAEPQFGSLK, while those in black are derived from the peptide KPGVGTYATVDK, with the largest of the respective *b* and *y* series ions shown on the schematic of the cross-linked peptide.

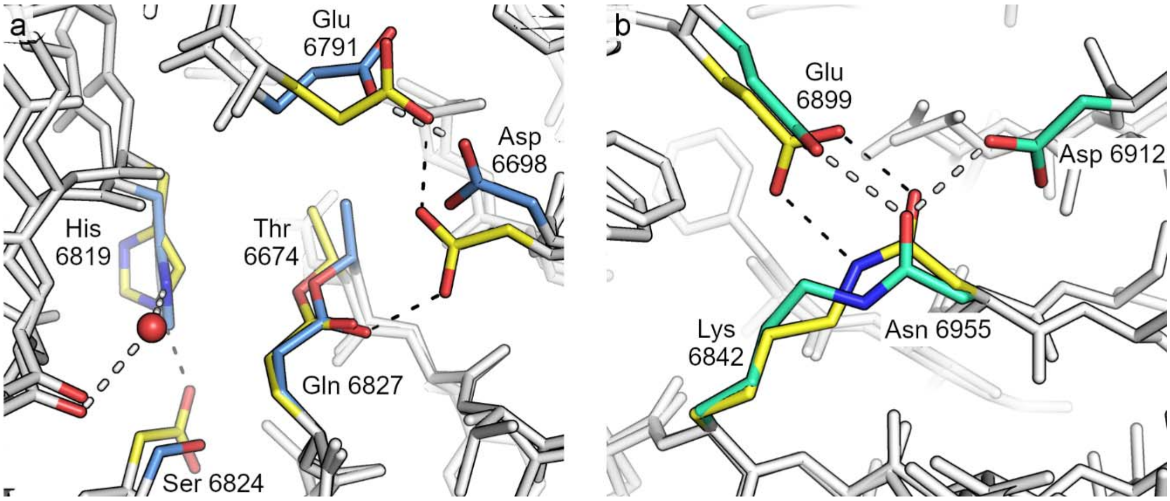

Figure S7 Comparison of *M. mulieris* domains *46E-47I* crosslinks and exemplar crystal structures. **a)** Overlay of ester bond crosslinks in domain *46E* (*M. mulieris*) and domain 1 of the Cpe0147 adhesin (PDB 4NI6). **b)** Overlay of isopeptide bond crosslinks in domain *47I* (*M. mulieris*) and domain 2 of the Spy0128 pilin protein (PDB 3B2M). Hydrogen bonds are shown as dashed lines; sequences are numbered for the *46E-47I M. mulieris* protein. An integral water molecule is highlighted as a red sphere.

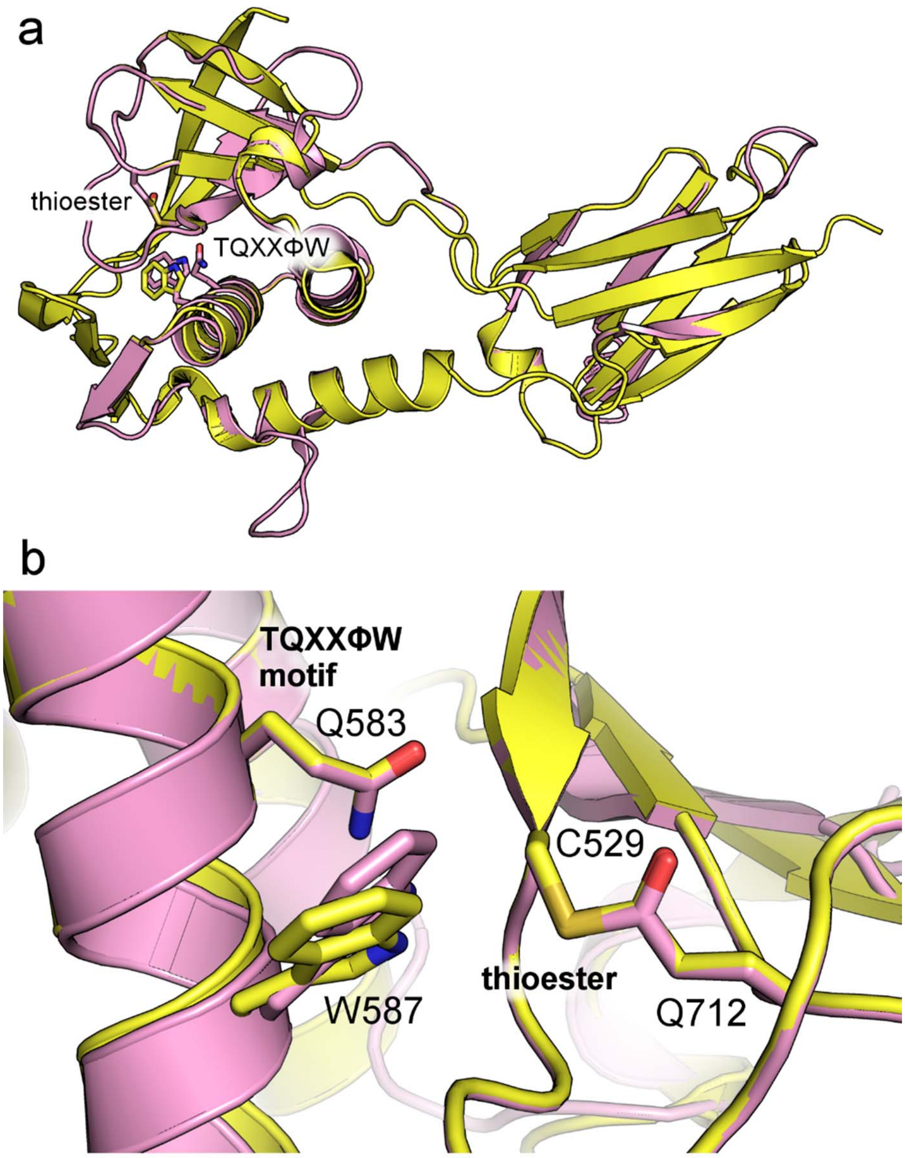

Figure S8 Structure prediction of the N-terminal thioester (TED) domain of *M. mulieris* adhesin. **a)** The predicted *M. mulieris* model (pink) overlaid with the X-ray crystal structure of the class II TED from vancomycin-resistant *Staphylococcus aureus*, SaTIE (yellow; PDB ID 6FX6). **b)** Zoomed view highlighting the predicted intramolecular thioester bond and the conserved TQXXϕW motif. The structure was predicted using the AlphaFold2 server.

```
Reference sequence (1): WP_004013458.1
Identities normalised by aligned length.
Colored by: identity
```

```
                    cov    pid    1 [        .         .         .         .         :         .         .         . 80
1 WP_004013458.1 100.0% 100.0%      MGDKTRGTQGRMPVSGLIKVGKGKRRRTTRNWGAAFVAASVAVAWTVGGLVMPSMAAEPAVIGKVHPEVNISGGGTANVS
2 WP_101929469.1  37.0%  15.7%      --------------------------------------------------------------------------------
  consensus/100%                    ................................................................................
  consensus/90%                     ................................................................................
  consensus/80%                     ................................................................................
  consensus/70%                     ................................................................................

                    cov    pid   81          .         1         .         .         .         .         :         . 160
1 WP_004013458.1 100.0% 100.0%      LAGQNTNLYYAALSTNLFTTIDRVYAQKMYGKTEQLGDFTAQYLPRACFVRDDRIISNEPLGNFIRMDEGGPSYLGRRYG
2 WP_101929469.1  37.0%  15.7%      --------------------------------------------------------------------------------
  consensus/100%                    ................................................................................
  consensus/90%                     ................................................................................
  consensus/80%                     ................................................................................
  consensus/70%                     ................................................................................

                    cov    pid  161          .         .         .         2         .         .         .         . 240
1 WP_004013458.1 100.0% 100.0%      AWVLYPNGSKSFSVDSSKSRSADRSVDSPSDVDDADDTEDTLKLTLDKLSFDDVVGDAAAAADKEFSDSQLPNEKTGKEQ
2 WP_101929469.1  37.0%  15.7%      --------------------------------------------------------------------------------
  consensus/100%                    ................................................................................
  consensus/90%                     ................................................................................
  consensus/80%                     ................................................................................
  consensus/70%                     ................................................................................

                    cov    pid  241          :         .         .         .         .         3         .         . 320
1 WP_004013458.1 100.0% 100.0%      KQTAESPVENPKESLASKEGGETQAKDVPPNGSESSSNSESAPELAKPTPAETSTAETEPKETKKPSETKPSSQFVEEKG
2 WP_101929469.1  37.0%  15.7%      --------------------------------------------------------------------------------
  consensus/100%                    ................................................................................
  consensus/90%                     ................................................................................
  consensus/80%                     ................................................................................
  consensus/70%                     ................................................................................

                    cov    pid  321          .         .         :         .         .         .         .         4 400
1 WP_004013458.1 100.0% 100.0%      HKAGNLDKESKSLEDRSAVSKQGKDSAENSNKADKNQGKDRSAVGQDRAVLFSAFQLKTKELKVKFDPQKSKDKAIEDVN
2 WP_101929469.1  37.0%  15.7%      --------------------------------------------------------------------------------
  consensus/100%                    ................................................................................
  consensus/90%                     ................................................................................
  consensus/80%                     ................................................................................
  consensus/70%                     ................................................................................

                    cov    pid  401          .         .         .         .         :         .         .         . 480
1 WP_004013458.1 100.0% 100.0%      KQVDEIQKDALQQLDEFVTKNRVPQDIADKFRTKLNIQFENMRRQAVEFLEKKYAKGAIGADEWRNCQQSGPIVPDGSLV
2 WP_101929469.1  37.0%  15.7%      --------------------------------------------------------------------------------
  consensus/100%                    ................................................................................
  consensus/90%                     ................................................................................
```

```
  consensus/80%                        ................................................................................
  consensus/70%                        ................................................................................

                    cov    pid  481           .         5         .         .         .         .         :         . 560
1 WP_004013458.1 100.0% 100.0%     LVNEARLDNHNKKAWYLGVKQNDYTISRLLQYASEGSGNIKADGADSFCVEPLKGLQPGAHNIYTHPSTLEWAQNHPERA
2 WP_101929469.1  37.0%  15.7%     --------------------------------------------------------------------------------
  consensus/100%                       ................................................................................
  consensus/90%                        ................................................................................
  consensus/80%                        ................................................................................
  consensus/70%                        ................................................................................

                    cov    pid  561           .         .         .         6         .         .         .         . 640
1 WP_004013458.1 100.0% 100.0%     ELVKALAWHYKQFAQPKEYAKYQNAIWSVVMGLPSMVGSWDGMKRFWVSEPGSSAGLISAAESAYKTRPSVVQALKSMHL
2 WP_101929469.1  37.0%  15.7%     --------------------------------------------------------------------------------
  consensus/100%                       ................................................................................
  consensus/90%                        ................................................................................
  consensus/80%                        ................................................................................
  consensus/70%                        ................................................................................

                    cov    pid  641           :         .         .         .         .         7         .         . 720
1 WP_004013458.1 100.0% 100.0%     DQISVDTAPDGKSKVIFLQLNDYDKSSEIRRAIGNSVYLRVTGATTPEGKPVDRVSLEEAERGVHFLVKNQDQFSVAFAG
2 WP_101929469.1  37.0%  15.7%     --------------------------------------------------------------------------------
  consensus/100%                       ................................................................................
  consensus/90%                        ................................................................................
  consensus/80%                        ................................................................................
  consensus/70%                        ................................................................................

                    cov    pid  721           .         .         :         .         .         .         .         8 800
1 WP_004013458.1 100.0% 100.0%     TLNNAQDAYFLDKPSHDTQAQVTVLSKKITVSGSLKIQWGTTASSDPQVGTTVLVNGNNQNPGGPETVDISKYGQNDTIT
2 WP_101929469.1  37.0%  15.7%     --------------------------------------------------------------------------------
  consensus/100%                       ................................................................................
  consensus/90%                        ................................................................................
  consensus/80%                        ................................................................................
  consensus/70%                        ................................................................................

                    cov    pid  801           .         .         .         .         :         .         .         . 880
1 WP_004013458.1 100.0% 100.0%     LTDSVTYSGLAKFAISKDNYYLVGKLKRVRDGKTIEMPVRPMLQDIKDIPAGSFQGIFPVQLKVSDMKAGDKYYYEEYIL
2 WP_101929469.1  37.0%  15.7%     --------------------------------------------------------------------------------
  consensus/100%                       ................................................................................
  consensus/90%                        ................................................................................
  consensus/80%                        ................................................................................
  consensus/70%                        ................................................................................

                    cov    pid  881           .         9         .         .         .         .         :         . 960
1 WP_004013458.1 100.0% 100.0%     TTDPRYEKNKKVESGWYGPLSVVVSHEGENPDQTVKVKGSNAKLLINKIGAGESAKAKGNVTFDVYCKKDNKPTTMSRLY
2 WP_101929469.1  37.0%  15.7%     --------------------------------------------------------------------------------
  consensus/100%                       ................................................................................
  consensus/90%                        ................................................................................
  consensus/80%                        ................................................................................
  consensus/70%                        ................................................................................

                    cov    pid  961           .         .         .         0         .         .         .         . 1040
1 WP_004013458.1 100.0% 100.0%     APAAGGPASTNGAEPATELVVVPGSNCQVTETYSSQDVTFTDKDAVSFKAVVLGNHQQQQIPAPASTTVDQDGHRLRAVT
2 WP_101929469.1  37.0%  15.7%     --------------------------------------------------------------------------------
  consensus/100%                       ................................................................................
  consensus/90%                        ................................................................................
  consensus/80%                        ................................................................................
  consensus/70%                        ................................................................................

                    cov    pid 1041           :         .         .         .         .         1         .         . 1120
1 WP_004013458.1 100.0% 100.0%     SFDVPQGATQVTVNTTNNYQADNAAKFILKKEFRTTDGSTLGDGGYRFTVDCAGYPSDALTLKRENGYQWEFTQLKGKAL
2 WP_101929469.1  37.0%  15.7%     --------------------------------------------------------------------------------
  consensus/100%                       ................................................................................
  consensus/90%                        ................................................................................
  consensus/80%                        ................................................................................
  consensus/70%                        ................................................................................

                    cov    pid 1121           .         .         :         .         .         .         .         2 1200
1 WP_004013458.1 100.0% 100.0%     KAGTKCTVKEVSQHQHPNHDWQSVSFSVQGTTGMPEDNGVSFVLPSASQATAVVTATNTYELKKGSLQVTKRVSTNAKDN
2 WP_101929469.1  37.0%  15.7%     --------------------------------------------------------------------------------
  consensus/100%                       ................................................................................
  consensus/90%                        ................................................................................
  consensus/80%                        ................................................................................
  consensus/70%                        ................................................................................

                    cov    pid 1201           .         .         .         .         :         .         .         . 1280
1 WP_004013458.1 100.0% 100.0%     PWKNQNFSFEADCFVPHPGHNSWAGQTNFERRRTGAEAASAAWIRPVNVKPGTPTLLANKTLPVGTMCLVWENEVGGDVR
2 WP_101929469.1  37.0%  15.7%     --------------------------------------------------------------------------------
  consensus/100%                       ................................................................................
  consensus/90%                        ................................................................................
  consensus/80%                        ................................................................................
  consensus/70%                        ................................................................................
```

```
                   cov     pid 1281          .         3         .         .         .         .         :         . 1360
1 WP_004013458.1 100.0% 100.0%      PNLTWTGAGDNVTYTDGASRAHTTARRVFITADNGGTPGIALTATNDFQVPYGSFKISKKVTGDAAGDAKVKNHEFTFKI
2 WP_101929469.1  37.0%  15.7%      --------------------------------------------------------------------------------
  consensus/100%                    ................................................................................
  consensus/90%                     ................................................................................
  consensus/80%                     ................................................................................
  consensus/70%                     ................................................................................

                   cov     pid 1361          .         .         .         4         .         .         .         . 1440
1 WP_004013458.1 100.0% 100.0%      NCVGLNEQTVKVRPDQAVDFTTYTKQKVKAGTQCRIHEDAADIKGITSQLTAWEGNNSVQEGNDLKITVPAAGEPELTVS
2 WP_101929469.1  37.0%  15.7%      --------------------------------------------------------------------------------
  consensus/100%                    ................................................................................
  consensus/90%                     ................................................................................
  consensus/80%                     ................................................................................
  consensus/70%                     ................................................................................

                   cov     pid 1441          :         .         .         .         .         5         .         . 1520
1 WP_004013458.1 100.0% 100.0%      ATNTVTKDTAKFKIQKLVQPQGTEFNGNFKFTYSCTDPDKNVYTNTKPYGAQAVKDFIEVTAGQSSEEITVPANSKCHVT
2 WP_101929469.1  37.0%  15.7%      --------------------------------------------------------------------------------
  consensus/100%                    ................................................................................
  consensus/90%                     ................................................................................
  consensus/80%                     ................................................................................
  consensus/70%                     ................................................................................

                   cov     pid 1521          .         .         :         .         .         .         .         6 1600
1 WP_004013458.1 100.0% 100.0%      EDKSLPSVKDTTSKNPLKYNKLSFNPAGQNQTSGEFSPSKNQVLTVSATNHYTPKTTGFQLSKKVTGNNKANHASDTFNF
2 WP_101929469.1  37.0%  15.7%      --------------------------------------------------------------------------------
  consensus/100%                    ................................................................................
  consensus/90%                     ................................................................................
  consensus/80%                     ................................................................................
  consensus/70%                     ................................................................................

                   cov     pid 1601          .         .         .         .         :         .         .         . 1680
1 WP_004013458.1 100.0% 100.0%      GWSCQPREGKAIDGNVTLADNGSHTVSDLPVGTKCVLWENVATPKANENNQTKWTLPGQQAVTGKTINNHANAVEFTVDK
2 WP_101929469.1  37.0%  15.7%      --------------------------------------------------------------------------------
  consensus/100%                    ................................................................................
  consensus/90%                     ................................................................................
  consensus/80%                     ................................................................................
  consensus/70%                     ................................................................................

                   cov     pid 1681          .         7         .         .         .         .         :         . 1760
1 WP_004013458.1 100.0% 100.0%      ENAAIVVEANNSFDIPNTSLTVQKKVVKGDQAVVDNNKEYRVSVSCVYPTDKTNHVVATDKALKADGILKFEADENGIKI
2 WP_101929469.1  37.0%  15.7%      --------------------------------------------------------------------------------
  consensus/100%                    ................................................................................
  consensus/90%                     ................................................................................
  consensus/80%                     ................................................................................
  consensus/70%                     ................................................................................

                   cov     pid 1761          .         .         .         8         .         .         .         . 1840
1 WP_004013458.1 100.0% 100.0%      PVGASCTVEENRTSAELEGHNLAIQLKVGGKNIADSNANKAENITVPAEGKTVTVENTYNRKLGGFKLTKVVTGEGLDTA
2 WP_101929469.1  37.0%  15.7%      --------------------------------------------------------------------------------
  consensus/100%                    ................................................................................
  consensus/90%                     ................................................................................
  consensus/80%                     ................................................................................
  consensus/70%                     ................................................................................

                   cov     pid 1841          :         .         .         .         .         9         .         . 1920
1 WP_004013458.1 100.0% 100.0%      EALQANGGKFVFTYDCALGGKSVKTGTLEVTPQTGAEANNIPAGATCTLTEKAVTAPSGFRAPERPTWKVNGADASGVIK
2 WP_101929469.1  37.0%  15.7%      --------------------------------------------------------------------------------
  consensus/100%                    ................................................................................
  consensus/90%                     ................................................................................
  consensus/80%                     ................................................................................
  consensus/70%                     ................................................................................

                   cov     pid 1921          .         .         :         .         .         .         .         0 2000
1 WP_004013458.1 100.0% 100.0%      IVEKTDATQNPVLEATNTYTPYQVKFNLRKIVNTDDHTQPGGEYGFHVDCGQGATKDVKLNQDNKYTWDSASTPEFSRLS
2 WP_101929469.1  37.0%  15.7%      --------------------------------------------------------------------------------
  consensus/100%                    ................................................................................
  consensus/90%                     ................................................................................
  consensus/80%                     ................................................................................
  consensus/70%                     ................................................................................

                   cov     pid 2001          .         .         .         .         :         .         .         . 2080
1 WP_004013458.1 100.0% 100.0%      AKSCTVTETVLPTLEGYTYQGVKYSVSDASAATDAAKRSVTFAFPPKGDVSVAVSATNSYRRQQGSIEVEKIVTIADNAK
2 WP_101929469.1  37.0%  15.7%      --------------------------------------------------------------------------------
  consensus/100%                    ................................................................................
  consensus/90%                     ................................................................................
  consensus/80%                     ................................................................................
  consensus/70%                     ................................................................................

                   cov     pid 2081          .         1         .         .         .         .         :         . 2160
```

```
1 WP_004013458.1 100.0% 100.0%      NPWDGKRYSFEADCANPVMNWSDHDRVEVEAGKKGQMHRGRWVGSVCHVWENTADTVDVINQLNWENGCEAVEPYTDKQG
2 WP_101929469.1  37.0%  15.7%      --------------------------------------------------------------------------------
  consensus/100%                    ................................................................................
  consensus/90%                     ................................................................................
  consensus/80%                     ................................................................................
  consensus/70%                     ................................................................................

                      cov    pid 2161        .         .         .         2         .         .         .         . 2240
1 WP_004013458.1 100.0% 100.0%      VEHPQACKVTIKADSKGTSGVKVSAKNHLTVQYGTFSLAKKLSGDAADDAKALNTDFNFRVNCGEAYNEVVKIKPGATWA
2 WP_101929469.1  37.0%  15.7%      --------------------------------------------------------------------------------
  consensus/100%                    ................................................................................
  consensus/90%                     ................................................................................
  consensus/80%                     ................................................................................
  consensus/70%                     ................................................................................

                      cov    pid 2241        :         .         .         .         .         3         .         . 2320
1 WP_004013458.1 100.0% 100.0%      LPDGKVKVGSTCTVHELPAEIPGLSSTFAGWEGNGYTPADDNGITVNVAKTEPGAQPVVFTANNQADYNLGKFKIRKSVV
2 WP_101929469.1  37.0%  15.7%      --------------------------------------------------------------------------------
  consensus/100%                    ................................................................................
  consensus/90%                     ................................................................................
  consensus/80%                     ................................................................................
  consensus/70%                     ................................................................................

                      cov    pid 2321        .         .         :         .         .         .         .         4 2400
1 WP_004013458.1 100.0% 100.0%      PQGTSFEGTFHFTYNCTPPAGKTVKPDLALNGEVDVPAGEASAEITVPADSTCTVTEADPKDAPKPVVADDKNPLKYQGT
2 WP_101929469.1  37.0%  15.7%      --------------------------------------------------------------------------------
  consensus/100%                    ................................................................................
  consensus/90%                     ................................................................................
  consensus/80%                     ................................................................................
  consensus/70%                     ................................................................................

                      cov    pid 2401        .         .         .         .         :         .         .         . 2480
1 WP_004013458.1 100.0% 100.0%      SFQGVPGLASKAITVGKDQVIEFEAVNKYVSQEGGFEFFKSVVGNNASKHTQDKFNFHWYCQARDKQVFSGSQNLSDKGN
2 WP_101929469.1  37.0%  15.7%      --------------------------------------------------------------------------------
  consensus/100%                    ................................................................................
  consensus/90%                     ................................................................................
  consensus/80%                     ................................................................................
  consensus/70%                     ................................................................................

                      cov    pid 2481        .         5         .         .         .         .         :         . 2560
1 WP_004013458.1 100.0% 100.0%      KKVTKLPVGTKCALWEDSFKKLEDEAKPETKWLQGSSEIKTEKVKDAQGNTHDGVNFEIKSEKDTFSVTAQNKFDIPSTE
2 WP_101929469.1  37.0%  15.7%      --------------------------------------------------------------------------------
  consensus/100%                    ................................................................................
  consensus/90%                     ................................................................................
  consensus/80%                     ................................................................................
  consensus/70%                     ................................................................................

                      cov    pid 2561        .         .         .         6         .         .         .         . 2640
1 WP_004013458.1 100.0% 100.0%      LKVKKLVKADKASKVAAGKQFTVNVTCVYPTDGQTYTIADAEKDGVFKHNQEKTFKTDKSGKNPIPVGAICTVTEDVASA
2 WP_101929469.1  37.0%  15.7%      --------------------------------------------------------------------------------
  consensus/100%                    ................................................................................
  consensus/90%                     ................................................................................
  consensus/80%                     ................................................................................
  consensus/70%                     ................................................................................

                      cov    pid 2641        :         .         .         .         .         7         .         . 2720
1 WP_004013458.1 100.0% 100.0%      DVPGHNWSVQIADGSTVVKTHTTRVKVPKAGKTVTITNDYERKVGSFKLSKFVAGVDVNTALAANDAKFEFKYNCKLDGK
2 WP_101929469.1  37.0%  15.7%      ----------------------------------MINSQTAKLLRRW----IVAGLAAALAL---------AWTITTLVA
  consensus/100%                    ..................................hIsspht+hltpa....hVAGlssshAL.........tashph.st
  consensus/90%                     ..................................hIsspht+hltpa....hVAGlssshAL.........tashph.st
  consensus/80%                     ..................................hIsspht+hltpa....hVAGlssshAL.........tashph.st
  consensus/70%                     ..................................hIsspht+hltpa....hVAGlssshAL.........tashph.st

                      cov    pid 2721        .         .         :         .         .         .         .         8 2800
1 WP_004013458.1 100.0% 100.0%      PVKDGTISLSATQMSTEITGIPAGAKCVVSEP----KVKAPHGYAVPTLSWKLDGAQAADGTA-TITEGNTSTLDATNTY
2 WP_101929469.1  37.0%  15.7%      PSQA---STDENVTVDAVSGTPTSLNLKYPDSDNAGRFPYPGAY-TAVESWRNNNPQVYQEWQQRINDSYGGSADTPVEY
  consensus/100%                    Psps...Shsts.hsstloGhPsuhphhhs-s....+h.hPtuY.sss.SW+.sssQshptht.pIs-u.suohDssspY
  consensus/90%                     Psps...Shsts.hsstloGhPsuhphhhs-s....+h.hPtuY.sss.SW+.sssQshptht.pIs-u.suohDssspY
  consensus/80%                     Psps...Shsts.hsstloGhPsuhphhhs-s....+h.hPtuY.sss.SW+.sssQshptht.pIs-u.suohDssspY
  consensus/70%                     Psps...Shsts.hsstloGhPsuhphhhs-s....+h.hPtuY.sss.SW+.sssQshptht.pIs-u.suohDssspY

                      cov    pid 2801        .         .         .         .         :         .         .         . 2880
1 WP_004013458.1 100.0% 100.0%      TPYQVKFDLKKLVKANDQAKLDGDFTFQVSCAQTSVDVTLNQANDYSWDSSKF--------AGKPILSGETCTIVETGTA
2 WP_101929469.1  37.0%  15.7%      RYMTV---------EEPFRDVQG-------------------QWRYKWVDPLGYFLPNAGPGKNQVITESACIDPNVGQD
  consensus/100%                    p.hpV.........tps.tclpG...................t.cYpWssshh........utp.llotpsCh.spsGps
  consensus/90%                     p.hpV.........tps.tclpG...................t.cYpWssshh........utp.llotpsCh.spsGps
  consensus/80%                     p.hpV.........tps.tclpG...................t.cYpWssshh........utp.llotpsCh.spsGps
  consensus/70%                     p.hpV.........tps.tclpG...................t.cYpWssshh........utp.llotpsCh.spsGps

                      cov    pid 2881        .         9         .         .         .         .         :         . 2960
1 WP_004013458.1 100.0% 100.0%      DIANYNHKSVEYTVSGSGVTKGNKAPAKGVSFTLPKTGDAS---VSVTATNTYDHQLGSLQLTKAVSVKDNAANPWDNKS
2 WP_101929469.1  37.0%  15.7%      PIYTTYVKNLSKNAVQ---TRITLDPQKAIEYQKKAGKNYSPEQQGMFKALAYVNTGGDPRLNDFVNYDDATL-KQKIAE
```

```
  consensus/100%                    sIhsh.hKslphssst...T+hshsPtKulpaph.tstshS....uhhts.sYspphGs.pLschVshcDssh...c.tp
  consensus/90%                     sIhsh.hKslphssst...T+hshsPtKulpaph.tstshS....uhhts.sYspphGs.pLschVshcDssh...c.tp
  consensus/80%                     sIhsh.hKslphssst...T+hshsPtKulpaph.tstshS....uhhts.sYspphGs.pLschVshcDssh...c.tp
  consensus/70%                     sIhsh.hKslphssst...T+hshsPtKulpaph.tstshS....uhhts.sYspphGs.pLschVshcDssh...c.tp

                 cov    pid 2961         .         .         .         0         .         .         .         . 3040
1 WP_004013458.1 100.0% 100.0%      YGFTVDCYQPGEGEQVG-EKLSTKAVSLKAGETKTVVEKAKVGTVCFVKETVADTPDVTNQLTWENAGTTVTSPDGVANA
2 WP_101929469.1  37.0%  15.7%      YPAIGDFYNANMALIEAQRDAVAKIKSLKPNQ---------------------KYQVLQGLAWAINR-----------
  consensus/100%                    YshhsDhYpss.u...u.cchssKhhSLKssp.....................p.pVhptLsWt.st...........
  consensus/90%                     YshhsDhYpss.u...u.cchssKhhSLKssp.....................p.pVhptLsWt.st...........
  consensus/80%                     YshhsDhYpss.u...u.cchssKhhSLKssp.....................p.pVhptLsWt.st...........
  consensus/70%                     YshhsDhYpss.u...u.cchssKhhSLKssp.....................p.pVhptLsWt.st...........

                 cov    pid 3041         :         .         .         .         .         1         .         . 3120
1 WP_004013458.1 100.0% 100.0%      RRVVIAEDQGKTPAMSVTATNRLEVHYGTFTLQKTLTGEAAADEDVTKTPVNFKVKCGDLSEQTVTVIPGGAPVDYATAT
2 WP_101929469.1  37.0%  15.7%      ------------------------------------------------------------------------------GN
  consensus/100%                    ..............................................................................us
  consensus/90%                     ..............................................................................us
  consensus/80%                     ..............................................................................us
  consensus/70%                     ..............................................................................us

                 cov    pid 3121         .         .         :         .         .         .         .         2 3200
1 WP_004013458.1 100.0% 100.0%      GQKVKVGTACTVHEVPAKV-TGVSSELTWQGSGTPAADNGVSVEIPAVGAPNVAIKAVNNVTYNKAAFKIKKTVDPVATK
2 WP_101929469.1  37.0%  15.7%      TDWLAIGDNCTYIDQYKTANCQVNKELTQLA-------HDVYADLKVMASSEL--KMYEAYAKYKEQFKNKR--DEYE-K
  consensus/100%                    sphltlGssCThh-..tps.stVspELT..u.......psV.s-l.shuuspl..Khhpshsh.KttFK.K+..D.ht.K
  consensus/90%                     sphltlGssCThh-..tps.stVspELT..u.......psV.s-l.shuuspl..Khhpshsh.KttFK.K+..D.ht.K
  consensus/80%                     sphltlGssCThh-..tps.stVspELT..u.......psV.s-l.shuuspl..Khhpshsh.KttFK.K+..D.ht.K
  consensus/70%                     sphltlGssCThh-..tps.stVspELT..u.......psV.s-l.shuuspl..Khhpshsh.KttFK.K+..D.ht.K

                 cov    pid 3201         .         .         .         .         :         .         .         . 3280
1 WP_004013458.1 100.0% 100.0%      FLGDFQ-FTYRCVAPNGKVYTQATPFNNQASAQYVSVAA-G----------EESAEII-----------------VPTDS
2 WP_101929469.1  37.0%  15.7%      ALKKWQEETQRQVEANEKLYQTNKELLSSI-KIYVDKTDDGKKLAIKLHGSSEALALLQQSKTNIDVNLTVNGKTVPVNG
  consensus/100%                    hLtcaQ..T.RpVtsNtKlYppsp.h.sph.t.YVshss.G..........pEuhtll.................VPssu
  consensus/90%                     hLtcaQ..T.RpVtsNtKlYppsp.h.sph.t.YVshss.G..........pEuhtll.................VPssu
  consensus/80%                     hLtcaQ..T.RpVtsNtKlYppsp.h.sph.t.YVshss.G..........pEuhtll.................VPssu
  consensus/70%                     hLtcaQ..T.RpVtsNtKlYppsp.h.sph.t.YVshss.G..........pEuhtll.................VPssu

                 cov    pid 3281         .         3         .         .         .         .         :         . 3360
1 WP_004013458.1 100.0% 100.0%      KCTVTEADPQTTPAVDNKGANPVKYSGTQFTPGGKGLTSASFTVT---KGTEVVTATAKNLYVPQTTGFQFSKAVSGNNV
2 WP_101929469.1  37.0%  15.7%      VSVAAAASPEGIPV-------PVDSTGLGCDQKLAGTVTASFSGEIEISGTYIFD--GRNKLQRQVVLQKYKVAVSGN--
  consensus/100%                    hsssstAsPpshPs.......PVc.oGhths.thtGhsoASFosp...pGT.lhs..u+Nhh..Qssh.paphAVSGN..
  consensus/90%                     hsssstAsPpshPs.......PVc.oGhths.thtGhsoASFosp...pGT.lhs..u+Nhh..Qssh.paphAVSGN..
  consensus/80%                     hsssstAsPpshPs.......PVc.oGhths.thtGhsoASFosp...pGT.lhs..u+Nhh..Qssh.paphAVSGN..
  consensus/70%                     hsssstAsPpshPs.......PVc.oGhths.thtGhsoASFosp...pGT.lhs..u+Nhh..Qssh.paphAVSGN..

                 cov    pid 3361         .         .         .         4         .         .         .         . 3440
1 WP_004013458.1 100.0% 100.0%      ANHPGKFNFGYTCKLPNGQKTAVKTEQLGANDPAV-----------------NVTDLPVGTECVLWEESVEKTQPSEKI
2 WP_101929469.1  37.0%  15.7%      --TSHDFSFAGTCPVAPERKIGTTLE--GSDGAKVIDTAGKKRTEKVTLVDKVKFENLKGGQNYVIKGELVDGNGN----
  consensus/100%                    ..pstcFsFuhTC.lsstpKhusphE..GussstV.................phpsL.sGpphVlhtE.V-tsts....
  consensus/90%                     ..pstcFsFuhTC.lsstpKhusphE..GussstV.................phpsL.sGpphVlhtE.V-tsts....
  consensus/80%                     ..pstcFsFuhTC.lsstpKhusphE..GussstV.................phpsL.sGpphVlhtE.V-tsts....
  consensus/70%                     ..pstcFsFuhTC.lsstpKhusphE..GussstV.................phpsL.sGpphVlhtE.V-tsts....

                 cov    pid 3441         :         .         .         .         .         5         .         . 3520
1 WP_004013458.1 100.0% 100.0%      STTWTVGAAKPVVGSKQADHQGTPHDNAVKFKVDKENEAVSITAENTFEVSGTELTVKKTVTKDNNSEIADSKSFKVSIK
2 WP_101929469.1  37.0%  15.7%      --------GVGVTGTSET---FDPKA---SGATDNGNGYYSGTINMTFQVPVQ-------TIRDNQ--------KLVAFE
  consensus/100%                    ........uhsVsGopps...hsP+s...phtsDptNthhShThp.TFpVssp.......sh+DNp........hhVuhc
  consensus/90%                     ........uhsVsGopps...hsP+s...phtsDptNthhShThp.TFpVssp.......sh+DNp........hhVuhc
  consensus/80%                     ........uhsVsGopps...hsP+s...phtsDptNthhShThp.TFpVssp.......sh+DNp........hhVuhc
  consensus/70%                     ........uhsVsGopps...hsP+s...phtsDptNthhShThp.TFpVssp.......sh+DNp........hhVuhc

                 cov    pid 3521         .         .         :         .         .         .         .         6 3600
1 WP_004013458.1 100.0% 100.0%      CVYPTDKAEHTIATNEPLT-------------NGQS---KTFKTDDTGTAIPVGAKCTVTEDETSAKVAGHTWKLEVKDP
2 WP_101929469.1  37.0%  15.7%      TLYQGDRPETGTKVTEHKDKNDENQTVTEKPRIGTSAEAQGESSDDGKVAVPEEGKDVLI-------KDTVTWNKLK--P
  consensus/100%                    slY.sD+sEpshtssE.hs.............GpS...ps.poDDstsAlP.tuKsslh.......hsshTWph.h..P
  consensus/90%                     slY.sD+sEpshtssE.hs.............GpS...ps.poDDstsAlP.tuKsslh.......hsshTWph.h..P
  consensus/80%                     slY.sD+sEpshtssE.hs.............GpS...ps.poDDstsAlP.tuKsslh.......hsshTWph.h..P
  consensus/70%                     slY.sD+sEpshtssE.hs.............GpS...ps.poDDstsAlP.tuKsslh.......hsshTWph.h..P

                 cov    pid 3601         .         .         .         .         :         .         .         . 3680
1 WP_004013458.1 100.0% 100.0%      NQVSTKGLSAEKDKAKNTVELVNDYKREVGGFKLTKAVAGKGIDFAKAADA---------------NNGKFEFTYDCVL
2 WP_101929469.1  37.0%  15.7%      GTY----------------TLSGQLMKKVGNQVVPVAGTKAAKNFTVNTGQLEGTQELTFTLPAKQIEAGASYVVYEKVF
  consensus/100%                    sph................pLssphh+cVGs.hlshAssttuhsFshssst................psGt..hsY-pVh
  consensus/90%                     sph................pLssphh+cVGs.hlshAssttuhsFshssst................psGt..hsY-pVh
  consensus/80%                     sph................pLssphh+cVGs.hlshAssttuhsFshssst................psGt..hsY-pVh
  consensus/70%                     sph................pLssphh+cVGs.hlshAssttuhsFshssst................psGt..hsY-pVh

                 cov    pid 3681         .         7         .         .         .         .         :         . 3760
1 WP_004013458.1 100.0% 100.0%      DGKTVKNGKGKLDLTKDKQTVEVKDIPTDATCTLKETAPTAPTGFAAP--TAPTWKIGSTTSD-------SGMVTIKE--
2 WP_101929469.1  37.0%  15.7%      KGANNTAG--ILVAS--------HEDPNDKGQ----TVTVNPKQHKLPTIKTVAWVRDDGNSLISTVEADGKDFKVKDKI
  consensus/100%                    cGtsspsG..hLsho........+-.PsDtsp....TssssPptathP..psssWhhssssS........ut.hplK-..
  consensus/90%                     cGtsspsG..hLsho........+-.PsDtsp....TssssPptathP..psssWhhssssS........ut.hplK-..
```

```
  consensus/80%                cGtsspsG..hLsho........+-.PsDtsp....TssssPptathP..psssWhhssssS........ut.hplK-..
  consensus/70%                cGtsspsG..hLsho........+-.PsDtsp....TssssPptathP..psssWhhssssS........ut.hplK-..

                    cov    pid 3761         .         .         .         8         .         .         .         . 3840
1 WP_004013458.1 100.0% 100.0%  --------------------------------------------------------------------------------
2 WP_101929469.1  37.0%  15.7%  IYNDLKPGTYTAFATLVNKDNPKEIIASGRQVFTNGAENGSTLVELAVAGSKVKADGKYVVFERIYQGEQTSEPSGEPYA
  consensus/100%               ................................................................................
  consensus/90%                ................................................................................
  consensus/80%                ................................................................................
  consensus/70%                ................................................................................

                    cov    pid 3841         :         .         .         .         .         9         .         . 3920
1 WP_004013458.1 100.0% 100.0%  -----GQTAEVVATNTYTPYQVKFTL-KKVIKADKTLDGDFKFQVTCGDKTQSVTLNKDNKYTWESSELKTPQILSGVKC
2 WP_101929469.1  37.0%  15.7%  KHQEIGDKSQTITVAPKNPVPAEFKIVKKTVGDNANTDRVFDFDVACDGYTGTVSVVVRKGQTQGETKVSNSALTSGMSC
  consensus/100%               .....GppupslsssshsPh.scFpl.KKsltsstshDtsFcFpVsCsshTtoVolshcpt.T.tpoclpsstlhSGhpC
  consensus/90%                .....GppupslsssshsPh.scFpl.KKsltsstshDtsFcFpVsCsshTtoVolshcpt.T.tpoclpsstlhSGhpC
  consensus/80%                .....GppupslsssshsPh.scFpl.KKsltsstshDtsFcFpVsCsshTtoVolshcpt.T.tpoclpsstlhSGhpC
  consensus/70%                .....GppupslsssshsPh.scFpl.KKsltsstshDtsFcFpVsCsshTtoVolshcpt.T.tpoclpsstlhSGhpC

                    cov    pid 3921         .         .         :         .         .         .         .         0 4000
1 WP_004013458.1 100.0% 100.0%  HLEETSEQAFKSHTWKSLDYQVDGKGVDTSVDKDAKRVTFTLPATGDADVTITATNTYDRNKGALQLQKVAKLNGQDVSK
2 WP_101929469.1  37.0%  15.7%  QV------------------------------------------------------------------------------
  consensus/100%               pl..............................................................................
  consensus/90%                pl..............................................................................
  consensus/80%                pl..............................................................................
  consensus/70%                pl..............................................................................

                    cov    pid 4001         .         .         .         .         :         .         .         . 4080
1 WP_004013458.1 100.0% 100.0%  DAKANPLPNHQYQFEVTCYDPADNGLGAKTQAVTVPLQAGAPPQTVIKDAPVGSVCFIKETPVKTEAVIGTLSWEGDGTN
2 WP_101929469.1  37.0%  15.7%  ----------------------------------------------------------SEQAVQDDQNTVKVQFSGTDTN
  consensus/100%               ..........................................................pEpsVps-tshsplpapGssTN
  consensus/90%                ..........................................................pEpsVps-tshsplpapGssTN
  consensus/80%                ..........................................................pEpsVps-tshsplpapGssTN
  consensus/70%                ..........................................................pEpsVps-tshsplpapGssTN

                    cov    pid 4081         .         1         .         .         .         .         :         . 4160
1 WP_004013458.1 100.0% 100.0%  AVGPDNAANVRKVVIGGEKGDVQQVNVKAVNDLTVQYGIFSLHKELTGDAQNDKDVKSKDFEFTVKCPGLDDQKV--TLK
2 WP_101929469.1  37.0%  15.7%  SDGTVAKFNLT-------ENNAKTVVVTATNTFTPKQAKFTVRKNVEAAGV-THFSVPNSFDFSYKCDGDAYWKAIPTLK
  consensus/100%               usGsssthNlp.......cssppVsVpAsNshTsp.uhFol+Kplpusu..s+.shspsF-FohKCsG.s..Ks..TLK
  consensus/90%                usGsssthNlp.......cssppVsVpAsNshTsp.uhFol+Kplpusu..s+.shspsF-FohKCsG.s..Ks..TLK
  consensus/80%                usGsssthNlp.......cssppVsVpAsNshTsp.uhFol+Kplpusu..s+.shspsF-FohKCsG.s..Ks..TLK
  consensus/70%                usGsssthNlp.......cssppVsVpAsNshTsp.uhFol+Kplpusu..s+.shspsF-FohKCsG.s..Ks..TLK

                    cov    pid 4161         .         .         .         2         .         .         .         . 4240
1 WP_004013458.1 100.0% 100.0%  GGETKTLPEGKMKVGTKCSVHEVPATVAGVTFDPVSWSQNATARTDGGIDVTIPATGVKDLSFTATNKAVYDKATFQIQK
2 WP_101929469.1  37.0%  15.7%  AGETS--AAIKVKPGVECQVKENSGTPENLD-RTLEWLGAQSQ--AGGT-ATFRTDANNTVALVATNTYTEKTGQLEISK
  consensus/100%               uGETp..sthKhKsGscCpV+EssuTstsls.cslpW.tstot..sGGh.sTh.ssuspsluhsATNphs.cpuphpIpK
  consensus/90%                uGETp..sthKhKsGscCpV+EssuTstsls.cslpW.tstot..sGGh.sTh.ssuspsluhsATNphs.cpuphpIpK
  consensus/80%                uGETp..sthKhKsGscCpV+EssuTstsls.cslpW.tstot..sGGh.sTh.ssuspsluhsATNphs.cpuphpIpK
  consensus/70%                uGETp..sthKhKsGscCpV+EssuTstsls.cslpW.tstot..sGGh.sTh.ssuspsluhsATNphs.cpuphpIpK

                    cov    pid 4241         :         .         .         .         .         3         .         . 4320
1 WP_004013458.1 100.0% 100.0%  --VISSTENLAFEDGFKFTYTCKAPNGKTFTQKAPFGAQTGVDTINVKAGQPSGIITVPQGSKCTVSEANPK-DLPKLVN
2 WP_101929469.1  37.0%  15.7%  QVVDGTAGAGTIPGTFEMKYICATGQKAE-------SVTKKLTSISVNKGGSATITGIPVGSQCAVYEDTTGINVPNTTL
  consensus/100%               ..V.uostshsh.ssFchpYhCtsspttp.......uspptlsoIsVptGtsusIhslP.GSpCsV.Essst.slPphs.
  consensus/90%                ..V.uostshsh.ssFchpYhCtsspttp.......uspptlsoIsVptGtsusIhslP.GSpCsV.Essst.slPphs.
  consensus/80%                ..V.uostshsh.ssFchpYhCtsspttp.......uspptlsoIsVptGtsusIhslP.GSpCsV.Essst.slPphs.
  consensus/70%                ..V.uostshsh.ssFchpYhCtsspttp.......uspptlsoIsVptGtsusIhslP.GSpCsV.Essst.slPphs.

                    cov    pid 4321         .         .         :         .         .         .         .         4 4400
1 WP_004013458.1 100.0% 100.0%  DP---NRNPVKYQENGTSIKVGDKTVTG--LTSAEFTADGQMVTVIATNTYVE-KITGFQFKKTLDGN--NAGKYQDQQF
2 WP_101929469.1  37.0%  15.7%  TTTFNGKAPQ-------QVTIGGQVLHNAALSNPIKAGESQKATINVENKYVNQKAGGFEVEKLVAGVTNPGTVLKDVAF
  consensus/100%               ss...s+sP........plplGspslps..Loss.hsu-uQhsTl.spNpYVp.KhsGFphcKhlsGs..sushhpD.tF
  consensus/90%                ss...s+sP........plplGspslps..Loss.hsu-uQhsTl.spNpYVp.KhsGFphcKhlsGs..sushhpD.tF
  consensus/80%                ss...s+sP........plplGspslps..Loss.hsu-uQhsTl.spNpYVp.KhsGFphcKhlsGs..sushhpD.tF
  consensus/70%                ss...s+sP........plplGspslps..Loss.hsu-uQhsTl.spNpYVp.KhsGFphcKhlsGs..sushhpD.tF

                    cov    pid 4401         .         .         .         .         :         .         .         . 4480
1 WP_004013458.1 100.0% 100.0%  SFGYTCKTPKEVRSGDAKL---------KSADDPVKVENLPVGTECTLWENKVNPKNAEKVSTTWTIDGKPVSSGSVTDH
2 WP_101929469.1  37.0%  15.7%  DFGYSCEIPGNAHAVKGSFKLKHGETFTSADPANPQLNNLPVNTVCKVWEETPKSSANADYV------GSELRA---SAT
  consensus/100%               sFGYoCchPtps+uscuph.........pusssssplpNLPVsT.CplWEppspspsstchs......Gp.lpu...osp
  consensus/90%                sFGYoCchPtps+uscuph.........pusssssplpNLPVsT.CplWEppspspsstchs......Gp.lpu...osp
  consensus/80%                sFGYoCchPtps+uscuph.........pusssssplpNLPVsT.CplWEppspspsstchs......Gp.lpu...osp
  consensus/70%                sFGYoCchPtps+uscuph.........pusssssplpNLPVsT.CplWEppspspsstchs......Gp.lpu...osp

                    cov    pid 4481         .         5         .         .         .         .         :         . 4560
1 WP_004013458.1 100.0% 100.0%  NGVKHTNA---NGVTFKVETEGTPVNVVATNKFDIPSTSFKIHKTVTNKTDGYHVPADKGFKFDVTCTYPTDDAEHVIAK
2 WP_101929469.1  37.0%  15.7%  NGAEVTGTDGKLELTIKLNKAAAKAKILATNQYSQQLGGFTLKKEVQGSADQAL--DIQSYKFKL--TYMDGTNTHVKE-
  consensus/100%               NGschTss....tlThKlpptus.spllATNpas...suFpl+KpVpspsDthh..s.puaKFcl..TY.sssspHVht.
  consensus/90%                NGschTss....tlThKlpptus.spllATNpas...suFpl+KpVpspsDthh..s.puaKFcl..TY.sssspHVht.
  consensus/80%                NGschTss....tlThKlpptus.spllATNpas...suFpl+KpVpspsDthh..s.puaKFcl..TY.sssspHVht.
  consensus/70%                NGschTss....tlThKlpptus.spllATNpas...suFpl+KpVpspsDthh..s.puaKFcl..TY.sssspHVht.
```

```
                          cov    pid 4561          .         .         .         6         .         .         .         . 4640
1 WP_004013458.1 100.0% 100.0%      DELVKGGQDSKNFEADVDGVPIPVGASCTVTEKNAAVANYSLAVTMKDGGKALTVETKQADQTKSEPGYTAKVITTVEAK
2 WP_101929469.1  37.0%  15.7%      QTIPAGG-GSYSFW------GVPVGSQVQVEELGAVD----------SQGNLIPVNELSTAKVKHSISWNG---------
  consensus/100%                    pplstGG.sShsF.......slPVGupspVpEhsAss..........stGphlsVpphpsspsKpp.uasu.........
  consensus/90%                     pplstGG.sShsF.......slPVGupspVpEhsAss..........stGphlsVpphpsspsKpp.uasu.........
  consensus/80%                     pplstGG.sShsF.......slPVGupspVpEhsAss..........stGphlsVpphpsspsKpp.uasu.........
  consensus/70%                     pplstGG.sShsF.......slPVGupspVpEhsAss..........stGphlsVpphpsspsKpp.uasu.........

                          cov    pid 4641          :         .         .         .         .         7         .         . 4720
1 WP_004013458.1 100.0% 100.0%      TVNVENTYTRDFGIFKIEKKTTAQSYIEKPSSFDFKYTCKDPKDSKAEVKQGILNVGEAAVSSQQIPVGYKCEVSEVDPK
2 WP_101929469.1  37.0%  15.7%      --TQDDSSTASANIFTIN------------------------------------------------------------KA
  consensus/100%                    ..s.-so.TtshsIFpIp.............................................................t
  consensus/90%                     ..s.-so.TtshsIFpIp.............................................................t
  consensus/80%                     ..s.-so.TtshsIFpIp.............................................................t
  consensus/70%                     ..s.-so.TtshsIFpIp.............................................................t

                          cov    pid 4721          .         .         :         .         .         .         .         8 4800
1 WP_004013458.1 100.0% 100.0%      VKGAKWAHTLSQKTFVIQKSEVQTASLTATNDFTDSTGKFKVTKKV----KNDDKLALPLNATYNFEWWCGPKGAEAPQK
2 WP_101929469.1  37.0%  15.7%      MTEAQWKESGKQANVVVKATNT--------YKAIEQKGTFSVQKTAVKVAGESKDKVKNLPTSYNFTYDCSDGIHGNIPN
  consensus/100%                    hptApWtcohpQtshVlptops.........chh-ppGpFpVpKps....tpscchshsLssoYNFpa.Custhtts..p
  consensus/90%                     hptApWtcohpQtshVlptops.........chh-ppGpFpVpKps....tpscchshsLssoYNFpa.Custhtts..p
  consensus/80%                     hptApWtcohpQtshVlptops.........chh-ppGpFpVpKps....tpscchshsLssoYNFpa.Custhtts..p
  consensus/70%                     hptApWtcohpQtshVlptops.........chh-ppGpFpVpKps....tpscchshsLssoYNFpa.Custhtts..p

                          cov    pid 4801          .         .         .         .         :         .         .         . 4880
1 WP_004013458.1 100.0% 100.0%      WNTFSLKQGETFESGEIASGSECGIREV--KPTGDFAAVGANVEVTWSLAETKTTQGAT-----------SVKAPVLVDS
2 WP_101929469.1  37.0%  15.7%      ---VKPGGEPVVAPAQFRAGTVCTLTEQQVLPKGT-----NAQTVSWTVDGKGKGVGPAAGTSATVTIGSDAKHPVAVQA
  consensus/100%                    ...hp.ttt.shtsuphtuGo.CslpE...hPpGs.....ss.pVoWolstptps.Gss...........ssKtPVhVpu
  consensus/90%                     ...hp.ttt.shtsuphtuGo.CslpE...hPpGs.....ss.pVoWolstptps.Gss...........ssKtPVhVpu
  consensus/80%                     ...hp.ttt.shtsuphtuGo.CslpE...hPpGs.....ss.pVoWolstptps.Gss...........ssKtPVhVpu
  consensus/70%                     ...hp.ttt.shtsuphtuGo.CslpE...hPpGs.....ss.pVoWolstptps.Gss...........ssKtPVhVpu

                          cov    pid 4881          .         9         .         .         .         .         :         . 4960
1 WP_004013458.1 100.0% 100.0%      EQAFVFGKQGDSAGK-FTIGKNGSEQILTATNEVKQTYVDLSLEKVAQSSNNNGDPGLSVDKFNYWNSYYQAWGGFVTRN
2 WP_101929469.1  37.0%  15.7%      ANEYQTPDEPKIATELKGEGRNGNIDLVE----------------------GADHTYTLTDKISYWNLEVGKTYKFSGEL
  consensus/100%                    tpta.hscpsc.Asc.hs.G+NGs.pllp......................ssscshh.sDKhsYWN..htthhtFssc.
  consensus/90%                     tpta.hscpsc.Asc.hs.G+NGs.pllp......................ssscshh.sDKhsYWN..htthhtFssc.
  consensus/80%                     tpta.hscpsc.Asc.hs.G+NGs.pllp......................ssscshh.sDKhsYWN..htthhtFssc.
  consensus/70%                     tpta.hscpsc.Asc.hs.G+NGs.pllp......................ssscshh.sDKhsYWN..htthhtFssc.

                          cov    pid 4961          .         .         .         0         .         .         .         . 5040
1 WP_004013458.1 100.0% 100.0%      FVEMQVTCNDKPVADKAH---PEGIWKVPLNGKIDLPVKVAIGSKCTVAELDKGFNDYKMLFYGRWRHSVDMSAKSLDGI
2 WP_101929469.1  37.0%  15.7%      ----VVPNADKTDATRTGIKAEKTVTITTPSGVVELPFKLTQADVA----------KYSTL--------V-----AFEKL
  consensus/100%                    .....VsssDKssAs+st....cslhhss.sGhl-LPhKls.ushs..........cYphL........V.....uh-tl
  consensus/90%                     .....VsssDKssAs+st....cslhhss.sGhl-LPhKls.ushs..........cYphL........V.....uh-tl
  consensus/80%                     .....VsssDKssAs+st....cslhhss.sGhl-LPhKls.ushs..........cYphL........V.....uh-tl
  consensus/70%                     .....VsssDKssAs+st....cslhhss.sGhl-LPhKls.ushs..........cYphL........V.....uh-tl

                          cov    pid 5041          :         .         .         .         .         1         .         . 5120
1 WP_004013458.1 100.0% 100.0%      DVSKRYDFDAVEDDKAQAAIDFDVSGKTKVEVKMTNNWDCFSKVTSELGTNLTDAKKVNGVRQIDLSEKKSDGTNKYTDW
2 WP_101929469.1  37.0%  15.7%      DLQVEGGFEPVTKHE---------DPKDENQTKQVNPWIATV---------LTD---EGGNKQIDTTGK------ADSEL
  consensus/100%                    DlphchsF-sVpccc.........ssKscspsK.sNsW.shs.........LTD....sGs+QIDhotK......t.o-h
  consensus/90%                     DlphchsF-sVpccc.........ssKscspsK.sNsW.shs.........LTD....sGs+QIDhotK......t.o-h
  consensus/80%                     DlphchsF-sVpccc.........ssKscspsK.sNsW.shs.........LTD....sGs+QIDhotK......t.o-h
  consensus/70%                     DlphchsF-sVpccc.........ssKscspsK.sNsW.shs.........LTD....sGs+QIDhotK......t.o-h

                          cov    pid 5121          .         .         :         .         .         .         .         2 5200
1 WP_004013458.1 100.0% 100.0%      VTLTDVVEFKNLPK-RNYTVMGRLFNASKSGK---LTAADFNNPNQYLNWKNPQGHTWNYVYQEVNADSAKVTLTYEIPV
2 WP_101929469.1  37.0%  15.7%      VTLVDTVKYRNLEAGKTYHLTGKLVDGSGKPIGVSSTSKAFTVPGEAG-----------------TLQSGEAKMTFQVTV
  consensus/100%                    VTLsDsVca+NL.t.+sYplhG+LhsuStpsh....TutsFssPsphh.................shpSucsphTaplsV
  consensus/90%                     VTLsDsVca+NL.t.+sYplhG+LhsuStpsh....TutsFssPsphh.................shpSucsphTaplsV
  consensus/80%                     VTLsDsVca+NL.t.+sYplhG+LhsuStpsh....TutsFssPsphh.................shpSucsphTaplsV
  consensus/70%                     VTLsDsVca+NL.t.+sYplhG+LhsuStpsh....TutsFssPsphh.................shpSucsphTaplsV

                          cov    pid 5201          .         .         .         .         :         .         .         . 5280
1 WP_004013458.1 100.0% 100.0%      SLFEANPDGIAAGVAVYRSDYWNPQCAGNCGLMAVEPTL-PQNQQVKPIWKGELKTQVLNPVTKSNMLQVGKGAPEIQSI
2 WP_101929469.1  37.0%  15.7%      GQIRQ-------NAKLVAFEYLHPGQPNTPGDETVITKHEDPNDENQTVNEGPRAATTAQSSTGTKVF----DAKTPAKV
  consensus/100%                    u.hct.......sstlht.-YhpPtpssssG..sV.sph.s.Nppspsl.cG.htspshpssTtophh....sA.p.tpl
  consensus/90%                     u.hct.......sstlht.-YhpPtpssssG..sV.sph.s.Nppspsl.cG.htspshpssTtophh....sA.p.tpl
  consensus/80%                     u.hct.......sstlht.-YhpPtpssssG..sV.sph.s.Nppspsl.cG.htspshpssTtophh....sA.p.tpl
  consensus/70%                     u.hct.......sstlht.-YhpPtpssssG..sV.sph.s.Nppspsl.cG.htspshpssTtophh....sA.p.tpl

                          cov    pid 5281          .         3         .         .         .         .         :         . 5360
1 WP_004013458.1 100.0% 100.0%      ADTVTYTNVAPGSYDLYGRIVALGNEGLVIGEGTANVTTAEAGSNALSSGEWNNDQIKVNTASLEDAVKNDAKGFVTYEF
2 WP_101929469.1  37.0%  15.7%      LDRVTWSKLPAGNYVLVGTLMDKGTKQAVPNVNAEIV------KFTVSNGELKGLQEMT--FTVPAEQAKSNAQFVAFEK
  consensus/100%                    hDpVTaoplssGsYsLhGplhshGscthV.s.sst.V......p.slSsGEhps.Q.hs..hol.st.tpssttFVsaEh
  consensus/90%                     hDpVTaoplssGsYsLhGplhshGscthV.s.sst.V......p.slSsGEhps.Q.hs..hol.st.tpssttFVsaEh
  consensus/80%                     hDpVTaoplssGsYsLhGplhshGscthV.s.sst.V......p.slSsGEhps.Q.hs..hol.st.tpssttFVsaEh
  consensus/70%                     hDpVTaoplssGsYsLhGplhshGscthV.s.sst.V......p.slSsGEhps.Q.hs..hol.st.tpssttFVsaEh

                          cov    pid 5361          .         .         .         4         .         .         .         . 5440
```

```
1 WP_004013458.1 100.0% 100.0%      LLPKGSATSGAAVETLKGQAVASHVDDNDVMQ---------QLFTPTLRTTATVGSENGTKTVDANATGTVEVFDKVEYR
2 WP_101929469.1  37.0%  15.7%      IYRQGDVDENGNVKDGKK-PVVEHSDLNDKAQTVTTGEEPQSPVTPTLTLQKVVKS---------------------EGL
  consensus/100%                    lh.pGssspsusVcshKt.sVspHsD.NDhhQ.........p.hTPTLphptsVtS.....................Ehh
  consensus/90%                     lh.pGssspsusVcshKt.sVspHsD.NDhhQ.........p.hTPTLphptsVtS.....................Ehh
  consensus/80%                     lh.pGssspsusVcshKt.sVspHsD.NDhhQ.........p.hTPTLphptsVtS.....................Ehh
  consensus/70%                     lh.pGssspsusVcshKt.sVspHsD.NDhhQ.........p.hTPTLphptsVtS.....................Ehh

                    cov     pid 5441         :         .         .         .         .         5         .         . 5520
1 WP_004013458.1 100.0% 100.0%      NLPGGKKYTVTGK---------------------LHFK--------SADGTDGGELPGVDSASGTIDLTSQKATEGHSGT
2 WP_101929469.1  37.0%  15.7%      EVPSTKQFAFTLKCSSPIDGKSKQQTYQVTAGSKISLPYVKGANCTLTEDREGAKVAGIAPSQVTFSADSEKIGL-ITSD
  consensus/100%                    plPusKpashThK.....................lph..........s-sp-GuclsGlssupsThshsSpKhs..hous
  consensus/90%                     plPusKpashThK.....................lph..........s-sp-GuclsGlssupsThshsSpKhs..hous
  consensus/80%                     plPusKpashThK.....................lph..........s-sp-GuclsGlssupsThshsSpKhs..hous
  consensus/70%                     plPusKpashThK.....................lph..........s-sp-GuclsGlssupsThshsSpKhs..hous

                    cov     pid 5521         .         .         :         .         .         .         .         6 5600
1 WP_004013458.1 100.0% 100.0%      VTLKFTVPVEKLQQKIVVAFEEVKDGNILVAAHADITDEAQTVYSPKIGTTASAAPGFSATADTPATVDMSNAKPLKGKD
2 WP_101929469.1  37.0%  15.7%      NSASFKMPAEE-----------TTEVNVVLTATNTY----PAPEKPTVATSAK---------------DANGTKGMYGQS
  consensus/100%                    sohpFphPsEc...........sp-sNlllsApssh.....ss.pPpluToAp...............DhsssKshhGps
  consensus/90%                     sohpFphPsEc...........sp-sNlllsApssh.....ss.pPpluToAp...............DhsssKshhGps
  consensus/80%                     sohpFphPsEc...........sp-sNlllsApssh.....ss.pPpluToAp...............DhsssKshhGps
  consensus/70%                     sohpFphPsEc...........sp-sNlllsApssh.....ss.pPpluToAp...............DhsssKshhGps

                    cov     pid 5601         .         .         .         .         :         .         .         . 5680
1 WP_004013458.1 100.0% 100.0%      SR---KAELGNVAVIDTVEYHGLEAGKYVLYGELKKLSDGKEVSTAKTSSHSFTVDAVTGV-HGTQSVTFSDLKVQSGET
2 WP_101929469.1  37.0%  15.7%      KQANQVYAGTSAKIVDEVSWANLLPGDYVLIGKLMDKETGKAVTSYTSTPAPFTVKAGEKNGKLDNTFTVSGDSLKAGSH
  consensus/100%                    pp...hhthssstllDpVpatsL.sGcYVLhGcLhchpsGKtVoohpoostsFTVcAspts.+hspohThSs.plpuGpp
  consensus/90%                     pp...hhthssstllDpVpatsL.sGcYVLhGcLhchpsGKtVoohpoostsFTVcAspts.+hspohThSs.plpuGpp
  consensus/80%                     pp...hhthssstllDpVpatsL.sGcYVLhGcLhchpsGKtVoohpoostsFTVcAspts.+hspohThSs.plpuGpp
  consensus/70%                     pp...hhthssstllDpVpatsL.sGcYVLhGcLhchpsGKtVoohpoostsFTVcAspts.+hspohThSs.plpuGpp

                    cov     pid 5681         .         7         .         .         .         .         :         . 5760
1 WP_004013458.1 100.0% 100.0%      YVVYEYLFKAGDFQVDKPDTKKA-IASHADPEDKGQTVTVPGVTTDATDGDGDKYVDSSQNFTIKDTVTATGLIP--GKT
2 WP_101929469.1  37.0%  15.7%      YVVYEYIYRAGDVNGATPNNGAKPVVEHAVITDADQTVEAI--TPPTPP---------AAKVKIMKKVVASGMEVSNNRA
  consensus/100%                    YVVYEYla+AGDhpsspPssttt.lspHAs.pDtsQTVps...Tsssss.........utphpIhcpVsAoGh.s..s+s
  consensus/90%                     YVVYEYla+AGDhpsspPssttt.lspHAs.pDtsQTVps...Tsssss.........utphpIhcpVsAoGh.s..s+s
  consensus/80%                     YVVYEYla+AGDhpsspPssttt.lspHAs.pDtsQTVps...Tsssss.........utphpIhcpVsAoGh.s..s+s
  consensus/70%                     YVVYEYla+AGDhpsspPssttt.lspHAs.pDtsQTVps...Tsssss.........utphpIhcpVsAoGh.s..s+s

                    cov     pid 5761         .         .         .         8         .         .         .         . 5840
1 WP_004013458.1 100.0% 100.0%      YDVSGELMVDDGTPQGATT------------GIKQT------------------GTIT----------AKADGTGETVLE
2 WP_101929469.1  37.0%  15.7%      YIFTLSCRDKDGNAAGDKTLLVRPDKATEVTGLRSGYVCSINEDLDAAIEQQVTPSISLTSGTNGFSVQQDSTAGAAHFT
  consensus/100%                    Y.hohphhscDGsstGspT............Gl+ps..................soIo..........tpssssGtshhp
  consensus/90%                     Y.hohphhscDGsstGspT............Gl+ps..................soIo..........tpssssGtshhp
  consensus/80%                     Y.hohphhscDGsstGspT............Gl+ps..................soIo..........tpssssGtshhp
  consensus/70%                     Y.hohphhscDGsstGspT............Gl+ps..................soIo..........tpssssGtshhp

                    cov     pid 5841         :         .         .         .         .         9         .         . 5920
1 WP_004013458.1 100.0% 100.0%      FPVTAQQAQD--------LGLVGKPIVVFEDLSLDGKKVAVHHDIKDEKQTVYNGGLKTKAVDAADENQAMVPGQKSAAV
2 WP_101929469.1  37.0%  15.7%      VPAATEQVPEVLVNVTNTYNKMGKPE------------------------------LQTNAVT--DNGSSSVQAGTATTV
  consensus/100%                    hPssspQs.-........hshhGKP...............................LpTpAVs..Dpspu.V.utpussV
  consensus/90%                     hPssspQs.-........hshhGKP...............................LpTpAVs..Dpspu.V.utpussV
  consensus/80%                     hPssspQs.-........hshhGKP...............................LpTpAVs..Dpspu.V.utpussV
  consensus/70%                     hPssspQs.-........hshhGKP...............................LpTpAVs..Dpspu.V.utpussV

                    cov     pid 5921         .         .         :         .         .         .         .         0 6000
1 WP_004013458.1 100.0% 100.0%      VDTVTFNGRFEKSHSYTLVGELHYVN---GTVVPGTKTETKTFQSDQD-GAIAAQKMTFTVPAEYIKAGQNMVVFEKLFD
2 WP_101929469.1  37.0%  15.7%      TDKVTWKNL--PEGKYLLTGKLMHITDDNAAPVAGVTNEPVVLEITKDNSLAGSTTMKFNVPAGAISQAGKYVVYEYLYN
  consensus/100%                    sDpVTapsh...ptpYhLsGcLhals...ussVsGspsEshshp.spD.uhhuuppMpFsVPAthIptutphVVaEhLas
  consensus/90%                     sDpVTapsh...ptpYhLsGcLhals...ussVsGspsEshshp.spD.uhhuuppMpFsVPAthIptutphVVaEhLas
  consensus/80%                     sDpVTapsh...ptpYhLsGcLhals...ussVsGspsEshshp.spD.uhhuuppMpFsVPAthIptutphVVaEhLas
  consensus/70%                     sDpVTapsh...ptpYhLsGcLhals...ussVsGspsEshshp.spD.uhhuuppMpFsVPAthIptutphVVaEhLas

                    cov     pid 6001         .         .         .         .         :         .         .         . 6080
1 WP_004013458.1 100.0% 100.0%      AKKKDGT-------PVASHEDPNDPDQTITVQEVEITTTA--YDGAAGDKSDPKDKNLDASKETVTIYDQVDYKGLNVGE
2 WP_101929469.1  37.0%  15.7%      YEDTDGHKPKPNTSTVVSHNDPSDDAQTVNVTEAPSVSTTATTDGAN-------EKEIQKG-KAAVVTDTVNWKNLPAGN
  consensus/100%                    hccpDGp.......sVsSHpDPsDssQTlsVpEs..soTs..hDGAs.......-Kplptu.cssslhDpVsaKsLssGp
  consensus/90%                     hccpDGp.......sVsSHpDPsDssQTlsVpEs..soTs..hDGAs.......-Kplptu.cssslhDpVsaKsLssGp
  consensus/80%                     hccpDGp.......sVsSHpDPsDssQTlsVpEs..soTs..hDGAs.......-Kplptu.cssslhDpVsaKsLssGp
  consensus/70%                     hccpDGp.......sVsSHpDPsDssQTlsVpEs..soTs..hDGAs.......-Kplptu.cssslhDpVsaKsLssGp

                    cov     pid 6081         .         1         .         .         .         .         :         . 6160
1 WP_004013458.1 100.0% 100.0%      EYTITGTLHYQADATLADGTQVKRGDEVPAQYVNVTPVKITANKASSDESGAVKAIVKFEVQKTALATAPVVVFETLYQG
2 WP_101929469.1  37.0%  15.7%      YQALSELVDS-------------KGNPVAGATTKAVPF----TVNNGEEAGSVKTKIQLPATATSTAGAKFVVFERIYRA
  consensus/100%                    ..slothlc..............+Gs.VsuthspssPh....shssu-EuGuVKshlph.sptTuhAsA.hVVFEplYpu
  consensus/90%                     ..slothlc..............+Gs.VsuthspssPh....shssu-EuGuVKshlph.sptTuhAsA.hVVFEplYpu
  consensus/80%                     ..slothlc..............+Gs.VsuthspssPh....shssu-EuGuVKshlph.sptTuhAsA.hVVFEplYpu
  consensus/70%                     ..slothlc..............+Gs.VsuthspssPh....shssu-EuGuVKshlph.sptTuhAsA.hVVFEplYpu

                    cov     pid 6161         .         .         .         2         .         .         .         . 6240
1 WP_004013458.1 100.0% 100.0%      TVEVATH-QDIDDGSQVVYHPS-------LRTLATVNGAKVIQMKKDSKENLTVTDQITWANLAPGTYTLEGSLMEVKDG
2 WP_101929469.1  37.0%  15.7%      SDVDSQTGRPAQNAQPVVSELDLNAINQTVRVVESPKGPPAITFTKVTE--------------------------KALDG
```

```
  consensus/100%                    os.supp.pshpsup.VV.c.s.......lRsltospGs.sIphpKsoc..........................cshDG
  consensus/90%                     os.supp.pshpsup.VV.c.s.......lRsltospGs.sIphpKsoc..........................cshDG
  consensus/80%                     os.supp.pshpsup.VV.c.s.......lRsltospGs.sIphpKsoc..........................cshDG
  consensus/70%                     os.supp.pshpsup.VV.c.s.......lRsltospGs.sIphpKsoc..........................cshDG

                     cov    pid 6241          :         .         .         .         .         3         .         . 6320
1 WP_004013458.1 100.0% 100.0%      QLVSNTPVAKGQTQKVEVAAGKAGATTSTGEAQMTFKLPVDKVKSGSQFVVYQILKDKSGQVVATHADPKSDDQTVTVGS
2 WP_101929469.1  37.0%  15.7%      QVPD-------------------------KDRKFQFTISCDKNFGGAYS--FEMP---AGVSAALPA-----DKQLQAGT
  consensus/100%                    Qlss.........................t-tphpFplssDKshuGu....aph....uG.ssAh.A.....DpplpsGo
  consensus/90%                     Qlss.........................t-tphpFplssDKshuGu....aph....uG.ssAh.A.....DpplpsGo
  consensus/80%                     Qlss.........................t-tphpFplssDKshuGu....aph....uG.ssAh.A.....DpplpsGo
  consensus/70%                     Qlss.........................t-tphpFplssDKshuGu....aph....uG.ssAh.A.....DpplpsGo

                     cov    pid 6321          .         .         :         .         .         .         .         4 6400
1 WP_004013458.1 100.0% 100.0%      LDTTATDAADGNKHADNAAAVTINDKVDYSGLNLAATYPDGTLKAYLVRGELMDKATGKPVAGVAPVERVIGAANSVYRV
2 WP_101929469.1  37.0%  15.7%      VCT---------------------------------------L-----TEMLTDEA---NADGILPDAISFSSSNKAMAV
  consensus/100%                    lsT.......................................L.....pt.LhDcA...sssGlhPsthshuuuNpshtV
  consensus/90%                     lsT.......................................L.....pt.LhDcA...sssGlhPsthshuuuNpshtV
  consensus/80%                     lsT.......................................L.....pt.LhDcA...sssGlhPsthshuuuNpshtV
  consensus/70%                     lsT.......................................L.....pt.LhDcA...sssGlhPsthshuuuNpshtV

                     cov    pid 6401          .         .         .         .         :         .         .         . 6480
1 WP_004013458.1 100.0% 100.0%      GDQNRPVEEEITSGAGSVVLSFQVPAKLTQ--GKVLVAFETVYEEGREFLIHHDINDDAQTVYTPSVKTQARVDSERN--
2 WP_101929469.1  37.0%  15.7%      SKI------------NNQTAQFTVPDVDTKVVPEIQVTIKNIYSKD-----------------FPELGTIARDNADQDKV
  consensus/100%                    uc.............ss.shpFpVPsh.Tp..scl.VshcslYpcs.................hPpltT.ARssu-ps..
  consensus/90%                     uc.............ss.shpFpVPsh.Tp..scl.VshcslYpcs.................hPpltT.ARssu-ps..
  consensus/80%                     uc.............ss.shpFpVPsh.Tp..scl.VshcslYpcs.................hPpltT.ARssu-ps..
  consensus/70%                     uc.............ss.shpFpVPsh.Tp..scl.VshcslYpcs.................hPpltT.ARssu-ps..

                     cov    pid 6481          .         5         .         .         .         .         :         . 6560
1 WP_004013458.1 100.0% 100.0%      LLL-ADKDSTIKDTVTLSGLKTGETYVLSGVLMDKATGQPVLGKDMQAITAVSEPLKAESGAFVKTDAVSFTVPAGTVKA
2 WP_101929469.1  37.0%  15.7%      LDLKKGENAQVQDVATWKNLA-PGKYTMMGTLMDKLTGKPVVGANTPAVDFQ-----VKKGEKTGTIYALFTVPGDKVST
  consensus/100%                    L.L.tscsuplpDssThpsLt.stpYsh.GsLMDKhTGpPVlGtsh.Alsh......scpGthstT.hs.FTVPuspVps
  consensus/90%                     L.L.tscsuplpDssThpsLt.stpYsh.GsLMDKhTGpPVlGtsh.Alsh......scpGthstT.hs.FTVPuspVps
  consensus/80%                     L.L.tscsuplpDssThpsLt.stpYsh.GsLMDKhTGpPVlGtsh.Alsh......scpGthstT.hs.FTVPuspVps
  consensus/70%                     L.L.tscsuplpDssThpsLt.stpYsh.GsLMDKhTGpPVlGtsh.Alsh......scpGthstT.hs.FTVPuspVps

                     cov    pid 6561          .         .         .         6         .         .         .         . 6640
1 WP_004013458.1 100.0% 100.0%      DTELVVFEKLWVANEVTVDTKTKTVTPKDTKTGKSQPAASHEDITDENQTVKSGTSPSLKTVLS------ADGKREWV--
2 WP_101929469.1  37.0%  15.7%      KASWVVFEKVYKASDVKDGKVVS----------KATPVVDHSNLEDADQTVQVTPPPASQEQKTREIATVAKNGTTFNDG
  consensus/100%                    csphVVFEKlahAs-Vpssphsp..........KupPsssHpslpDtsQTVpssssPu.pp.ho......Acstppas..
  consensus/90%                     csphVVFEKlahAs-Vpssphsp..........KupPsssHpslpDtsQTVpssssPu.pp.ho......Acstppas..
  consensus/80%                     csphVVFEKlahAs-Vpssphsp..........KupPsssHpslpDtsQTVpssssPu.pp.ho......Acstppas..
  consensus/70%                     csphVVFEKlahAs-Vpssphsp..........KupPsssHpslpDtsQTVpssssPu.pp.ho......Acstppas..

                     cov    pid 6641          :         .         .         .         .         7         .         . 6720
1 WP_004013458.1 100.0% 100.0%      --ENNTNIPTV-PHASDSLIDTVLYTGLTEGVSYRLDAKLMEINPVT--------GKV-SETPVATGYTEFTAKTSDGTA
2 WP_101929469.1  37.0%  15.7%      TKDPNLGKPVLTPGQDAVIVDTVKWKNLEPG-EYTITGSLMDKSTNAPLTYLDKDGNQKDGATAERGSFAVKEGQTEGET
  consensus/100%                    ..-sNhshPsl.PttsssllDTVhapsLp.G.pYplsupLM-hssss........Gp..stssstpG.hthpttpo-Gps
  consensus/90%                     ..-sNhshPsl.PttsssllDTVhapsLp.G.pYplsupLM-hssss........Gp..stssstpG.hthpttpo-Gps
  consensus/80%                     ..-sNhshPsl.PttsssllDTVhapsLp.G.pYplsupLM-hssss........Gp..stssstpG.hthpttpo-Gps
  consensus/70%                     ..-sNhshPsl.PttsssllDTVhapsLp.G.pYplsupLM-hssss........Gp..stssstpG.hthpttpo-Gps

                     cov    pid 6721          .         .         :         .         .         .         .         8 6800
1 WP_004013458.1 100.0% 100.0%      QVTFNGITGKLKAGYKYVAYEKMTRPGQPDKPVP-----------------------------PPHEDPKDPNQTVVSEH
2 WP_101929469.1  37.0%  15.7%      KVYFTVKGEAIQANRQYVVFEDLYKTADIDEHGQPTPGAEKVAQHHDINDAAQTLSGDKPKPGTPPETPKTPNT------
  consensus/100%                    pVhFsshsttlpAshpYVsaEchh+sup.Dc.s..............................sP.EsPKsPNp......
  consensus/90%                     pVhFsshsttlpAshpYVsaEchh+sup.Dc.s..............................sP.EsPKsPNp......
  consensus/80%                     pVhFsshsttlpAshpYVsaEchh+sup.Dc.s..............................sP.EsPKsPNp......
  consensus/70%                     pVhFsshsttlpAshpYVsaEchh+sup.Dc.s..............................sP.EsPKsPNp......

                     cov    pid 6801          .         .         .         .         :         .         .         . 6880
1 WP_004013458.1 100.0% 100.0%      NPGITTTLTDAQAAKGTDGKVISLTRD-AQLKDVVRVT-QTGLIEGAKYHVFSKLVNQANPDQVVSAGMQEFTATGDQLR
2 WP_101929469.1  37.0%  15.7%      -PGTTPPGPTPRTPSGFRGVVSTVLAKTGSTTGIMAMTGVLAMVAGIGLVMIRRYQREELED------------------
  consensus/100%                    .PGhTsshssspsspGhcGhV.olhtc.uphpslhthT..huhltGhthhhhp+h.ppt..D..................
  consensus/90%                     .PGhTsshssspsspGhcGhV.olhtc.uphpslhthT..huhltGhthhhhp+h.ppt..D..................
  consensus/80%                     .PGhTsshssspsspGhcGhV.olhtc.uphpslhthT..huhltGhthhhhp+h.ppt..D..................
  consensus/70%                     .PGhTsshssspsspGhcGhV.olhtc.uphpslhthT..huhltGhthhhhp+h.ppt..D..................

                     cov    pid 6881          .         9         .         .         .         .         :         . 6960
1 WP_004013458.1 100.0% 100.0%      SVTVKFTVPKETLQELAGSDPSAEFKLVAYEYLALDSDTDIVNKEATSEIEAVGFKTGKTWAATHADPNDAGQTVTVVKA
2 WP_101929469.1  37.0%  15.7%      --------------------------------------------------------------------------------
  consensus/100%                    ................................................................................
  consensus/90%                     ................................................................................
  consensus/80%                     ................................................................................
  consensus/70%                     ................................................................................

                     cov    pid 6961          .         .         .         0         .         .         .         . 7040
1 WP_004013458.1 100.0% 100.0%      PKIGTTLKYGQSKTVWVADKVELTDTVEYFNLQPKTKYTLSGNLMGGTSAESLSDTGVKATTEFTTPAAANGAQTVSGTA
2 WP_101929469.1  37.0%  15.7%      --------------------------------------------------------------------------------
  consensus/100%                    ................................................................................
  consensus/90%                     ................................................................................
```

```
  consensus/80%                      ................................................................................
  consensus/70%                      ................................................................................

                    cov    pid 7041           :         .         .         .         .         1         .         . 7120
1 WP_004013458.1 100.0% 100.0%       VVKFTVPREVLERNEKLVAYEYLTIDGNPVASHEDPKDENQTVTSKKPGVGTYATVDKLKAFDVTDGKKDAFTIKDTVRL
2 WP_101929469.1  37.0%  15.7%       --------------------------------------------------------------------------------
  consensus/100%                     ................................................................................
  consensus/90%                      ................................................................................
  consensus/80%                      ................................................................................
  consensus/70%                      ................................................................................

                    cov    pid 7121           .         .         :         .         .         .         .         2 7200
1 WP_004013458.1 100.0% 100.0%       YNVEEGKTYAIAGQLYEQSVAGDEGSALAKAATTVKVTASMAKPATEVEKTKYGEDVKVYETEMDLTVKREDLTKNQVVK
2 WP_101929469.1  37.0%  15.7%       --------------------------------------------------------------------------------
  consensus/100%                     ................................................................................
  consensus/90%                      ................................................................................
  consensus/80%                      ................................................................................
  consensus/70%                      ................................................................................

                    cov    pid 7201           .         .         .         .         :         .         .         . 7280
1 WP_004013458.1 100.0% 100.0%       DDIALVVYEQLWAEGTYEKVNDTEVTPKGKSEPVAKHNDPQSSSQSITAEPQFGSLKLTKTVTGWEDAFAKVARPEASYK
2 WP_101929469.1  37.0%  15.7%       --------------------------------------------------------------------------------
  consensus/100%                     ................................................................................
  consensus/90%                      ................................................................................
  consensus/80%                      ................................................................................
  consensus/70%                      ................................................................................

                    cov    pid 7281           .         3         .         .         .         .         :         . 7360
1 WP_004013458.1 100.0% 100.0%       FTVKCVQKGSVDEFTLKEGEEKTVEGIPLGDTCTISEDVQGAVNQAGLKDTVKFTAVNGVTVDSQVNGEAVVKIGGTANG
2 WP_101929469.1  37.0%  15.7%       --------------------------------------------------------------------------------
  consensus/100%                     ................................................................................
  consensus/90%                      ................................................................................
  consensus/80%                      ................................................................................
  consensus/70%                      ................................................................................

                    cov    pid 7361           .         .         .         4         .         .         .         . 7440
1 WP_004013458.1 100.0% 100.0%       SDTVANVEVTAENSFSYDPVITTNTISQFGKVLENGGVLTDTVTYKQMPAGNYVLHTYFVEMVKDEATGKTVAKKIDYVP
2 WP_101929469.1  37.0%  15.7%       --------------------------------------------------------------------------------
  consensus/100%                     ................................................................................
  consensus/90%                      ................................................................................
  consensus/80%                      ................................................................................
  consensus/70%                      ................................................................................

                    cov    pid 7441           :         .         .         .         .         5         .         . 7520
1 WP_004013458.1 100.0% 100.0%       SYVTEQTVKADATTPENGYNGTWTVSVDIPDTLHEVGKKVVVWQDVYVAPQTADMTKFKDGLNKLSAGETSKVAKLVVSH
2 WP_101929469.1  37.0%  15.7%       --------------------------------------------------------------------------------
  consensus/100%                     ................................................................................
  consensus/90%                      ................................................................................
  consensus/80%                      ................................................................................
  consensus/70%                      ................................................................................

                    cov    pid 7521           .         .         :         .         .         .         .         6 7600
1 WP_004013458.1 100.0% 100.0%       HETSEQQGDGYQWFKVSSNYGSFQVEKTVENGAGLSENVSRQLPKTFKFEYEAKLPAGALLKPGTQPKGEFTLTVDSSNP
2 WP_101929469.1  37.0%  15.7%       --------------------------------------------------------------------------------
  consensus/100%                     ................................................................................
  consensus/90%                      ................................................................................
  consensus/80%                      ................................................................................
  consensus/70%                      ................................................................................

                    cov    pid 7601           .         .         .         .         :         .         .         . 7680
1 WP_004013458.1 100.0% 100.0%       ALAKSPVFEGFPVGTEVAITETGVEGTMPTGATMSTTWATADGKAKTKAWSSDRKSDKVTVKIQPRGLLQVKATNHFEST
2 WP_101929469.1  37.0%  15.7%       --------------------------------------------------------------------------------
  consensus/100%                     ................................................................................
  consensus/90%                      ................................................................................
  consensus/80%                      ................................................................................
  consensus/70%                      ................................................................................

                    cov    pid 7681           .         7         .         .         .         .         :         . 7760
1 WP_004013458.1 100.0% 100.0%       YPKLATLATTVDGAKMLKPSEATPVLDTVTYSGLVADREYWLLTQLVYTDDSTPVLGADGQPLARWTKVSAGKDGQGTWV
2 WP_101929469.1  37.0%  15.7%       --------------------------------------------------------------------------------
  consensus/100%                     ................................................................................
  consensus/90%                      ................................................................................
  consensus/80%                      ................................................................................
  consensus/70%                      ................................................................................

                    cov    pid 7761           .         .         .         8         .         .         .         . 7840
1 WP_004013458.1 100.0% 100.0%       VDRENPLVVPETTDSQRDLVFFESLFEVPNTPGDAGTKPPDPTDPSNPPIVEHKDPKDPKQVVSSRPKLEMQTVATIGAD
2 WP_101929469.1  37.0%  15.7%       --------------------------------------------------------------------------------
  consensus/100%                     ................................................................................
  consensus/90%                      ................................................................................
  consensus/80%                      ................................................................................
  consensus/70%                      ................................................................................
```

```
                        cov     pid 7841         :       .       .       .       .       9       .       . 7920
1 WP_004013458.1 100.0% 100.0%      VKTIKPGEAVKITDTVSYNGLKAGGVYTLVGKLVRKTDGEDVSTPVTKTGLVADASGSGKWTMDIPLTAEQTKNLKQGDE
2 WP_101929469.1  37.0%  15.7%      --------------------------------------------------------------------------------
  consensus/100%                    ................................................................................
  consensus/90%                     ................................................................................
  consensus/80%                     ................................................................................
  consensus/70%                     ................................................................................

                        cov     pid 7921         .       .       :       .       .       .       .       0 8000
1 WP_004013458.1 100.0% 100.0%      LVVFEKAYLGKMEDAASNPNLKPILAHEDFKDAGQTVTVVETPDTPPTTPPTTPPYTTPPVSPSTTTPPTTPPNPPVSPS
2 WP_101929469.1  37.0%  15.7%      --------------------------------------------------------------------------------
  consensus/100%                    ................................................................................
  consensus/90%                     ................................................................................
  consensus/80%                     ................................................................................
  consensus/70%                     ................................................................................

                        cov     pid 8001         .       .       .       .       :       .       ] 8069
1 WP_004013458.1 100.0% 100.0%      ITTPPTTPPPPAPPVAPATTIPPAQAKMPPTLARTGAQAALVGMLSLAMIAAGGAIGLLAARRKRETTE
2 WP_101929469.1  37.0%  15.7%      ---------------------------------------------------------------------
  consensus/100%                    .....................................................................
  consensus/90%                     .....................................................................
  consensus/80%                     .....................................................................
  consensus/70%                     .....................................................................
```

MView (https://desmid.github.io/mview/) 1.63, Copyright © 1997-2018 Nigel P. Brown (mailto:biomview@gmail.com)

Figure S9 Pairwise sequence alignment between *M. mulieris* and *V. cambriense* mega-adhesin. Sequences were aligned using Clustal Omega and visualised using MView.
